# High Cytotoxicity of Ruthenium(II) and Gold(I) Bimetallic Complexes and Its Precursors

**DOI:** 10.1021/acs.inorgchem.5c04993

**Published:** 2026-01-06

**Authors:** Janine Blignaut, Hendrik G. Visser, Eleanor Fourie, Marietjie Schutte-Smith

**Affiliations:** 37702 University of the Free State, Department of Chemistry, Nelson Manda Drive, Bloemfontein, South Africa 9301

## Abstract

A series of Ru­(II)-PC_(*n*)_P–Au­(I) (*n* = 1–5) bimetallic complexes were successfully synthesized and characterized with phosphorus (^31^P­{H}) and proton (^1^H) nuclear magnetic resonance (NMR) spectroscopy, electrospray ionization mass spectrometry (ESI-MS), supported by crystallographic data. Correlations between resonance shifts and P–Au­(I) bond distances provided insight into the electronic environment of the phosphorus centers, while single-crystal X-ray diffraction confirmed the molecular structures. Hirshfeld surface analysis revealed dominant H···H and F···H/H···F interactions, with contact percentages varying systematically with carbon chain length. Biological evaluation showed that the complexes exhibited higher cytotoxicity than cisplatin and melphalan against HeLa and Caco2 cancer cell lines, with pincer ligand carbon chain-length-dependent activity: shorter chains were more potent in HeLa cells, whereas longer chains were more effective in Caco2 cells. Most complexes displayed cytostatic activity, with compound **2b** demonstrating the best overall performance, combining strong cytostatic properties with high selectivity toward cancerous cells. These findings highlight the structural, electronic, and biological relationships of the complexes, underlining their potential as anticancer agents.

## Graphical abstract

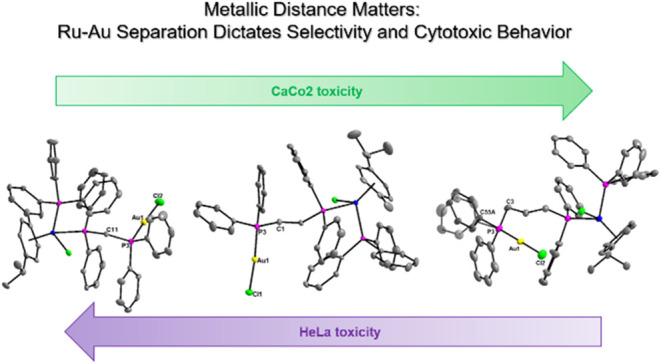

## Introduction

The challenges and successes with platinum-based drugs for the treatment of cancer are well-documented^1–4^ and have opened the door for developing alternative mononuclear, heteronuclear and multinuclear transition metal complexes as possible substitutes with lower toxicity, targeting efficiency, and efficacy against acquired drug resistance.^5^ Among these, ruthenium and gold complexes have been extensively studied as nonplatinum-based anticancer metallodrugs for their clinical potential and mechanism of action.^6–9^ The success of Rapta-C and its derivatives are well documented,^10–12^ with some displaying greater toxicity than the platinum-based anticancer drugs.^13^ Similarly, auranofin^8,14–17^ increased the interest of Au­(I)-phosphine complexes as anticancer drugs. Until now, four ruthenium complexes have been clinically tested or are in the process of being tested.^6^ On the other hand, gold (Au­(I)) complexes have also been identified as potential antiproliferative agents due to their mechanism of action.^8,9,18–20^ Additionally, Au­(III) is isoelectronic to Pt­(II) and could be seen as a possible replacement for cisplatin.^21–28^

Of all the different strategies for cancer drug design, heterometallic complexes have gained much attention, as they can incorporate the unique properties of two metals into a single structure. Different metal complexes have varying biotargets and mechanisms of action, therefore, heteronuclear complexes are expected to be multitargeting and display synergistic mechanisms of action. Also, if a specific cancer type develops resistance against one metal, the presence of a second or third metal may still exhibit activity.^27,29–34^ In many cases, compared to their parental mononuclear counterparts, heterometallic complexes exhibit increased anticancer activity and greater potency in reducing drug resistance.^35–37^ Apart from chemotherapy, heterometallic drugs offer a range of other potential applications in medicine, such as cellular imaging, trackable probes, and drug carriers for cytotoxic complexes. Various heterometallic complexes with photophysical characteristics have been evaluated for applications such as cellular imaging, trackable probes, and as drug carriers.^38^ Ferrocenyl, titanocene, and ruthenium­(II) arene complexes are highly adaptable building blocks for heterometallic complexes because of their excellent redox properties^12,31,34,35,39^ and ease of functionalization.

In spite of the attention given to Rapta-C and auranofin, very little work has been done on their heterometallic complexes or derivations thereof. Elie et al. synthesized [(η^6^-*p*-Cymene)­RuCl_2_(μ-dppm)­Au­(IMes)]­ClO_4_ (RANCE-1, dppm = diphenylphosphanyl-methyl­(diphenyl)­phosphane; IMes = 1,3-*bis*(2,4,6-trimethylphenyl)­imidazole-2-ylidene) which exhibited more efficient inhibition of thioredoxin reductase (TrxR) and similar antiproliferative properties against the renal cancer cell line (Caki-1) to auranofin.^40^ Massai et al. investigated two Ru­(II)–Au­(I) complexes, [Ru­(*p*-cymene)­Cl_2_(μ-dppm)­AuCl] and [Ru­(*p*-cymene)­Cl_2_(μ-dppm)­Au­(S-thiazoline)] (dppm = 1,1-*bis*(diphenylphosphino)-methane), that displayed an increased selectivity and cytotoxicity compared to its precursors.^18^ In a later study, the introduction of heterocyclic carbene ligands to the gold center enhanced tumor cell selectivity.^41,42^ More recently, a series of Rapta-auranofin-inspired complexes were synthesized with varying lengths of linkers between them. These complexes did not show selectivity toward cancer cells but were able to overcome platinum resistance in A2780cisR cell lines.^12^ There are a few other examples of Ru­(II)–Au­(I) heterometallic complexes that deviate somewhat from the Rapta-auranofin scaffold. Wenzel et al. combined a Ru­(II)­(polypyridine) moiety with an Au­(I)­(thioglucose) fragment with stronger antiproliferative activity against A2780 and A549 cells compared to cisplatin.^43^ Bertrand et al. synthesized heterobimetallic complexes based on the N-heterocyclic carbene-gold scaffold (Au-NHC). Interestingly, the Au-NHC-Ru­(II)­(cymene) was less cytotoxic than Au-NHC itself with IC_30_ > 100 μM in all cell lines tested.^44^

Combinations of gold with phosphine ligands have generated significant interest due to their wide range of anticancer properties.^24,45,46^ From this group, bridged digold phosphine complexes have been studied extensively.^47–52^ One such study was done by Mirabelli et al., where *bis*phosphine (PC_(*n*)_P) gold complexes were synthesized.^53^ They studied the correlation between the carbon chain length of the PC_(*n*)_P ligands and the antitumor activity of the complexes. Their findings showed good anticancer activities and that the phosphine atoms on either side of the ‘bridge’ should be close to one another to improve anticancer activity. However, according to our knowledge, a series of varying carbon chain lengths of *bis*phosphine ligands have not yet been used as bridges between Ru­(II) and Au­(I) fragments.

In this study, we report the synthesis, characterization and cytotoxic properties of a series of complexes with varying carbon chain lengths between the *bis*phosphine bridging ligands. The precursors: **1a**, **2a, 3a**, **4a**, together with the resultant bimetallic complexes: **1b**, **2b**, **3b**, **4b**, synthesized and characterized in this study is shown in Figure 1. Furthermore, the synthesis, characterization and cytotoxicity of a trimetallic Ru­(II)–Au­(I)–Au­(I) complex using *bis*(diphenylphosphinoethyl)­phenylphosphine as a bridging ligand, **5b**, and its precursor, **5a**, was also investigated (Figure 1). The crystal structures of **1a**, **1b**, **2b**, **3b**, are also reported. A counterion exchange was done to replace the SbF_6_^–^ ion with an NO_3_^–^ in **3b** to yield Ru­(II)-PC_(3)_P–Au­(I)·NO_3_ (**3c**), to investigate the effect of a counterion on the properties of the complex. Cytotoxicities of the compounds (**1a**–**5a** and **1b**–**5b**) were tested against HeLa cells and Caco2 cells to obtain the compounds’ IC_50_ values, using the SRB and MTT assays, respectively. The IC_50_ concentrations of the compounds were screened against Vero Cells that are in two different growth phases (proliferating and nonproliferating Vero cells). This was done to investigate the effects of the compounds on cells that represent nontumorigenic tissue (nonproliferating Vero cells) and tumorgenic tissue (proliferating Vero cells), using the Hoechst 33342/Propidium iodide (PI) dual staining method.

**Figure 1:**
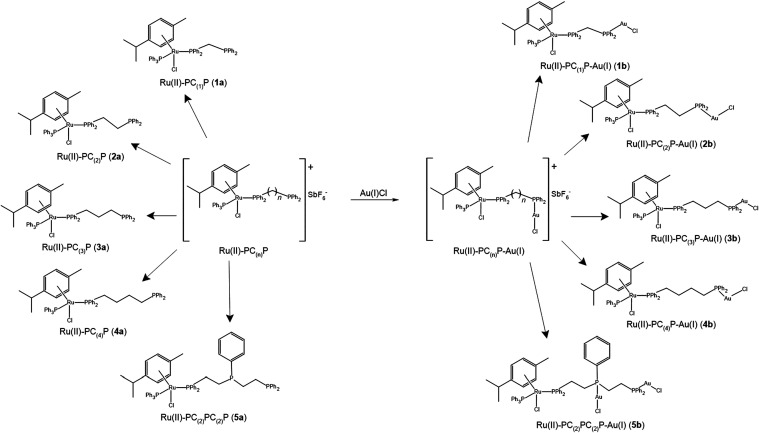
Reaction scheme showing the monometallic precursors (**1a**-**5a**) and the bridged, bimetallic complexes (**1b**-**5b**) synthesized in this study.

## Results and Discussion

### Complex Synthesis and Characterization

Ru­(II)-PC_(*n*)_P–Au­(I), where *n* = 1–4 (**1b**-**4b**), and Ru­(II)-PC_(2)_PC_(2)_P–Au­(I) (**5b**), and the precursors were successfully synthesized to investigate the anticancer properties. Here, we investigate the effect of the carbon chain length of bridging PC_(*n*)_P ligands on the anticancer properties of heterobimetallic complexes; the Ru­(II)-PC_(*n*)_P–Au­(I) complexes and the effect of adding a different metal center (Ru­(II)) to a complex containing two Au­(I) centers; the Ru­(II)-PC_(2)_PC_(2)_P–Au­(I) complex. The effect of a counterion on the properties of one metal complex was tested by exchanging the SbF_6_^–^ anion in **3b** with NO_3_^–^, to yield **3c**.

The ^1^H NMR spectra for compounds **3a** and **3b** are presented alongside their corresponding molecular structures, with colored markers used to assign each proton to its respective peak in Figure 2, confirming the purity of these samples. The full ^1^H NMR spectra for these two compounds, as well as those of the remaining complexes, are provided in the Supporting Information (Figures S2–S14). The purity was further confirmed with ^31^P­{H} NMR (Figures S15–S27), elemental analysis (Supporting Information, paragraph A1), and ESI-MS (Figures S28–S38).

**Figure 2:**
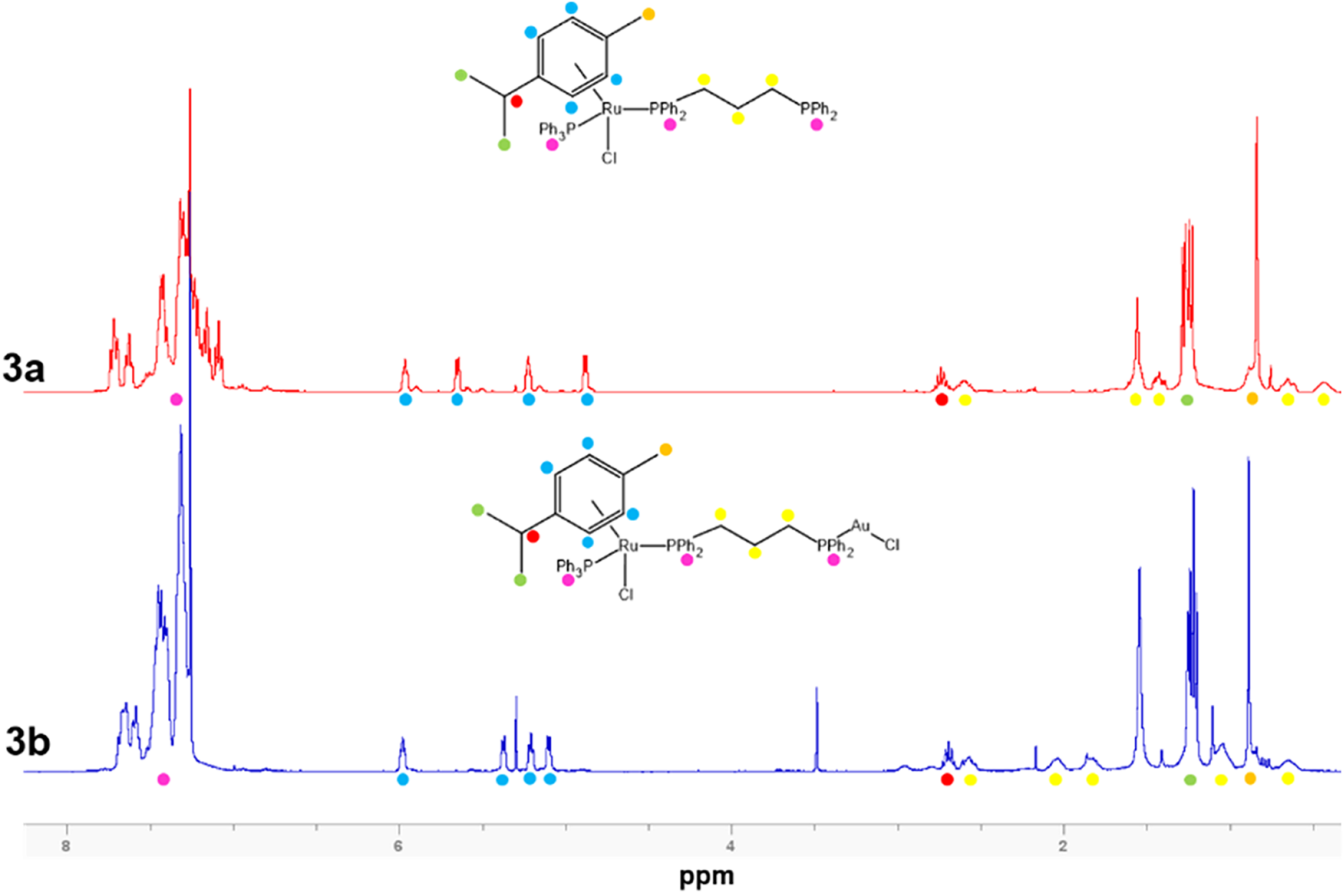
^1^H NMR spectra of **3a** and **3b**.

For the synthesis of **1b**-**5b**, the specially designed Ru­(II)-acetonitrile precursor (see Figure S1) was used, to ensure that only one phosphorus atom from the PC_(*n*)_P (*n* = 1–4) and PC_(2)_PC_(2)_P ligands bind to the ruthenium metal in a η^1^-coordination mode. Ru­(II)-acetonitrile was reacted with the five different ligands to obtain **1a**-**5a**, as illustrated in Figure 1, with 87.0, 86.2, 84.7, 82.1, and 87.4% yields, respectively. These structures were confirmed with ^31^P­{^1^H} NMR data with three distinct ^31^P­{^1^H} resonances that have large *J*_pp_ coupling constants (ca. 27–53 Hz) for the resonances belonging to the coordinated phosphorus atoms.^18^ Complexes **1b**-**5b** were then synthesized with 79.8, 80.6, 78.6, 63.4, and 86.9% yields, respectively, and also confirmed with ^31^P­{^1^H} NMR spectra. Complexes **1a**, **1b**, **2b**, and **3b** were characterized further with single crystal X-ray diffraction analysis. As the ^31^P­{^1^H} NMR data of **5b** cannot conclusively confirm the correct structure of the complex, it was subjected to ESI-MS analysis.

The ^31^P­{^1^H} NMR data of **1a**–**5a** in Table S1, informs on the coordination mode of the *bis*phosphine ligands, the environment of the P atoms, and about the Ru­(II)-P distances. The η^1^-coordination mode of these ligands is clearly shown by ^31^P­{^1^H} NMR data as each phosphorus center from the coordinated ligand has its own resonance signal (see Figure 3). In general, for the precursors, the triphenylphosphine (Ru-**P**Ph_3_) and the *bis*phosphine ruthenium-coordinated phosphine atoms (indicated as Ru-PC_(*n*)_P) resonances appear at ∼23 ppm and between 23–18 ppm, respectively, as doublets. The resonance signals of the pendant phosphorus atoms (Ru-PC_(*n*)_ ***P***) all appear below 0 ppm, as illustrated in Figure 3, as the nuclei are shielded by its electrons. Resonances for **5a** appear as two multiplets from 24.2 to 18.7 ppm, and from −12.7 to −16.2 ppm with integrals of 1:1.3, respectively, indicating that each multiplet represents two overlapping resonances.

**Figure 3:**
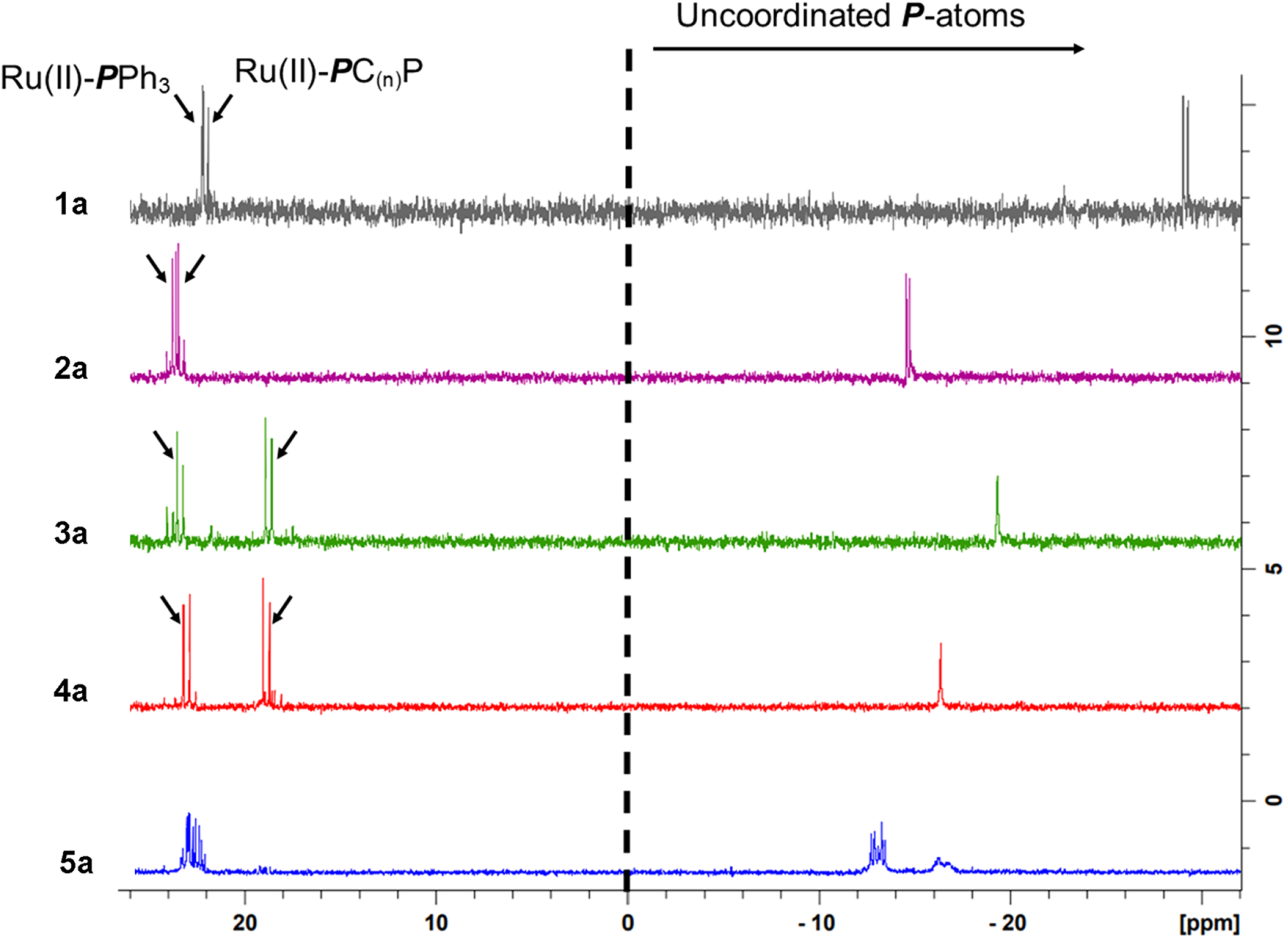
^31^P­{^1^H} NMR shifts for **1a**, **2a**, **3a**, **4a**, and **5a**.

The ^31^P­{^1^H} NMR spectra for each Ru­(II)-PC_(*n*)_P–Au­(I) complex (**1b**-**4b**) (Table S1) again show three ^31^P­{^1^H} resonances that supports the bridging η^1^-coordination mode of the PC_(*n*)_P ligands to the two metal centers. When the Au­(I)Cl is coordinated to the pendant phosphorus atom, its resonance signal no longer appears below 0 ppm, instead a new doublet/broad singlet appears between 20–32 ppm (Figure 4). The ^31^P­{^1^H} NMR data for **5b** shows four different resonances for the four different phosphorus environments in the complex. The resonance of the pendant gold-coordinated phosphorus atom appears at 34.1–33.1 ppm and the intermediate gold-coordinated phosphorus atom has a resonance at 31.8–29.7 ppm. Two more multiplets are observed at 22.9–22.7 ppm and 23.9–23.5 ppm. Since ESI-MS analysis (see Supporting Information A4) confirms the successful synthesis of **5b**, the broad multiplet ^31^P­{^1^H} resonances for this complex suggests the occurrence of certain dynamic processes in solution, causing peak overlap.^54^ When looking at the separation of the phosphorus resonances for the ruthenium-coordinated and the gold-coordinated phosphorus atoms, a direct correlation is seen: with an increase in the PC_(*n*)_P bridge length, the difference in Ru–P and Au–P ppm shifts become larger. This is indicative of an increasing change in the phosphorus environments as the distance between them and the two metal centers increase.

**Figure 4:**
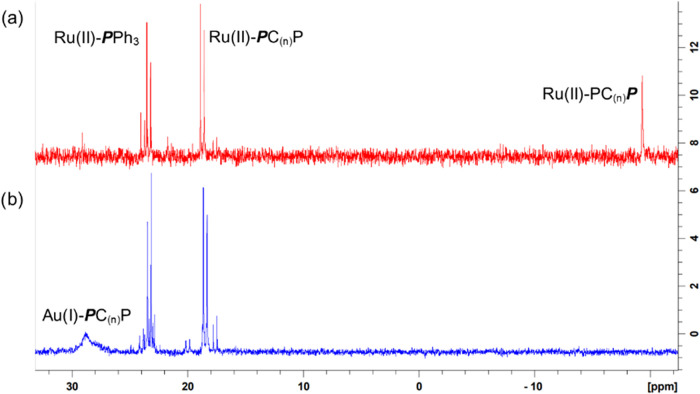
^31^P­{H} NMR resonance for Ru­(II)-PC_(3)_P (a) and Ru­(II)-PC_(3)_P–Au­(I) (b).

The *bis*phosphine metal coordinated phosphorus coupling constant of **3b** and **3c** changes from 52.3 to 135.9 Hz with a change in counterion (SbF_6_^–^ to NO_3_^–^). The magnitude of ^31^P­{^1^H} NMR coupling constants in metal–phosphine complexes is sensitive to both the coordinating ability and steric profile of the counteranion. Nitrate (NO_3_^–^), a relatively small and moderately coordinating counteranion,^55,56^ can readily approach or interact with the metal center with its oxygen atoms, altering its electron density and the Ru­(II)–P orbital interactions. Additionally, its interaction mode (i.e., monodentate or aniso-/isobidentate) with the metal center will determine how well it delocalizes the positive charge on it.^57^ In contrast, hexafluoroantimonate (SbF_6_^–^) is both weakly coordinating and sterically bulky; its diffuse charge and large ionic radius hinder close approach to the cation, restricting inner-sphere interactions.^58–60^ As a result, SbF_6_^–^ generally exerts minimal structural and electronic perturbation.

### X-ray Diffraction

The geometric parameters, basic crystallographic data, and data collection parameters of **1a**, **1b**, **2b**, and **3b** are summarized in Table 2. Selected angles and bond lengths for the complexes are listed in Table 1. The molecular diagrams of each complex are shown in Figure 5. The *p-*cymene group coordinates to Ru­(II) through a η^6^ π-bond, forming a *pseudo*-octahedral geometry or a ‘piano-stool’ configuration.^18,61–64^ The angles around the Ru­(II) center for **1a** are reported as 128.44(4)°, 125.53(4)°, and 122.76(4)° for Cg1–Ru1-P1, Cg1–Ru1-P2, and Cg1–Ru1-Cl1 (where Cg1 = center of gravity of *p*-cymene moiety), respectively, with a Ru-*p*-cymene bond of 1.7835 Å. The angles and bond lengths compare well with similar structures in literature.^63^ For **1b** they are 125.46(8)°, 129.9(7)°, and 119.46(8)°, respectively, with a slight increase in the Ru-*p*-cymene bond of 1.7892 Å. The presence of Au­(I)-Cl enlarges the Cg1-Ru-PC_(*n*)_P angle and reduces the other angles. All the bond distances and angles for **1b** compare well to similar structures in literature, with P3–Au1 and Au1–Cl2 bond distances reported as 2.220(3) Å and 2.287(3) Å, respectively, and the P3–Au1–Cl2 angle almost linear at 174.77(11)°.^18^ As the carbon chain length increases and steric influence reduces in the complexes, the Cg1-Ru-PC_(*n*)_P and Cg1–Ru-Cl angles seem to adjust itself to reach angles of similar magnitude, where **2b** have the largest angles for all three angles with the longest Ru-*p*-cymene bond. The effect of the presence of Au­(I)-Cl can be seen in the *bis*phosphine phosphorus atoms’ bond lengths in **1a** and **1b**, as the Ru-PC_(*n*)_P and C–P­(Au) bond lengths decrease; from 2.386 and 1.877 Å in **1a** to 2.366 and 1.830 Å in **1b**. The same bonds decrease as the two metal centers are bridged by longer carbon chains. The Ru-*p*-cymene bond length shows a slight increase, where the longest bond appears in **2b**. The longest Ru–Cl bond, P–Au bond, and Au–Cl also belong to **2b**. With an increase in carbon chain length, the P3–Au1–Cl1 angle adjusts to values closer to 180.0° where the angles for **2b** and **3b** are 175.12(5)° and 178.5(2)°.

**Figure 5:**
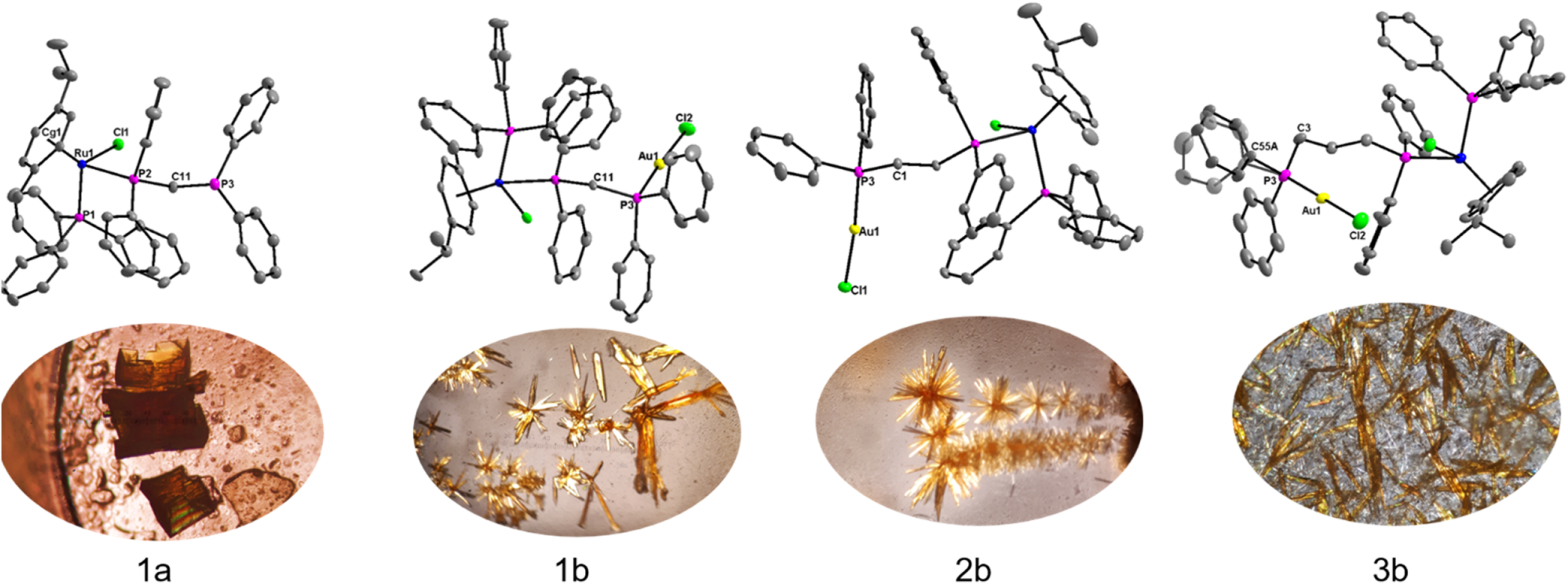
Molecular diagrams and pictures of each complex’s crystals for **1a**, **1b**, **2b**, and **3b**. All diagrams indicate atom numbering schemes. Hydrogen atoms and insignificant labels were omitted for clarity. Ellipsoids are drawn at 50%.

**Table 1:** Selected Bond Lengths­(Å) and Angles (°) for **1a**, **1b**, **2b**, and **3b**

	**1a**	**1b**	**2b**	**3b**
Ru-PPh_3_	2.365(2)	2.367(3)	2.337(1)	2.366(3)
Ru-PC_(*n*)_P	2.386(2)	2.366(3)	2.364(1)	2.358(3)
Ru-*p*-cymene	1.7835(6)	1.7892(8)	1.7960(6)	1.795(1)
Ru–Cl	2.392(1)	2.392(3)	2.396(1)	2.389(3)
C–P(Au)	1.877(5)	1.83(1)	1.827(5)	1.81(1)
P–Au	-	2.220(3)	2.232(2)	2.226(4)
Au–Cl	-	2.287(3)	2.293(1)	2.267(4)
Cg1-Ru-PPh3	128.44(4)	125.46(8)	125.70(5)	125.16(9)
Cg1-Ru-PC_(*n*)_P	125.53(4)	129.9(7)	126.76(4)	125.67(8)
Cg1–Ru-Cl	122.76(4)	119.46(8)	123.18(4)	123.03(9)

**Table 2:** Summary of the Crystal Data of **1a**, **1b**, **2b**, and **3b**

	**1a**	**1b**	**2b**	**3b**
Empirical formula	C_54_H_53_Cl_3_F_6_P_3_RuSb	C_54_H_53_AuCl_4_F_6_P_3_RuSb	C_54_H_53_AuCl_2_F_6_P_3_RuSb	C_55_H_55_AuCl_2_F_6_P_3_RuSb
Formula weight	1238.05	1470.46	1399.56	1413.58
Temperature (K)	100.0	100.0	100.0	100.0
Crystal system	Monoclinic	Triclinic	Triclinic	Triclinic
Space group	*P*2_1_/*n*	*P*1̅	*P*1̅	*P*1
Unit cell dimensions				
*a* (Å)	13.801(5)	9.9637(9)	11.514(3)	9.173(4)
*b* (Å)	18.738(6)	21.138(2)	13.017(3)	12.069(5)
*c* (Å)	21.213(7)	25.526(3)	19.197(4)	13.871(6)
α (°)	90	86.644(3)	73.292(8)	68.110(13)
β (°)	107.905(11)	89.047(3)	83.292(8)	71.092(12)
γ (°)	90	76.835(3)	65.258(8)	72.078(13)
Volume (Å^3^)	5220(3)	5225.8(9)	2502.7(10)	1317.1(9)
*Z*	4	4	2	1
Density (g/cm^3^)	1.575	1.869	1.857	1.782
Absorption coefficient (mm^–1^)	1.110	3.955	4.020	3.821
*F*(000)	1488.0	2872.0	1368.0	692.0
Crystal size (mm^3^)	0.234 × 0.124 × 0.064 mm^3^	0.095 × 0.035 × 0.013	0.21 × 0.143 × 0.042	0.122 × 0.041 × 0.037
Radiation	Mo Kα (λ = 0.71073)	Mo Kα (λ = 0.71073)	Mo Kα (λ = 0.71073)	Mo Kα (λ = 0.71073)
Range of data collection (°)	5.342 to 55.998	3.666 to 55.996	3.572 to 56.878	5.126 to 55.992
Index range	–18 ≤ *h* ≤ 18	–13 ≤ *h* ≤ 13	–15 ≤ *h* ≤ 15	–12 ≤ *h* ≤ 12
	–22 ≤ *k* ≤ 24	–27 ≤ *k* ≤ 27	–17 ≤ *k* ≤ 17	–15 ≤ *k* ≤ 15
	–28 ≤ *l* ≤ 24	–33 ≤ *l* ≤ 33	–25 ≤ *l* ≤ 25	–18 ≤ *l* ≤ 18
Reflections collected	53418	113703	83697	21835
Independent reflections	12591 [*R*_int_ = 0.0953, *R*_sigma_ = 0.0923]	23757 [*R*_int_ = 0.2094, *R*_sigma_ = 0.1315]	12550 [*R*_int_ = 0.0722, *R*_sigma_ = 0.0416]	10231 [*R*_int_ = 0.0474, *R*_sigma_ = 0.0938]
Completeness (°,%)	27.999°, 99.9	27.998, 94.2	28.439, 99.4	27.996, 97.0
Refinement method	Full-matrix least-squares on *F* ^2^			
Data/restraints/parameters	12591/0/556	23757/0/1150	12550/0/580	10231/3/542
Goodness-of-fit on *F* ^2^	1.107	1.104	1.038	1.073
Final *R* indices [*I* > 2sigma(*I*)]	*R*_1_ = 0.0696	*R*_1_ = 0.0825	*R*_1_ = 0.0497	*R*_1_ = 0.0486
	w*R*_2_ = 0.1066	w*R*_2_ = 0.1491	w*R*_2_ = 0.1291	w*R*_2_ = 0.0960
Final *R* indices (all data)	*R*_1_ = 0.1068	*R*_1_ = 0.1202	*R*_1_ = 0.0527	*R*_1_ = 0.0590
	w*R*_2_ = 0.1165	w*R*_2_ = 0.1626	w*R*_2_ = 0.1314	w*R*_2_ = 0.1009
Largest diff. peak/hole (Å^‑3^)	1.12/–1.19	1.75/–2.38	12.98/–2.68	1.89/–2.18
Flack parameter	-	-	-	0.055(6)

Nine hydrogen bonding and seven π-interactions are observed in the structure of **1a** (see Table S3 and Figures S40 and S41). The hydrogen bonding and π-interactions range between 3.124(7) Å and 3.437(6) Å (D···A) and between 3.415(6) Å and 3.886(6) Å (D···Cg) respectively. Interestingly, eight of the nine hydrogen bonding interactions observed in **1a** involves the SbF_6_^–^ counterion and DCM solvent molecule. Five of the seven aromatic rings, all three aromatic rings from the PPh_3_ moiety and one from the PPh_2_ moiety, are involved in the π-interactions. **1a** is further stabilized by bifurcation between Cl1 and two hydrogen atoms (H11A and H25). Molecules of **1a** pack in a head-to-tail fashion when viewed along the *b*-axis (Figure S42). Fourteen hydrogen bonding interactions (of which eight are intramolecular), seven D-H···π interactions (of with five are intramolecular), two D-X···π interactions, and two π–π interactions are observed in the structure of **1b** (Table S5 and Figures S46 and S47). All 14 of the hydrogen bonding interactions involve the counterion or solvent molecule. **1b** is further stabilized by bifurcation at Cl1 (from H11B and H29) and Cl3 (from H110B and H84), and an intramolecular short contact (4.153(4) Å) between Sb1 and Cl6 forming an infinite chain along the *c*-axis (Figure S48). **1b** packs in a head-to-head fashion in column like structures with the counterions and solvent molecules systematically positioned in between the columns, when viewed along the *b*,*c*-plane (Figure S48). Twelve hydrogen bonding interactions, of which six are intramolecular (Figure S52), and three intramolecular C–H···π-interactions (Figure S53) are observed in the structure of **2b**. The ruthenium bound chlorido ligand is the acceptor of five of the six intramolecular hydrogen bonding interactions, significantly stabilizing the crystal structure of **2b** (Table S7). **2b** packs in a head-to-head fashion with the SbF_6_^–^ counterions in between, when viewed along the *a*-axis (Figure S54). Five hydrogen bonding interactions and seven π-interactions are observed in the structure of **3b** (Figures S58 and S59, Table S9). Three of the hydrogen bonding interactions are to three SbF_6_^–^ counterions (one intramolecular and two intermolecular), while Cl1 is once again the acceptor of two intramolecular hydrogen bonding interactions. All of this contributes to the stabilization of **3b**. The molecules pack in a head-to-toe fashion along the *b*-axis, with the Ru­(II) center of both molecules (created by a glide plane along the *b*-axis) situated at a corner of the unit cell and the counterions forming “layers” in between the complex molecules (Figure S60).

With the addition of Au­(I)Cl to **1a**, to form **1b**, the ^31^P­{^1^H} shifts of the ruthenium-coordinated and the pendant phosphorus atoms have marked changes. The ruthenium-coordinated phosphorus has a 3 ppm upfield shift. This may be explained by solid-state bond distances. The bond distances of this ruthenium-coordinated phosphorus between the ruthenium and the carbon atoms (see Ru–P2 and P2–C11 in Tables S2 and S4) changes from 2.386 to 2.366 Å and 1.838 to 1.830 Å. These closer associations change the electron density on the phosphorus atom, resulting in the shift. The increase in electron density results in a resonance appearing as a doublet of doublets, coupling with the triphenylphosphine, and a weaker coupling to the gold-coordinated phosphorus.

For **2b** and **3b** only, the pendant phosphorus shifts changed significantly. From solid-state data, the P–Au bond lengths in decreasing order, 2.232 > 2.226 > 2.220 Å, correlate with the decreasing trend of their NMR shifts of 30.10 > 28.83 > 20.34 ppm for complexes in the order **2b**, **3b**, and **1b**, respectively. This trend shows that gold-coordinated phosphorus NMR shifts depend on its association with the gold­(I) metal center. The NMR data, coupled with the solid-state crystal data shows **2b** has the longer P–Au bond length resulting in the more downfield NMR shift. Additional solid-state data shows that **2b** has the longest Au–Cl bond length, and the shortest Ru-PPh_3_ bond length of the bimetallic crystals. This may indicate that the *bis*phosphine ligand of **2b** creates a conformation which sterically allows the triphenylphosphine to have a closer association with the ruthenium, allowing the Au–Cl bond to increase. The slight increase in the P–Au bond length may indicate that this conformational change may be facilitated by the positive inductive effect of the *bis*phosphine ligand and the σ-donating ability of phosphorus. Figure S59 shows that in solid state, the SbF_6_^–^ counterion interacts through π-interactions with the *p*-cymene moiety, which stabilizes a charged metal center through electron/charge transfer.^65^ Research has also linked coupling constant variation to changes in geometry of the metal center.^66^ This drastic change in counterion for complex **3c**, may influence the *pseudo*-octahedral geometry of the Ru­(II) center as well. The aforementioned factors cause the redistribution of electron density which enhances the phosphorus scalar coupling, resulting in larger ^31^P­{^1^H} couplings Therefore, the complex paired with SbF_6_^–^ (**3b**) typically displays a smaller phosphorus coupling constant than its NO_3_^–^ analogue (**3c**) due to possible electronic influences on the Ru­(II) center.

### Hirshfeld Analysis

Bifurcation is observed at the Ru-chlorido ligand in all four structures; two interactions in **1a**, four interactions in **1b** (two for each complex molecule), five interactions in **2b**, and two interactions in **3b**. All the structures show hydrogen bonding interactions that involve the SbF_6_^–^ counterion, which stabilizes the crystal packing; six interactions (two intramolecular) in **1a**, eight (four intramolecular) in **1b**, six (one intramolecular) in **2b**, and three (one intramolecular) in **3b**. Fingerprint plots of the crystal structures of **1a**, **1b**, **2b**, and **3b** were obtained and are illustrated in Figures S43, S48, S54, and S60 highlighting the particular atom pair close contacts. The decomposed fingerprint plots for each type of interaction have the same pattern for all four structures with the C···H/H···C interactions comprising between 12.7 and 17.7%, the Cl···H/H···Cl interactions comprising between 7.3 and 8.7% (with **1b** being an outlier at 24.4%), the F···H/H···F interactions comprising between 17.4 and 20.9%, and H···H comprising between 49.2 and 54.5% (with **1b** being an outlier at 40.5%) of the total Hirshfeld surface area. The H···H contacts contribute the most to the crystal packing of all the structures, while another substantial amount of stabilization comes from the F···H/H···F interactions in all the structures and the Cl···H/H···Cl interactions in **1b** (24.4%) (Figure 6). The C–H···π interactions are seen in the fingerprint plots as characteristic ‘wings’, more prominent in **1b** (Figure S49) where 11 π-interactions are observed. When d_norm_ is mapped on a Hirshfeld surface, intermolecular contacts, defined as contacts shorter than the sum of the van der Waals radii, appear as red spots on a blue surface, another valuable method to identify close intermolecular contacts. The visible contacts in the d_norm_ Hirshfeld plots of all the structures (Figures S44, S50, S56, and S62) correlates with the interactions summarized in Tables S3, S5, S7, and S9. It is interesting to note that with an increase in carbon chain length of the *bis*phosphine ligand between the two metal centers, Ru­(II) and Au­(I), H···H contacts and C···H/H···C contacts increase (Figure 6), however, Cl···H/H···Cl contacts decrease. The impact of Au­(I) on the Hirschfield plots can be seen when comparing contact percentages between **1a** and **1b**. When Au­(I) is present, C···H/H···C contacts and F···H/H···F contacts decrease. A drastic decrease in H···H contacts are observed, where in contrast a drastic increase in Cl···H/H···Cl contacts are observed.

**Figure 6:**
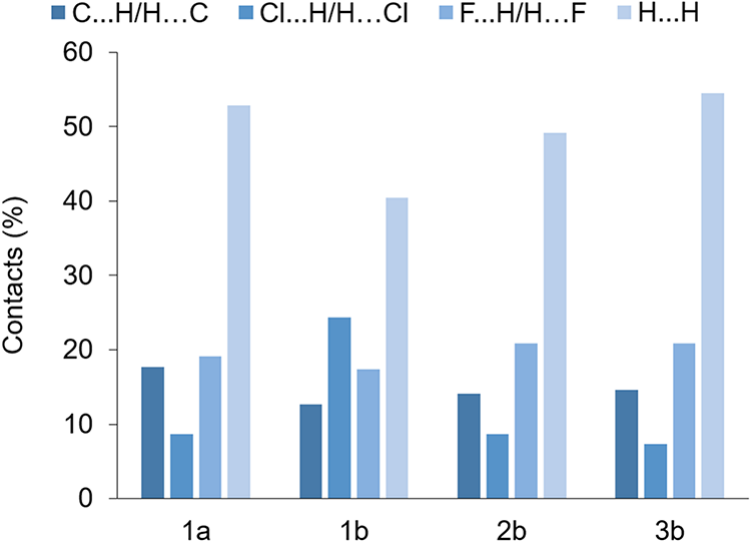
Hirshfeld contacts (%) resolved from fingerprint plots of **1a**, **1b**, **2b**, and **3b**.

### Cytotoxicity Studies

The IC_50_ value of a compound is the concentration of a compound where viable cell numbers will be reduced by 50%. These were determined on two cell lines: HeLa cells and Caco2 cells. Additionally, the complexes were screened against proliferating and nonproliferating Vero cells, testing the effect of these compounds on tumor-like tissue and nontumorigenic tissue and showing the selectivity of the compounds toward tumor-like tissue. The results of three sets of data (**1a-5a, 1b-5b**, and **3c**) are summarized in Table 3. The only difference between **1a**–**4a** and **1b**–**4b** is the carbon chain length between the two phosphorus atoms and metal centers. Complex **5a** has three phosphorus moieties instead of two and **5b** contains two P–Au­(I)-Cl moieties instead of one. Complex **3c** contains a different counterion, NO_3_^–^, where the other complexes have SbF_6_ as counterion. This allows us to see the effect of the distance between the Ru­(II) and Au­(I) centers, the effect of one and two P–Au­(I)-Cl moieties with one Ru­(II) in one complex, and the effect of the counterion my have on the anticancer properties.

**Table 3:** Cytotoxicity of the 11 Synthesized Complexes toward HeLa (SRB Assay, 72 h Incubation; IC_50_ Values, Mean ± SD, *n* = 3) and Caco-2 Cells (MTT Assay, 48 h; IC_50_ Values, Mean ± SD, *n* = 4), along with Growth-Inhibition Results Obtained in Vero Cells (MTT Assay, 48 h; IC_50_ Values, Mean ± SD, *n* = 4)

Ru(II)-PC_(*n*)_P·SbF_6_										
	HeLa		Caco2		Vero					
	IC_50_ (μM)	(±SD)	IC_50_ (μM)	(±SD)	% live nonproliferating cells	(±SD)	% live proliferating cells	(±SD)	Toxicity	Selectivity^c^
**1a**	4.20	0.21	1.70	0.03	73.76	5.55	39.21	10.95	Cytostatic	1.88
**2a**	22.19	0.19	1.54	0.03	71.64	3.41	24.53	6.72	Cytostatic	2.92
**3a**	11.07	0.16	3.98	0.03	46.26	2.85	13.38	2.51	Cytotoxic	3.46
**4a**	13.96	0.17	2.76	0.02	66.89	4.04	15.52	5.71	Cytostatic	4.31
**5a**	23.23	0.20	4.00	0.03	50.93	1.50	30.34	7.02	Cytostatic	1.68
**Control**	0.75^a^	0.28	30^b^	3.31	6.08	0.17	5.78	0.25	Cytotoxic	1.05
Ru(II)-PC_(*n*)_P–Au(I)·SbF_6_										
**1b**	0.470	1.2	6.10	0.03	46.04	2.43	25.33	2.99	Cytotoxic	1.82
**Control**	8.08^a^	1.2							Cytotoxic	5.01
**2b**	0.413	1.55	4.40	0.01	79.32	8.62	28.62	4.29	Cytostatic	2.77
**3b**	1.264	1.53	0.80	0.04	80.49	2.93	47.68	6.85	Cytostatic	1.69
**3c**	1.89	1.46	4.57	0.03	27.52	1.10	16.51	2.63	Cytotoxic	1.67
**4b**	1.072	1.45	2.10	0.04	93.74	5.45	78.60	6.32	Cytostatic	1.19
**5b**	3.391	1.62	4.80	0.01	81.13	2.87	29.97	8.92	Cytostatic	2.71
**Control**	2.97^a^	1.4	50^b^	2.93	29.98	2.73	5.99	2.99	Cytotoxic	5.01

a Cisplatin.

b Melphalan.

c Selectivity toward proliferating cells (% live nonproliferating cells/% live proliferating cells).

### Cytotoxicity against HeLa and Caco2 Cell Lines

The IC_50_ values for the Ru­(II)-PC_(*n*)_P precursors on the Caco2 cell line increase in the order **2a** < **1a** < **4a** < **3a** < **5a**. The precursors with shorter carbon chains (**2a** and **1a**, where *n* = 2 and 1, respectively) are more cytotoxic than those with the longer carbon chains. All the precursors have IC_50_ values lower than that of the positive control (Melphalan). When tested on HeLa cells, the IC_50_ values increase in the order **1a** < **3a** < **4a** < **2a** < **5a**, with all of the values being larger than that of the positive control (cisplatin). The IC_50_ values of the five Ru­(II)-PC_(*n*)_P–Au­(I) complexes (**1b-5b**) for the Caco2 cell line and the HeLa cell line, increase in the order **3b** < **4b** < **2b** < **5b** < **1b** and **2b** < **1b** < **4b** < **3b** < **5b**, respectively. The carbon chain length between the Ru­(II) and Au­(I) metal centers is observed to have an influence on the complexes’ toxicity toward both cell lines, where the longer carbon chains show greater toxicity toward Caco2 cells and the shorter carbon chains show greater cytotoxicity to the HeLa cells. This suggests different anticancer mechanisms of the complexes in the two cell lines. Most of the complexes show excellent cytotoxicity when compared to anticancer drugs currently in use (cisplatin and melphalan). The Caco2 cell line IC_50_ values of **1a** and **2a** increased from 1.7 and 1.54 μM to 6.1 and 4.4 μM, respectively, upon the addition of AuCl to the complexes to form **1b** and **2b**. The opposite is true for **3a** and **4a** (3.95 and 2.76 μM) as they become **3b** and **4b** (0.8 and 2.10 μM), as the addition of AuCl lowers their IC_50_ values. This indicates that the coordination of the Au­(I) center to the short, more rigid *bis*phosphine ligands interferes with the anticancer activity of the complexes, however, improves the anticancer activity when coordinated to the longer, more flexible *bis*phosphine ligands. The coordination of AuCl to the complexes drastically decreased the HeLa IC_50_ values by at least 10-fold, suggesting an improved anticancer activity by the heterobimetallic complexes, while the shorter bridged complexes retain the higher toxicity and the longer bridged complexes the lower toxicity. The Since the IC_50_ values of **3b** (1.264 and 0.80 μM) are much lower than that of **3c** (1.89 and 4.57 μM), it suggests that the counterion SbF_6_^–^ contributes in giving the complex a lower IC_50_ value.

Comparison of the crystallographic parameters of complexes **1b**-**3b** with their cytotoxicity profiles reveals several correlations. For HeLa cells, cytotoxicity decreases in the order **2b** > **1b** > **4b** > **3b**. Within this series, the Au–Cl bond lengths decrease in the order **2b** > **1b** > **3b**. Although the Au–Cl bond length alone cannot establish a mechanism, longer Au–Cl bonds are generally associated with weaker Au–Cl interactions, which in other gold­(I) systems have been linked to increased lability and potential for mitochondrial protein thiol association or cellular uptake.^9,64^ The data here show a correlation between longer Au–Cl bonds and higher cytotoxicity within this specific set of complexes, although the underlying mechanism has not been experimentally verified here. Mirabelli et al. reported that in related PC_(*n*)_P systems, complexes containing a two-carbon bridging ligand, displayed the highest anticancer activity which the authors attributed to conformational flexibility revealed by crystallographic studies.^53^ While their mechanistic interpretation cannot be directly transferred to our Ru–Au complexes, the trend they observed - decreasing biological activity with increasing P–P separation—is consistent with our cytotoxicity data. In our series, complexes **2b** and **1a**, which have the shortest P–P distances and closest Ru–Au proximities, also exhibit the highest activity toward HeLa cells. Taken together, these observations indicate a structure–activity correlation in which shorter P–P distances and longer Au–Cl bonds coincide with increased cytotoxicity within this family of complexes. However, in the absence of direct mechanistic studies, any mechanistic interpretation remains tentative, and our conclusions are therefore restricted to the observed structural correlations that may inform future mechanistic investigations.

Caco2 cytotoxicity decrease in the order **3b** (*n* = 3) > **4b** (*n* = 4) > **2b** (*n* = 2) > **5b** (*n* = 2) > **1b** (*n* = 1). It is clear that the shorter the carbon chain between the metal centers/phosphorus atoms become the cytotoxicity decreases. This could be explained by looking at solid-state data. The anticancer mechanism of ruthenium complexes usually involves the intercalation, electrostatic associations, and covalent bonding of its aromatic rings with DNA/proteins.^64^ Chlorido ligands are substituted by aqua ligands, activating the metal center.^67^ In the order **3b**, **2b**, and **1b** (decreasing cytotoxicity); the Ru-PC_(*n*)_P and RuPC_n_-P bond lengths increase. The shorter bonds may indicate a stronger inductive effect, increasing the reactivity of the metal centers. In the same order, Ru-*p*-cymene and Ru–Cl bond lengths decrease. The longer Ru-*p*-cymene bond may facilitate intercalation of the *p*-cymene with DNA or proteins. This can be further supported by looking at how the ‘piano stool’ conformation changes from **1b** to **2b** to **3b** (Table 2), with angles around the *p*-cymene becoming larger, resulting in less steric hindrance around it. The longer (weaker) Ru–Cl bond lengths facilitate its substitution with aqua ligands, which activates the Ru­(II) metal center for further biological reactivity.

### Screening against Vero Cells

All the compounds were screened against two groups of Vero cells, proliferating (similar to tumorigenic cells) and nonproliferating (similar to nontumorigenic cells). These two cell phases were exposed to three different concentrations (where the IC_50_ is that of the Caco2 cell line) of each compound for 48 h, after which a live cell quantification was done. The live cell data of these two sets of cells, after 48 h, are reported in Figure 7. The Ru­(II)-PC_(*n*)_P precursors showed decreasing proliferating cell inhibition in the order **3a** > **2a** > **5a** > **1a** > **4a**, where **1a**, **2a**, and **4a** have cytostatic activity and **3a** and **5a** have cytotoxic activity. The selectivities listed in Table 4 show an increase with increase in the *bis*phosphine carbon chain length. The greatest inhibitory effect on proliferating Vero cells is displayed by **1b**, **2b**, and **5b**. Compound **1b** appears to be cytotoxic, as its IC_50_ concentration inhibit proliferating Vero cell viability with >50 and ∼50% for nonproliferating Vero cells. Compounds **2b**, **3b**, **4b**, and **5b** show cytostatic activity (inhibit the growth of proliferating cells without having a large effect on nonproliferating cells) at IC_50_ concentrations. Compounds **2b**, **3b**, and **5b** inhibit proliferating cell viability by >50% and nonproliferating viability by ∼20%. Complex **4b** inhibits the viability of proliferating cells by ∼20% with no significant effect on the nonproliferating cells. Therefore, **2b**-**5b** may be less toxic to nontumorigenic cells than they will be to tumor-like tissue and displays selectivity toward tumor-like tissue. The selectivities on these bimetallic complexes toward proliferating cells are listed in Table 4. Their selectivities decrease in the order **2b** > **5b** > **1b** > **3b** > **4b** and show lower selectivity values than their precursors. This can be expected as P–Au–Cl moieties are known for its nonselective cell accumulation due to its high lipophilicity.^9^ The selectivities and cytotoxic/cytostatic activities of the complexes change with a change in the concentration administered. Thus, these complexes have tunable anticancer properties. Lastly, at their IC_50_ values, **3b** is cytostatic where **3c** is cytotoxic, however, their selectivities remain the same. This suggests that the counterion has an effect on its toxicity (NO_3_^–^ give more toxicity), but does not influence its selectivity.

**Figure 7:**
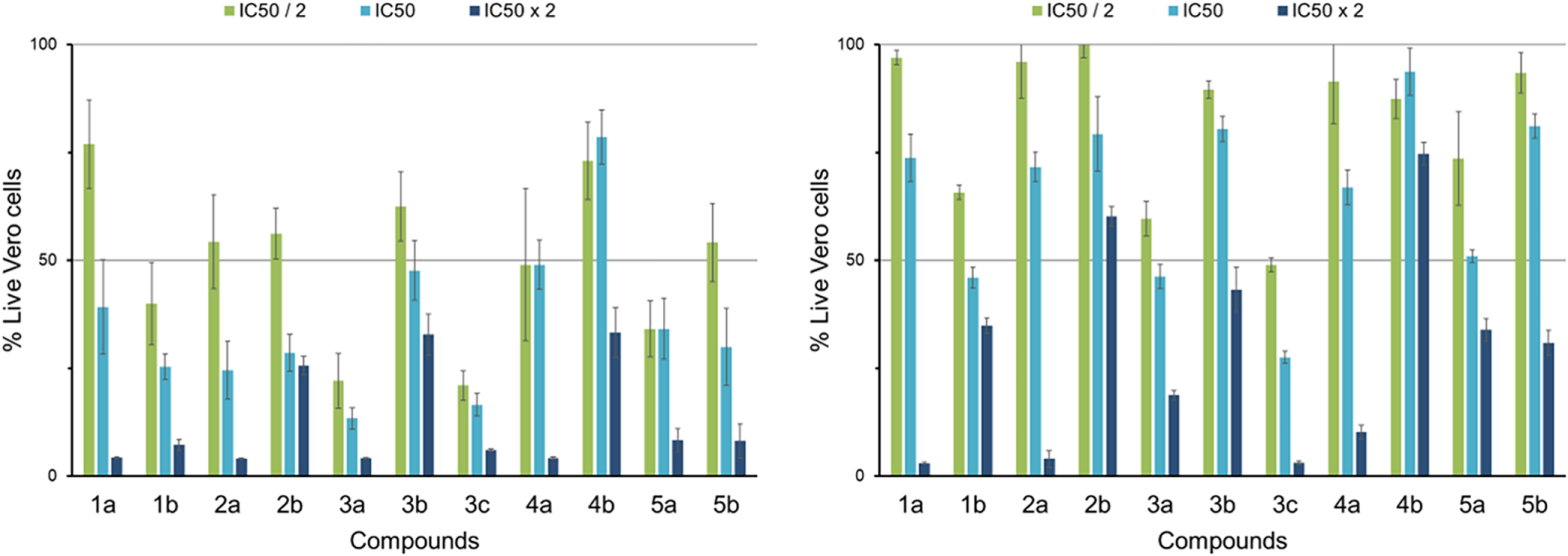
Number of live proliferating Vero cells (left) and nonproliferating Vero cells (right) after 48h treatment with three different concentrations of each complex. The values represented in this graph are the mean values of four replicates.

**Table 4:** A Summary of the Three Concentrations Used for Compounds **1**–**5**

Conc.	**1a**	**1b**	**2a**	**2b**	**3a**	**3b**	**3c**	**4a**	**4b**	**5a**	**5b**	**Melphalan**
IC_50_ / 2 (μM)	0.77	3.07	1.99	2.195	1.38	0.385	2.29	2.00	1.065	1.42	2.39	15
IC_50_ (μM)	1.54	6.14	3.98	4.39	2.76	0.77	4.57	4.00	2.13	2.83	4.78	30
IC_50_ × 2 (μM)	3.07	12.28	7.96	8.78	5.51	1.54	9.15	7.99	4.26	5.67	9.56	60

## Conclusion

Bimetallic complexes were successfully synthesized and fully characterized, with phosphorus NMR providing valuable insights into their electronic environments. Correlations between NMR chemical shifts and crystallographic bond distances, particularly the P–Au­(I) bonds, supported the interpretation of electronic effects around the phosphorus centers. Single-crystal X-ray diffraction confirmed the molecular structures and further enabled a comparison of electronic and structural parameters. Hirshfeld surface analysis revealed that intermolecular interactions, dominated by H–H and F–H/H–F contacts, varied systematically with the carbon chain length, corroborating the crystallographic data and highlighting the role of packing effects. Biological evaluation demonstrated that the complexes exhibited higher cytotoxicity than the standard drugs cisplatin and melphalan against both HeLa and Caco2 cancer cell lines. The results showed chain-length-dependent activity, with shorter-chain derivatives displaying greater potency toward HeLa cells, while longer-chain derivatives were more effective against Caco2 cells. Most of the complexes acted through cytostatic mechanisms, with compound **2b** standing out for combining strong cytostatic properties with notable selectivity toward cancerous cells. Overall, this study establishes clear structure–activity relationships across synthesis, solid-state characterization, and biological evaluation, highlighting the potential of these bimetallic complexes as promising anticancer agents.

## Experimental Section

### General Procedures

All syntheses were carried out under strict inert conditions using a Schlenk line. The solvents used were dried and distilled using standard procedures. All reagents used in the syntheses of all compounds were purchased from Sigma-Aldrich and Merck, South Africa, and used without any additional purification.

The techniques used to characterize the ligands and complexes synthesized were infrared spectroscopy (IR), ultraviolet/visible spectroscopy (UV/vis), nuclear magnetic resonance spectroscopy (NMR), and single crystal X-ray diffraction (XRD). NMR spectra were collected on a 300 MHz Bruker FOURIER NMR spectrometer operating at 25.0 °C with a 5.0 mm ^13^C/^1^H high-resolution NMR probe equipped with Z gradient coil recording ^1^H NMR spectra at 300.18 MHz. Spectra were also collected on a 400 MHz AVANCE III NMR spectrometer operating at 25.0 °C with a 5 mm BBI H-BB-D probe with Z gradients, collecting ^1^H NMR spectra at 400.13 MHz and ^31^P­{^1^H} spectra at 161.97 MHz. Chemical shifts are given in ppm and coupling constants (*J*) are given in Hz. The deuterated solvents used are indicated in the experimental procedures. Single crystal X-ray diffraction data were completed on a Bruker D8 Quest Eco Chi Photon || CPAD diffractometer and a Bruker D8 Venture 4K Kappa Photon ||| C28 diffractometer. Infrared spectra were collected on a Thermo Scientific Smart iTX at room temperature, that uses a monolithic diamond accessory, in the range of 4000 to 600 cm^–1^. Ultraviolet/visible spectra were collected on a Varian Cary 50 UV/vis Spectrophotometer at room temperature. Where electron spray ionization mass spectroscopy (ESI-MS) was done, samples were analyzed using an ABSCIEX 4000 QTRAP hybrid triple quadrupole ion trap mass spectrometer with a Shimadzu HPLC stack as a front end. All data acquisition and processing were performed using Analyst 1.5 (AB SCIEX) software. All characterization data is reported in the Supporting Information A1.

### Synthesis of Precursor Complexes

To synthesize Ru­(II)-PC_(*n*)_P complexes, the precursors η^6^ *-p-*cymenedichlorido-triphenylphosphinoruthenium­(II) (Ru­(II)-PPh_3_) and η^6^ *-p-*cymeneacetonitrilechlorido-triphenylphosphineruthenium­(II) hexafluoroantimonate­(I) (Ru­(II)-acetonitrile) were synthesized first according to the methods described in the Supporting Information A1.

### Synthesis of Ru­(II)-PC_(*n*)_P Complexes

A solution of Ru­(II)-acetonitrile (1 equiv) and the PC_(*n*)_P ligand (1 equiv) in DCM (20 mL) was stirred at room temperature for 1–2 h. The solvent was removed until ∼5.0 mL of the solvent was left. Upon addition of pentane to the leftover solvent, the product precipitated out as a yellow-orange solid. The solid was filtered and allowed to dry *in vacuo* for ∼2 h to yield complexes **1a**, **2a**, **3a**, **4a**, and **5a** (87.0, 86.2, 84.7, 82.1, and 87.4%). Crystals suitable for X-ray crystallography were obtained by recrystallizing the product in DCM/Diethyl ether [1:1]. **1a-** ^31^P­{^1^H} NMR (161.97 MHz, Chloroform-*d1*): δ 23.0 (d, *J* = 13.5 Hz), 22.9 (d, *J* = 27.8 Hz), −29.3 (dd, *J* = 27.9, 13.7 Hz). **2a-** ^31^P­{^1^H} NMR (161.97 MHz, Chloroform-*d1*): δ 23.7 (d, *J* = 30.4 Hz), 23.4 (s), −14.7 (dd. *J* = 29.3, 3.9). **3a-** ^31^P­{^1^H} NMR (161.97 MHz, Chloroform-*d1*): δ 23.4 (d, *J* = 52.2 Hz), 18.7 (d, *J* = 52.4 Hz), −19.3 (s). **4a-** ^31^P­{^1^H} NMR (161.97 MHz, Chloroform-*d1*): δ 23.0 (d, *J* = 52.7 Hz), 18.8 (d, *J* = 52.4 Hz), −16.4 (s). **5a-** ^31^P­{^1^H} NMR (161.97 MHz, Chloroform-*d1*): δ 24.2–18.7 (m), −12.7 to −16.2 (m).

### Synthesis of Ru­(II)-PC_(*n*)_P–Au­(I) Complexes

Ru­(II)-PC_(*n*)_P (1 equiv) and [AuS­(Me)_2_Cl] (1 equiv) were dissolved in dichloromethane (10.0 mL) to yield a red solution that was stirred at room temperature for 2 h. Dichloromethane was then removed under reduced pressure until ∼5.0 mL of the solvent was left. Pentane was added to the remaining solvent to yield a yellow-orange solid. The liquid was removed by decantation and the solid ([μ-1,1-*bis*(diphenylphosphino)­alkyl-η^6^ *-p-*cymenetriphenylphosphinochloridoruthenium­(II)]-chloridogold­(I) hexafluoroantimonate­(I)) was washed with cold diethyl ether (∼0.0 °C, 3 × 10.0 mL). For complex **5b**, [AuS­(Me)_2_Cl] (2 equiv) was used to synthesize [μ-1,1-*bis*(diphenylphosphinoethyl)­phenylphosphine-η^6^ *-p-*cymenetriphenylphosphino-chloridoruthenium­(II)]­dichloridogold­(I) hexafluoroantimonate­(I). The solid was stored *in vacuo* for 2 h to yield an orange powder of complexes **1b**, **2b**, **3b**, **4b**, and **5b** (79.8, 80.63, 78.56, 63.4, and 86.85%). Crystals suitable for X-ray crystallography were obtained by recrystallizing the product in DCM/Methanol [1:1]. A counterion exchange was done with complex **3b** by dissolving it and AgNO_3_ was in 10 cm^3^ DCM and stirred at ambient temperature for 24h. The AgSbF_6_ precipitate was filtered off, and discarded. The filtrated was allowed to dry, producing an orange powder that was washed with pentane to yield complex **3c**. **1b-** ^31^P­{^1^H} NMR (161.97 MHz, Chloroform-*d1*): δ 22.4 (d, *J* = 53.1 Hz), 20.3 (d, *J* = 18.9 Hz), 19.4 (dd, *J* = 52.1 Hz, 18.4 Hz). **2b-** ^31^P­{^1^H} NMR (161.97 MHz, Chloroform-*d1*): δ 30.1 (s), 23.3 (d, *J* = 9.8 Hz), 23.2 (s). **3b-** ^31^P­{^1^H} NMR (161.97 MHz, Chloroform-*d1*): δ 28.8 (s), 23.3 (d, *J* = 52.2 Hz), 18.5 (d, *J* = 52.3 Hz). **3c-** ^31^P­{^1^H} NMR (161.97 MHz, Chloroform-*d1*): δ 28.2 (s), 23.3 (m), 18.1 (dd, *J* = 135.9 Hz, 52.2 Hz). **4b-** ^31^P­{^1^H} NMR (161.97 MHz, Chloroform-*d1*): δ 31.4 (s), 22.7 (d, *J* = 52.6 Hz), 19.0 (d, *J* = 51.2 Hz). **5b-** ^31^P­{^1^H} NMR (161.97 MHz, Chloroform-*d1*): δ 34.1–33.1 (m), 31.8–29.7 (m), 23.9–23.5 (m), 22.9–22.7 (m).

### X-ray Diffraction

Single-crystal X-ray Diffraction data were collected on a Bruker D8 Quest Eco Chi Photon || CPAD diffractometer and a Bruker D8 Venture 4K Kappa Photon||| C28 diffractometer. The latter diffractometers are equipped with a graphite monochromated Mo X-ray source that has an X-ray wavelength of 0.71073 Å and collects φ- and ω-scans at 100 K. The data refinement was done using APEX III and data reduction was completed with SAINT-plus and XPREP.^68^ The SADABS software package was utilized for the absorption corrections.^69^ For structure solution and refinement, OLEX2,^70^ SHELXL-97,^71^ and WINGX^72^ software was used. The graphical representations of the molecular structures were created with DIAMOND.^73^ Non-hydrogen atoms were anisotropically refined and hydrogen atoms (aromatic, CH, CH_2_, and CH_3_) were placed in geometrically optimized positions with the HFIX 43, 13, 23, and 137 functions, respectively. The hydrogen atoms were constrained to ride on their parent atoms with U_iso_(H) = 1.2 U_eq_(C) for methine, methylene, and aromatic hydrogens and U_iso_(H) = 1.5 U_eq_(C) for methyl hydrogens. The constraints and optimized positions for the hydrogen atoms mean that the distances between the hydrogen atoms of methyl, methylene, methine, and aromatic groups and their parent atoms will be 0.980, 0.990, 1.000, and 0.950 Å, respectively. The checkCIF file for each crystal structure is in the Supporting Information A7. All tables containing the crystallographic data of **1a**, **1b**, **2b**, and **3b** can also be found in the Supporting Information A6.

### Hirshfeld

Hirshfeld surface plots and fingerprint plots of all the structures have been produced by Crystal Explorer 21 ^74^ giving a colored 2D projection of the Hirshfeld surfaces (see the Supporting Information A6). Each molecular crystal has a unique Hirshfeld surface which is obtained from the electron distribution around the atoms in the molecule that are calculated as the sum of the spherical atom electron densities.^75–77^ Additional insight into the intermolecular interactions in crystal structures is obtained and visualized by using 3D molecular surface contours and 2D fingerprint plots.

### Cytotoxicity Studies

#### Reagents

All reagents and solvents were purchased from Sigma-Aldrich (St. Louis, MO, USA), unless otherwise stated, and used without further purification. Dulbecco’s Modified Eagle Media (DMEM), Dulbecco’s Modified Eagle Media–Low glucose (DMEM-LG), and PBS with and without Ca^2+^ and Mg^2+^ were purchased from Cytiva (Marlborough, MA, USA). Foetal Bovine Serum (FBS) and penicillin/streptomycin were purchased from Biowest (Nuaillè, France). The African green monkey kidney cell line (Vero cells), the human colorectal adenocarcinoma cell line (Caco2), and human cervical cancer cell line (HeLa) cells were purchased from Cellonex, South Africa.

##### Cell Line Maintenance

HeLa cells were maintained in DMEM. The medium was supplemented with 10% fetal calf serum and 1% penicillin/streptomycin. Caco2 cells were maintained in 10 cm culture dishes in complete medium (DMEM-LG, 10% FBS, penicillin/streptomycin). The African green monkey kidney cell line, Vero cells, was used for cytotoxicity screening. The cells were maintained in 10 cm culture dishes in complete medium (DMEM supplemented with 10% FBS). All cells were incubated at 37.0 °C in a humidified atmosphere with 5% CO_2_.

##### Sample Preparation

Test samples were reconstituted in dimethyl sulfoxide (DMSO) to a stock concentration of 100 mM. Samples were sonicated if solubility was a problem and stored at 4 °C until required.

##### Cytotoxicity of HeLa Cells–SRB Assay

A monolayer of cells was trypsinized and suspended in 1 mL growth medium. The cell count was diluted to 0.5 × 10^5^ cells per mL, of which 0.1 mL was added to each well of a 96 well microplate. Plates were incubated for 1 h to allow cells to adhere. Varied concentrations (0.1 mL) of test compounds were added to each well. The plates were again incubated for 3 days at 37.0 °C with 5% CO_2_. Trichloroacetic acid (0.05 mL of 50%) was added to each well in order to fix cells and then stored overnight at 4.0 °C. The plates were washed under running tap water and dried at 50.0 °C for 2 h. The SRB dye (0.1 mL) was added to each well and kept in the dark for 30 min. Plates were washed with 1% acetic acid (0.1 mL × 4) in order to remove unbound stain, and air-dried overnight. The bound dye was solubilized by the addition of 10 mM tris buffer (0.1 mL) to each well. The plates were gently shaken for 1 h and the absorbance was measured at 510 nm. The absorbance measurements were used to determine growth inhibition of the test compounds as a percentage of the control group. One-way ANOVA with Dunnett’s post-test was performed using GraphPad Prism version 5.0.0 for Windows, GraphPad Software, San Diego California USA, www.graphpad.com. Data was fitted to a nonlinear regression of normalized response.

##### Cytotoxicity of Caco2 Cells–MTT Assay

Cells were seeded in 96 well plates at 4000 cells per well in 100 μL aliquots and left overnight to attach. The selected compounds that were tested, were prepared at the following concentrations for treatment: 0.01, 0.1, 1.0, 5.0, and 10.0 μM. Compound **1b** was tested at a slightly higher concentration range, 0.1, 1.0, 5.0, 10.0, and 50.0 μM since preliminary screening tests indicated that its IC_50_ concentration will be higher than the other compounds’ concentrations. Melphalan (30 μM) was used as a positive control. Treatments were prepared in complete medium, with DMSO vehicle controls included, and added to the cells. The cells were then incubated for 48 h, after which MTT treatments were aspirated. MTT (100 μL, 0.5 mg/mL) in complete medium was added to each well. The MTT treated cells were incubated for 3 h. Thereafter, 100 μL DMSO was added to each well. Absorbance was measured at 540 nm using a BioTek PowerWave XS spectrophotometer (Winooski, VT, USA). One-way ANOVA with Dunnett’s multiple comparisons test was performed using GraphPad Prism version 4.0.0 for Windows, GraphPad Software, San Diego California USA, www.graphpad.com, to determine the IC_50_ values of the selected compounds.

##### Screening Procedure of Vero Cells

Each compound was tested at 3 concentrations against proliferating (log-phase) and nonproliferating (confluent) Vero cells. For effect against proliferating cells: Cells were seeded into a 96 well microtiter plate at a density of 4000 cells per well using a volume of 100 μL in each well. For effect against nonproliferating cells: Cells were seeded into a 96 well microtiter plate at 15,000 cells per well using a volume of 100 μL in each well. The microtiter plates were incubated at 37.0 °C, 5% CO_2_, and 100% relative humidity for 24 h prior to the addition of test compounds to allow for cell attachment.

##### Compound Concentration

The Vero cells were treated with three concentrations of each compound: half of the IC_50_ concentration of the compound (Conc. 1), the IC_50_ concentration of the compound (Conc. 2), and double the IC_50_ concentration of the compound (Conc. 3). Table 1 summarizes the concentrations used for each compound. IC_50_ concentration refers to the IC_50_ values determined for the compounds against Caco2 cells. Melphalan (100 mM stock) was used as a positive control with an IC_50_ value of 30 μM. The cells were treated for 48 h at 37.0 °C, in an atmosphere with 5% CO_2_.

##### Staining Procedure

The dyes that were used in the staining procedure are Hoechst 33342 nuclear dye and propidium iodide (PI). The former was prepared as a 10 mL staining solution with a concentration of 5 μg/mL (2.5 μL Hoechst in 10 mL). The latter was prepared as a 100 μg/mL solution by diluting 20 μL PI in 10 mL PBS. The Hoechst dye (100 μL) staining solution was added to each well and the plates were incubated for 30 min, where after the PI solution (10 μL) was added to each well. The images of the plates were acquired immediately using the DAPI and Texas Red filters.

##### Data Acquisition and Analysis

The quantification of live and dead cells was performed using the ImageXpress Micro XLS Widefield Microscope (from Molecular Devices) using a 10× Plan Fluor objective and DAPI and Texas Red filter cubes. Nine image sites were acquired per well which is representative of roughly 70% of the surface area of the well. Acquired images were analyzed using the MetaXpress software and Multi-Wavelength Cell Scoring Application Module, from Molecular Devices, California, USA. Acquired data was transferred to an Excel spreadsheet where data was analyzed and processed.

## Supplementary Material



## Notes

NMR spectra and ^1^H data, FT-IR data, Crystallography data, Hirshfeld data, CheckCIFs, and ESI-MS spectra are included in the Supporting Information (PDF)
