# Supplementary material for: High Cytotoxicity of Ruthenium(II) and Gold(I) Bimetallic Complexes and Its Precursors
Source: Inorg Chem. 2026 Jan 6;65(2):1463–76. doi: 10.1021/acs.inorgchem.5c04993 (PMC12820967; doi:10.1021/acs.inorgchem.5c04993)
Supplement: Supplementary file 1 [file ic5c04993_si_001.pdf]

Supporting Information

High Cytotoxicity of Ruthenium(II) and Gold(I) bimetallic complexes and its precursors.

Janine Blignaut[a], Hendrik G. Visser[a], Eleanor Fourie[a], and Marietjie Schutte-Smith[a]*

[a] University of the Free State, Department of Chemistry, Nelson Manda Drive, Bloemfontein, South Africa, 9301.

* Email: schuttem@ufs.ac.za

Table of Contents

A1 - Synthesis 2

A2 -Summary of $^{31}P\{H\}$ NMR data 13

A3 – ^{1}H-NMR Spectra 6

A4 - $^{31}P\{H\}$ NMR Spectra 13

A5 - ESI-MS Spectra 21

A6 - X-Ray Diffraction Discussion 27

A7 - X-Ray Diffraction and Hirshfeld data 30

A1 - Synthesis

η⁶-*p*-Cymenedichloridotriphenylphosphinoruthenium(II) (Ru(II)-PPh_3)- Ru(II)-dimer complex (1 eq) was dissolved in hexane (20 cm^3) and refluxed with triphenylphosphine (3 eq) for 4 h. The precipitate was filtered off and washed with hexane (97.5 %) ^{1}H NMR (400.13 MHz, Chloroform-d1): δ): δ 7.85-7.80 (m, 5H), 7.39-7.33 (m, 10H), 5.19 (d, *J* = 6.16 Hz, 2H), 4.99 (dd, *J* = 6.20 Hz, 1.16 Hz, 2H), 2.88-2.81 (m, 1H), 1.86 (s, 3H), 1.10 (d, *J* = 6.96 Hz, 6H). ^{31}P{H} NMR (161.97 MHz, Chloroform-*d1*): δ 24.17 (s).) IR (ATR, cm^{-1}): ν_{PPh3} = 741.74, 691.16. UV/Vis (ε, λ_{max} = 265 nm): 2317.67 M^{-1} cm^{-1}.

η⁶-*p*-Cymeneacetonitrilechloridotriphenylphosphineruthenium(II) hexafluoroantimonate(I) (Ru(II)-acetonitrile) - A reaction mixture of Ru(II)-PPh_3 (1 eq) and $AgSbF_6$ (1 eq) in $CNCH_3$ (70.0 cm^3) was refluxed (~82.0 °C) for 10 min. The solvent was removed until ~5.0 cm^3 of the solvent was left. The product was precipitated out as an oil by addition of pentane. The supernatant liquid was removed by decantation. The oil was then sonicated twice in pentane and then diethyl ether.(94.2 %) ^{1}H NMR (400.13 MHz, Chloroform-*d1*): δ 7.61-7.47 (m, 15H), 6.03 (dd, *J* = 6.24 Hz, 1.28 Hz, 1H), 5.93 (d, *J* = 6.08 Hz, 1H), 5.38 (d, *J* = 5.96 Hz, 1H), 4.65 (d, *J* = 6.24 Hz, 1H), 3.05-2.98 (m, 1H), 1.91 (d, *J* = 1.44 Hz, 3H), 1.40-1.34 (m, 6H). ^{31}P{H} NMR (161.97 MHz, Chloroform-*d1*): δ 35.40 (s)). IR (ATR, cm^{-1}): ν_{CN} = 1481.86, 1435.01. UV/Vis (ε, λ_{max} = 265 nm): 2975.82 M^{-1} cm^{-1}.

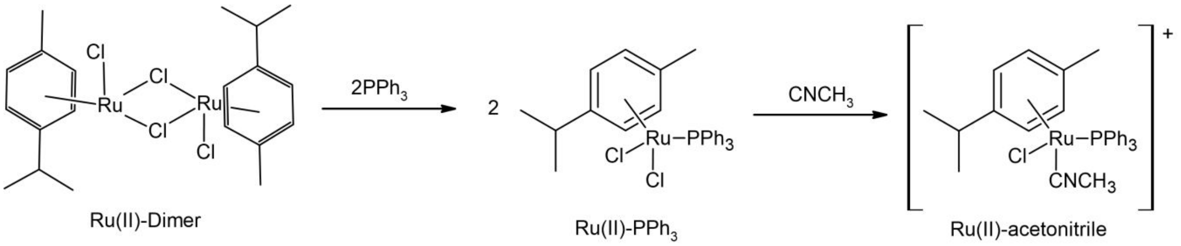

Figure S1. The reaction scheme of the Ru(II) precursors

[μ-1,1-Bis(diphenylphosphino)alkyl-η⁶-*p*-cymenetriphenylphosphinochloridoruthenium(II)] hexafluoroantimonate(I) (Ru(II)-$PC_{(n)}P$) complexes.

1a ([μ-1,1-*bis*(diphenylphosphino)methan-*η*⁶-*p*-cymenetriphenylphosphinochlorido-ruthenium(II)] hexafluoroantimonate(I))- ^{1}H NMR (400.13 MHz, Chloroform-*d1*): δ 7.41-6.94 (m, 35H), 5.88 (t, 1H), 5.62 (d, *J* = 5.92 Hz, 1H), 5.22 (t, 1H), 4.94 (d, *J* =

6.20 Hz, 1H), 3.66 (dd, J = 14.73 Hz, 6.28 Hz, 1H), 2.80-2.73 (m, 1H), 1.27 (dd, J = 22.97 Hz, 6.84 Hz, 6H), 1.11-1.07 (m, 1H), 0.93 (s, 3H). $^{31}P\{^1H\}$ NMR (161.97 MHz, Chloroform-*d1*): δ 23.0 (d, J = 13.5 Hz), 22.9 (d, J = 27.8 Hz), -29.3 (dd, J = 27.9, 13.7 Hz). IR (ATR, cm^{-1}): v_{PC} = 1086, 1014. UV/Vis (ε, λ_{max} = 350 nm): 7427.67 M^{-1} cm^{-1}. Anal. Calcd for $C_{54}H_{53}P_3RuCl_3Sb_1F_6$: C, 52.38; H, 4.32. Found: C, 52.43; H, 4.36. ESI-MS. Calcd for [C27H26NORu]+ ESI-MS (positive ion mode): *m/z* 918.28 [M – SbF_6^-]$^+$, calc. 917.46.

2a ([µ-1,1-*bis*(diphenylphosphino)ethan-η^6-*p*-cymenetriphenylphosphinochlorido-ruthenium(II)] hexafluoroantimonate(I))- ^1H NMR (400.13 MHz, Chloroform-*d1*): δ 7.39-7.23 (m, 35H), 6.03 (s, 1H), 5.64 (d, J = 6.76 Hz, 1H), 5.19 (s, 1H), 4.83 (d, J = 5.68 Hz, 1H), 2.78-2.69 (m, 1H), 2.65-2.62 (m, 1H), 2.17-2.08 (m, 1H), 1.45-1.42 (m, 1H), 1.26 (dd, J = 23.97 Hz, 6.88 Hz, 6H), 1.01-0.97 (m, 1H), 0.85 (s, 3H). $^{31}P\{^1H\}$ NMR (161.97 MHz, Chloroform-*d1*): δ 23.7 (d, J = 30.4 Hz), 23.4 (s), -14.7 (dd. J = 29.3, 3.9 Hz). IR (ATR, cm^{-1}): v_{PC} = 1087, 1025. UV/Vis (ε, λ_{max} = 345 nm): 3927.37 M^{-1} cm^{-1}. Anal. Calcd for $C_{54}H_{53}P_3RuCl_1Sb_1F_6$: C, 55.57; H, 4.58. Found: C, 55.59; H, 4.61. ESI-MS (positive ion mode): *m/z* 932.28 [M – SbF_6^-]$^+$, calc. 931.49.

3a ([µ-1,1-*bis*(diphenylphosphino)propan-η^6-*p*-cymenetriphenylphosphinochlorido-ruthenium(II)] hexafluoroantimonate(I))- ^1H NMR (400.13 MHz, Chloroform-*d1*): δ 7.56-7.07 (m, 35H), 5.97-5.95 (m, 1H), 5.65 (d, J = 6.20 Hz, 1H), 5.23-5.20 (m, 1H), 4.88 (d, J = 6.04 Hz, 1H), 2.77-2.70 (m, 1H), 2.64-2.55 (m, 1H), 1.60-1.53 (s, 3H) 1.46-1.39 (m, 1H), 1.25 (dd, J = 17.57 Hz, 6.96 Hz, 6H), 0.83 (m, 4H). $^{31}P\{^1H\}$ NMR (161.97 MHz, Chloroform-*d1*): δ 23.4 (d, J = 52.2 Hz), 18.7 (d, J = 52.4 Hz), -19.3 (s). IR (ATR, cm^{-1}): v_{PC} = 1086, 1014. UV/Vis (ε, λ_{max} = 345 nm): 4343.31 M^{-1} cm^{-1}. Anal. Calcd for $C_{55}H_{55}P_3RuCl_1Sb_1F_6$: C, 55.92; H, 4.69. Found: C, 56.02; H, 4.73. ESI-MS (positive ion mode): *m/z* 946.29 [M – SbF_6^-]$^+$, calc. 945.52.

4a ([µ-1,1-*bis*(diphenylphosphino)butan-η^6-*p*-cymenetriphenylphosphinochlorido-ruthenium(II)] hexafluoroantimonate(I))- ^1H NMR (400.13 MHz, Chloroform-*d1*): δ 7.35-7.14 (m, 35H), 6.00 (s, 1H), 5.66 (s, 1H), 5.20 (s, 1H), 4.87 (s, 1H), 2.74-2.71 (m, 1H), 2.45 (s, 1H), 2.01 (s, 2H), 1.28-1.22 (m, 6H), 0.84-0.83 (m, 6H), 0.37 (s, 2H). $^{31}P\{^1H\}$ NMR (161.97 MHz, Chloroform-*d1*): δ 23.0 (d, J = 52.7 Hz), 18.8 (d, J = 52.4 Hz), -16.4 (s). IR (ATR, cm^{-1}): v_{PC} = 1088, 1015. UV/Vis (ε, λ_{max} = 350 nm): 3875.08 M^{-1} cm^{-1}. Anal. Calcd for $C_{56}H_{57}P_3RuCl_1Sb_1F_6$: C, 56.27; H, 4.81. Found: C, 56.64; H, 4.83. ESI-MS (positive ion mode): *m/z* 960.29 [M – SbF_6^-]$^+$, calc. 959.55.

5a ([μ-1,1-*bis*(diphenylphosphinoethyl)phenylphosphine-η^6-*p*-cymenetriphenylphosphinochloridoruthenium(II)] hexafluoroantimonate(I))- ^{1}H NMR (400.13 MHz, Chloroform-*d1*): δ 7.65-7.01 (m, 40H), 6.00 (s, 1H), 5.66 (d, *J* = 15.68 Hz, 1H), 5.19 (s, 1H), 4.81 (d, *J* = 11.90 Hz, 1H), 2.79-2.67 (m, 1H), 1.57 (s, 3H), 1.26 (s, 6H), 0.88-0.83 (m, 9H). ^{31}P{^{1}H} NMR (161.97 MHz, Chloroform-*d1*): δ 24.2 - 18.7 (m), -12.7 - -16.2 (m). IR (ATR, cm^{-1}): ν_{PC} = 1088, 1016. UV/Vis (ε, λ_{max} = 347 nm): 5465.66 M^{-1} cm^{-1}. Anal. Calcd for $C_{62}H_{62}P_4RuCl_1Sb_1F_6$: C, 57.14; H, 4.79. Found: C, 57.11; H, 4.81. ESI-MS (positive ion mode): *m/z* 1068.28 [M – SbF_6^-]$^+$, calc. 1067.63.

[μ-1,1-Bis(diphenylphosphino)alkyl-η^6-*p*-cymenetriphenylphosphinochloridoruthenium(II)]chloridogold(I) hexafluoroantimonate(I) (Ru(II)-PC$_{(n)}$P-Au(I)) complexes.

1b ([μ-1,1-*bis*(diphenylphosphino)methan-η^6-*p*-cymenetriphenylphosphinochlorido-ruthenium(II)]chloridogold(I) hexafluoroantimonate(I))- ^{1}H NMR (400.13 MHz, Chloroform-*d1*): δ 7.46-7.09 (m, 35H), 5.73 (t, 1H), 5.65 (d, *J* = 6.00 Hz, 1H), 5.25 (t, 1H), 4.87 (d, *J* = 6.36 Hz, 1H), 2.74-2.66 (m, 1H), 2.12 (s, 2H), 1.22 (dd, *J* = 23.89 Hz, 6.94 Hz, 6H), 0.90 (s, 3H). ^{31}P{^{1}H} NMR (161.97 MHz, Chloroform-*d1*): δ 22.4 (d, *J* = 53.1 Hz), 20.3 (d, *J* = 18.9Hz), 19.4 (dd, *J* = 52.1 Hz, 18.4 Hz). IR (ATR, cm^{-1}): ν_{PC} = 740, 691, 652. UV/Vis (ε, λ_{max} = 357 nm): 3314.45 M^{-1} cm^{-1}. Anal. Calcd for $C_{54}H_{53}P_3RuAuCl_4Sb_1F_6$: C, 44.11; H, 3.63. Found: C, 44.19; H, 3.61. ESI-MS (positive ion mode): *m/z* 1151.17 [M – SbF_6^-]$^+$, calc. 1149.88.

2b ([μ-1,1-*bis*(diphenylphosphino)ethan-η^6-*p*-cymenetriphenylphosphinochlorido-ruthenium(II)]chloridogold(I) hexafluoroantimonate(I))- ^{1}H NMR (400.13 MHz, Chloroform-*d1*): δ 7.58-7.16 (m, 35H), 6.16 (s, 1H), 5.66 (s, 1H), 5.21 (s, 1H), 4.86 (s, 1H), 2.76-2.71 (m, 2H), 2.45 (s, 1H), 1.50-1.41 (m, 1H), 1.27 (dd, *J* = 21.49 Hz, 6.84 Hz, 6H), 0.87 (s, 3H), 0.81-0.75 (m, 1H).^{31}P{^{1}H} NMR (161.97 MHz, Chloroform-*d1*): δ 30.1 (s), 23.3 (d, *J* = 9.8 Hz), 23.2 (s). IR (ATR, cm^{-1}): ν_{PC} = 692, 653. UV/Vis (ε, λ_{max} = 357 nm): 3238.69 M^{-1} cm^{-1}. Anal. Calcd for $C_{54}H_{53}P_3RuAuCl_2Sb_1F_6$: C, 46.34; H, 3.82. Found: C, 46.32; H, 3.84. ESI-MS (positive ion mode): *m/z* 1164.19 [M – SbF_6^-]$^+$, calc. 1163.91.

3b ([μ-1,1-*bis*(diphenylphosphino)propan-η^6-*p*-cymenetriphenylphosphinochlorido-ruthenium(II)]-chloridogold(I) hexafluoroantimonate(I))- ^{1}H NMR (400.13 MHz, Chloroform-*d1*): δ 7.66-7.27 (m, 35H), 5.99-5.96 (m, 1H), 5.37 (d, *J* = 6.23 Hz, 1H), 5.21-5.19 (m, 1H), 5.10 (d, *J* = 5.82 Hz, 1H), 2.96-2.79 (m, 1H), 2.72-2.65 (m, 1H),

2.61-2.56 (m, 1H) 2.03 (s, 1H), 1.84 (s,1H), 1.25 (m, 6H), 1.03 (s, 2H), 0.88 (s, 3H). $^{31}P\{^1H\}$ NMR (161.97 MHz, Chloroform-*d1*): δ 28.8 (s), 23.3 (d, *J* = 52.2 Hz), 18.5 (d, *J* = 52.3 Hz). **IR** (ATR, cm^{-1}): ν_{PC} = 743, 692, 652. UV/Vis (ε, λ_{max} = 359 nm): 27 162.59 M^{-1} cm^{-1}. Anal. Calcd for $C_{55}H_{55}P_3RuAuCl_2Sb_1F_6$: C, 46.73; H, 3.92. Found: C, 46.61; H, 3.94. ESI-MS (positive ion mode): *m/z* 1178.20 $[M – SbF_6^-]^+$, calc. 1177.94.

3c ([μ-1,1-*bis*(diphenylphosphino)propan-*η*^{6}-*p*-cymenetriphenylphosphinochlorido-ruthenium(II)]chloridogold(I) nitrate)- ^1H NMR (400.13 MHz, Chloroform-*d1*): 7.53-7.31 (m, 35H), 5.55 (d, *J*=5.6 Hz, 1H), 5.35-5.33 (m, 1H), 5.18 (d, *J*=23.7, 1H), 4.96 (dd, *J*=24.8, 4.7 Hz 1H), 3.17-3.14 (m, 4H), 2.71-2.68 (m, 2H), 2.54-2.49 (m. 1H), 2.17 (s, 3H), 1.37-1.34 (m, 6H). $^{31}P\{^1H\}$ NMR (161.97 MHz, Chloroform-*d1*): δ 28.2 (s), 23.3 (m), 18.1 (dd, *J* = 135.9 Hz, 52.2 Hz). **IR** (ATR, cm^{-1}): ν_{PC} = 745, 693, 654. UV/Vis (ε, λ_{max} = 359 nm): 2524.19 M^{-1} cm^{-1}. Anal. Calcd for $C_{55}H_{55}P_3RuAuCl_2NO_3$: C, 53.28; H, 4.47; N, 1.13. Found: C, 53.34; H, 4.50; N, 1.14. ESI-MS (positive ion mode): *m/z* 1178.21 $[M – SbF_6^-]^+$, calc. 177.94.

4b ([μ-1,1-*bis*(diphenylphosphino)butan-*η*^{6}-*p*-cymenetriphenylphosphinochlorido-ruthenium(II)]chloridogold(I) hexafluoroantimonate(I))- ^1H NMR (400.13 MHz, Chloroform-*d1*): δ 7.50-7.12 (m, 35H), 5.89 (s, 1H), 5.60 (d, *J* = 5.2 Hz, 1H), 5.19 (s, 1H), 4.93 (d, *J* = 4.98 Hz, 1H), 2.73-2.68 (m, 1H), 2.36-2.31 (m, 1H), 2.06 (s, 2H), 1.86 (s, 2H), 1.67 (s, 1H), 1.25-1.21 (m, 6H), 1.11 (s, 2H), 0.87 (s, 3H). $^{31}P\{^1H\}$ NMR (161.97 MHz, Chloroform-*d1*): δ 31.4 (s), 22.7 (d, *J*= 52.6 Hz), 19.0 (d, *J*= 51.2 Hz). **IR** (ATR, cm^{-1}): ν_{PC} = 797, 742, 692, 653. UV/Vis (ε, λ_{max} = 356 nm): 2682.92 M^{-1} cm^{-1}. Anal. Calcd for $C_{56}H_{57}P_3RuAuCl_2Sb_1F_6$: C, 47.11; H, 4.02. Found: C, 47.15; H, 4.03. ESI-MS (positive ion mode): *m/z* 1193.24 $[M – SbF_6^-]^+$, calc. 1191.97.

5b ([μ-1,1-*bis*(diphenylphosphinoethyl)phenylphosphine-*η*^{6}-*p*-cymenetriphenyl-phosphinochloridoruthenium(II)]dichloridogold(I) hexafluoroantimonate(I))- ^1H NMR (400.13 MHz, Chloroform-*d1*): δ 7.69-6.91 (m, 40H), 5.22-4.92 (m, 4H), 2.75-2.74 (m, 1H), 2.43-2.31 (m, 4H), 2.11 (s, 1H) 1.79-1.73 (m, 2H), 1.25 (s, 6H), 1.10-1.02 (m, 1H), 0.91-0.080 (m, 3H). $^{31}P\{^1H\}$ NMR (161.97 MHz, Chloroform-*d1*): δ 34.1-33.1 (m), 31.8-29.7 (m), 23.9-23.5 (m), 22.9-22.7 (m). IR (ATR, cm^{-1}): ν_{PC} = 802, 745, 726, 693, 654. UV/Vis (ε, λ_{max} = 358 nm): 2651.42 M^{-1} cm^{-1}. Anal. Calcd for $C_{62}H_{62}P_4RuAu_2Cl_3Sb_1F_6$: C, 42.12; H, 3.53. Found: C, 42.16; H, 3.52. ESI-MS (positive ion mode): *m/z* 1533.7 $[M – SbF_6^-]^+$, calc. 1532.47.

A2 – ^{1}H-NMR Spectra

Starting Materials

Figure S2. ^{1}H NMR Spectrum of Ru(II)-PPh3

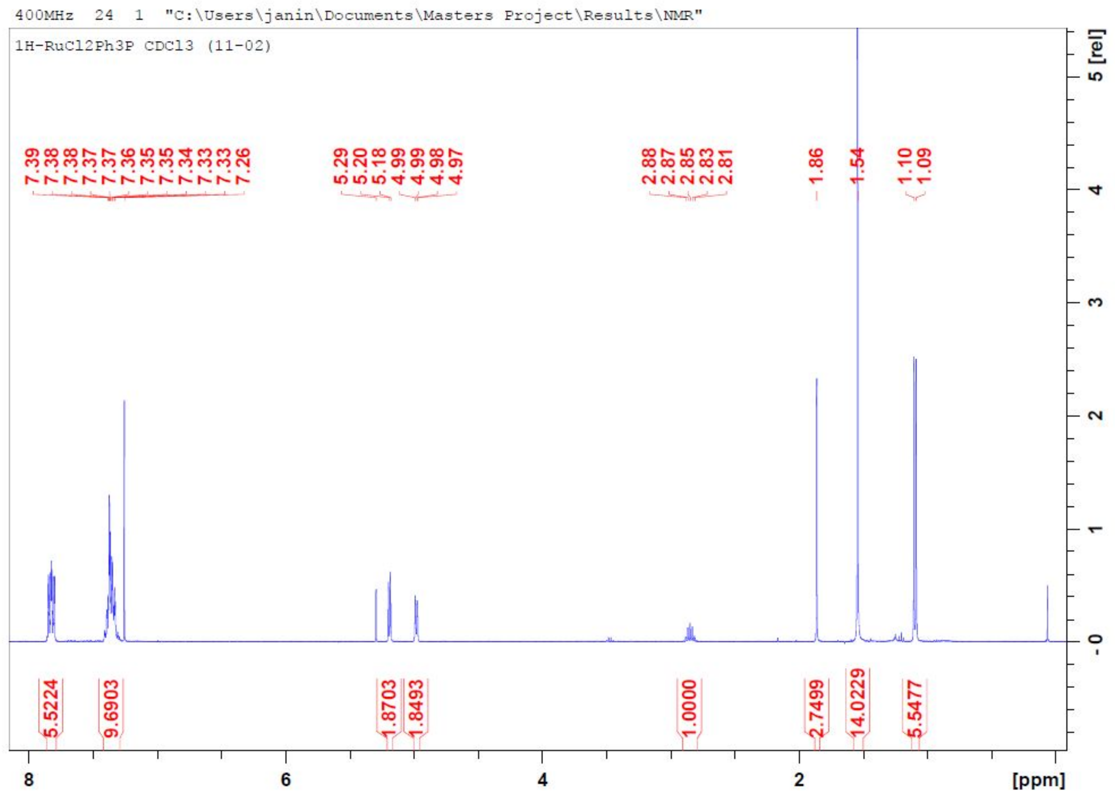

Figure S3. ^{1}H NMR Spectrum of Ru(II)-acetonitrile

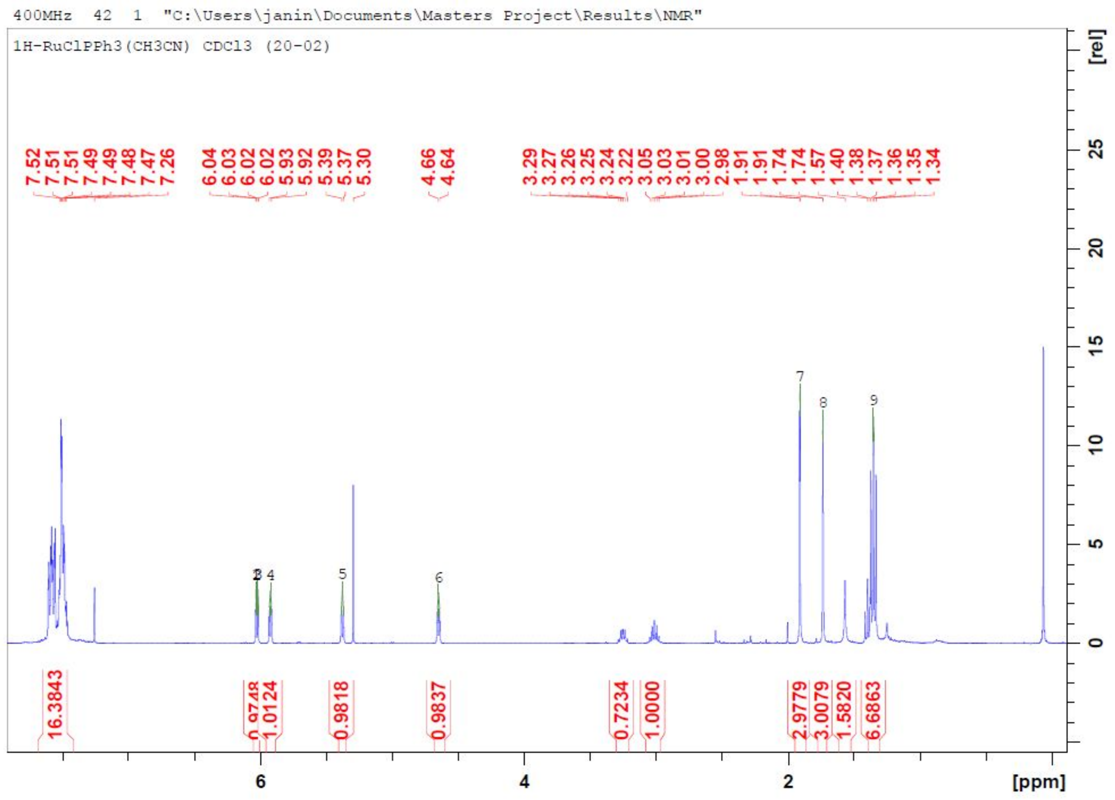

Ru(I)-PC$_{(n)}$P Complexes

Figure S4. ^{1}H NMR Spectrum of Ru(II)-PC$_{(1)}$P·SbF_6

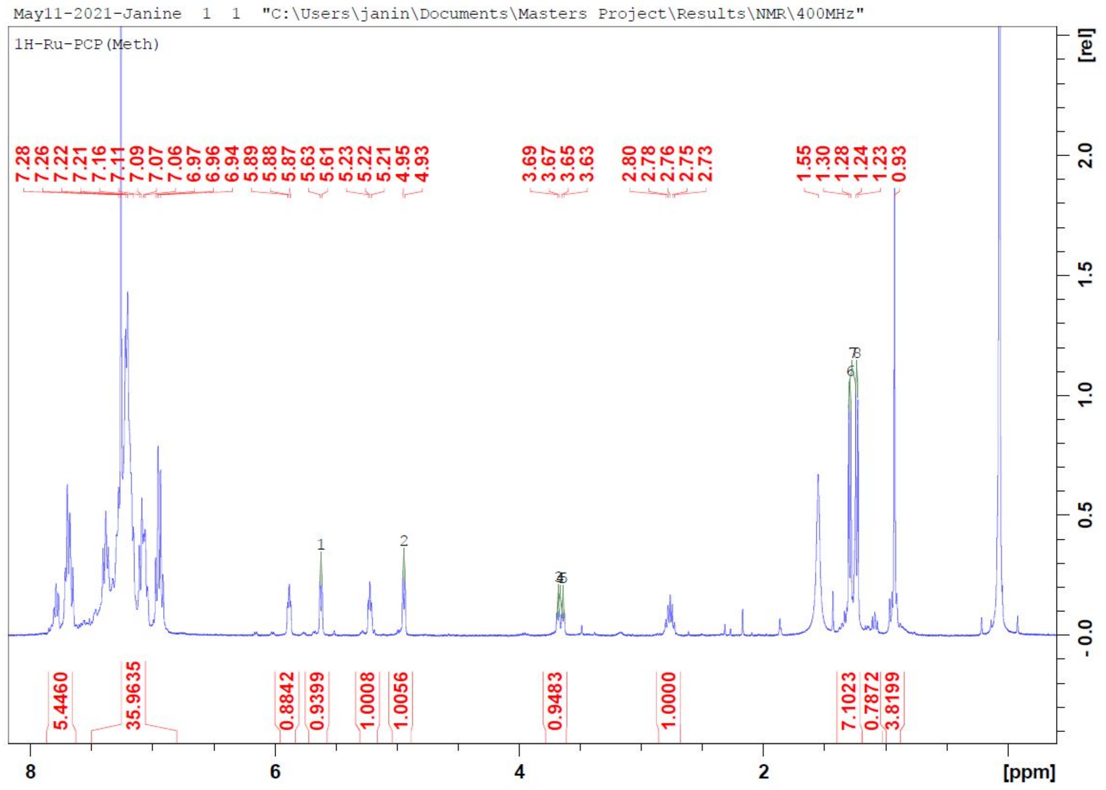

Figure S5. ^{1}H NMR Spectrum of Ru(II)-PC$_{(2)}$P·SbF_6

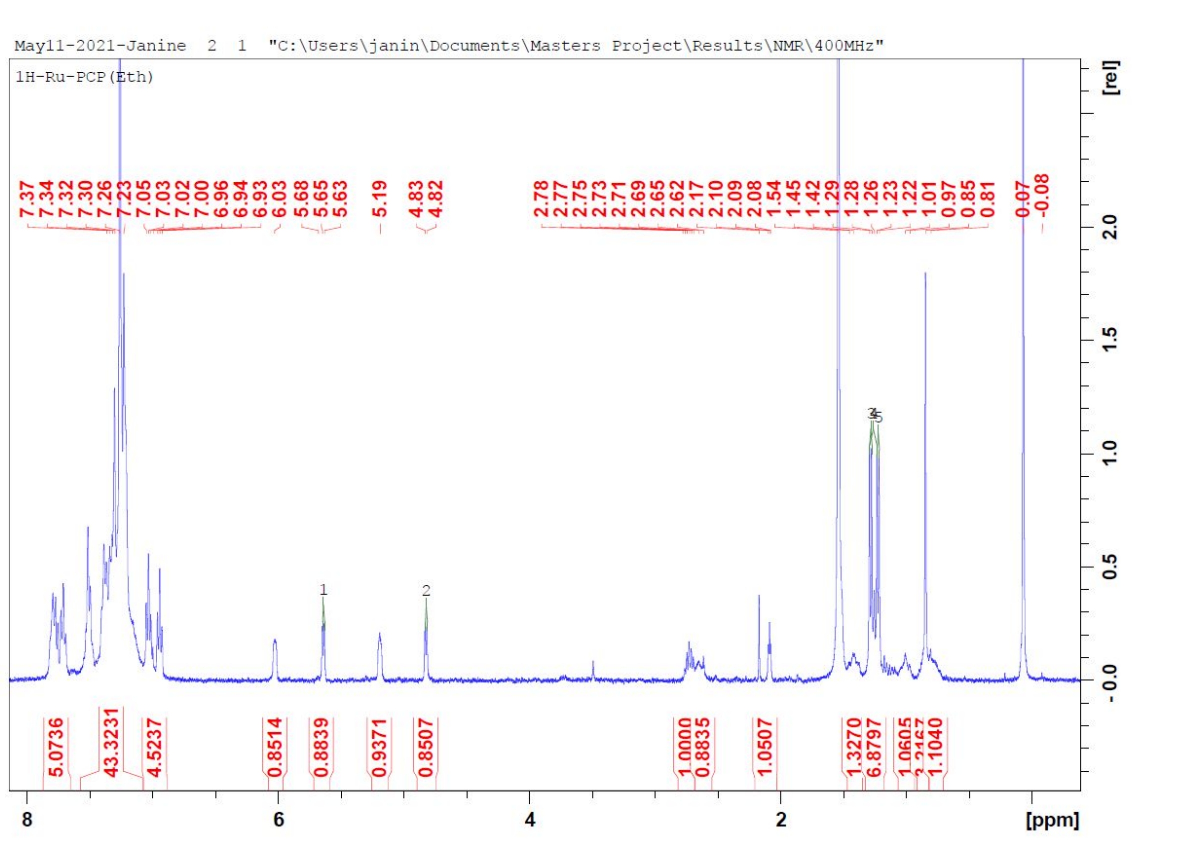

Figure S6. ^{1}H NMR Spectrum of Ru(II)-PC$_{(3)}$P·SbF$_6$

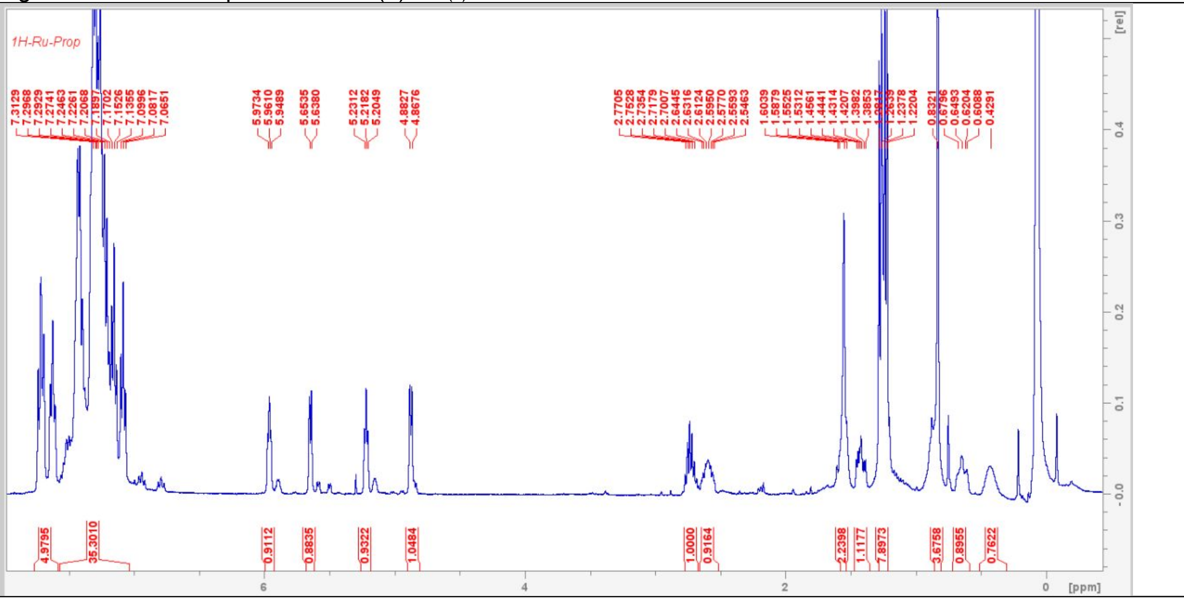

Figure S7. ^{1}H NMR Spectrum of Ru(II)-PC$_{(4)}$P·SbF$_6$

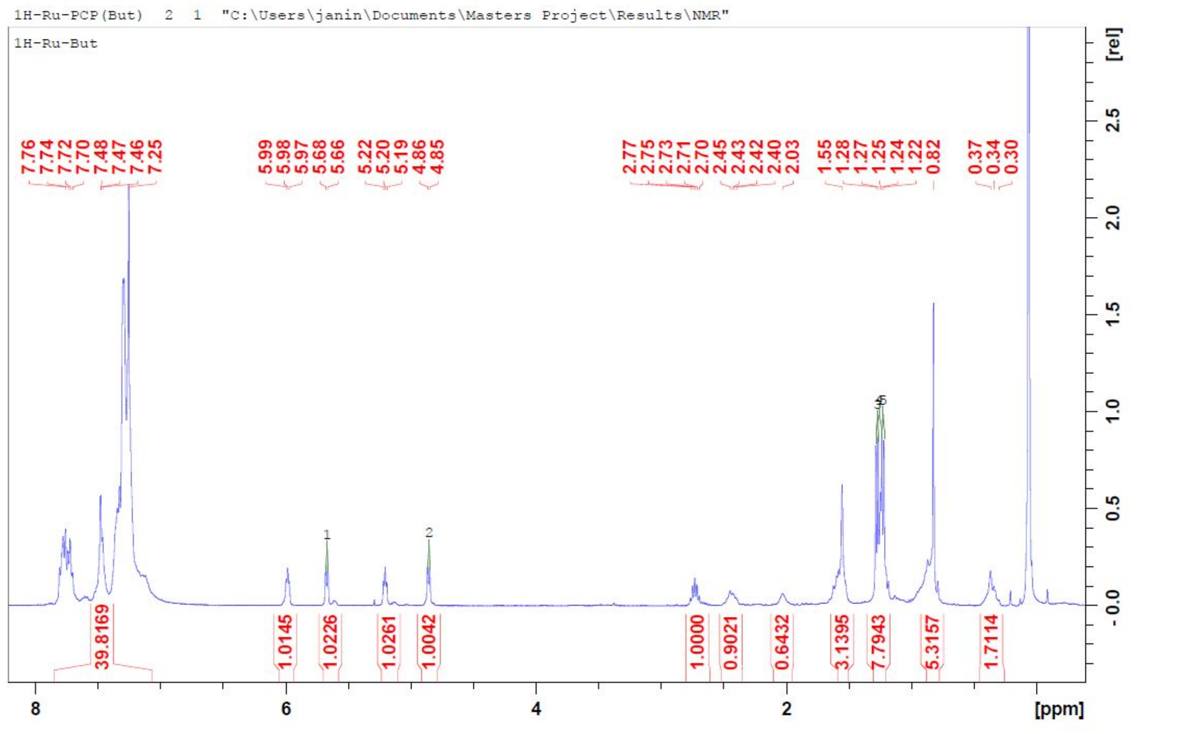

Figure S8. ^{1}H NMR Spectrum of Ru(II)-$PC_{(2)}PC_{(2)}P \cdot SbF_6$

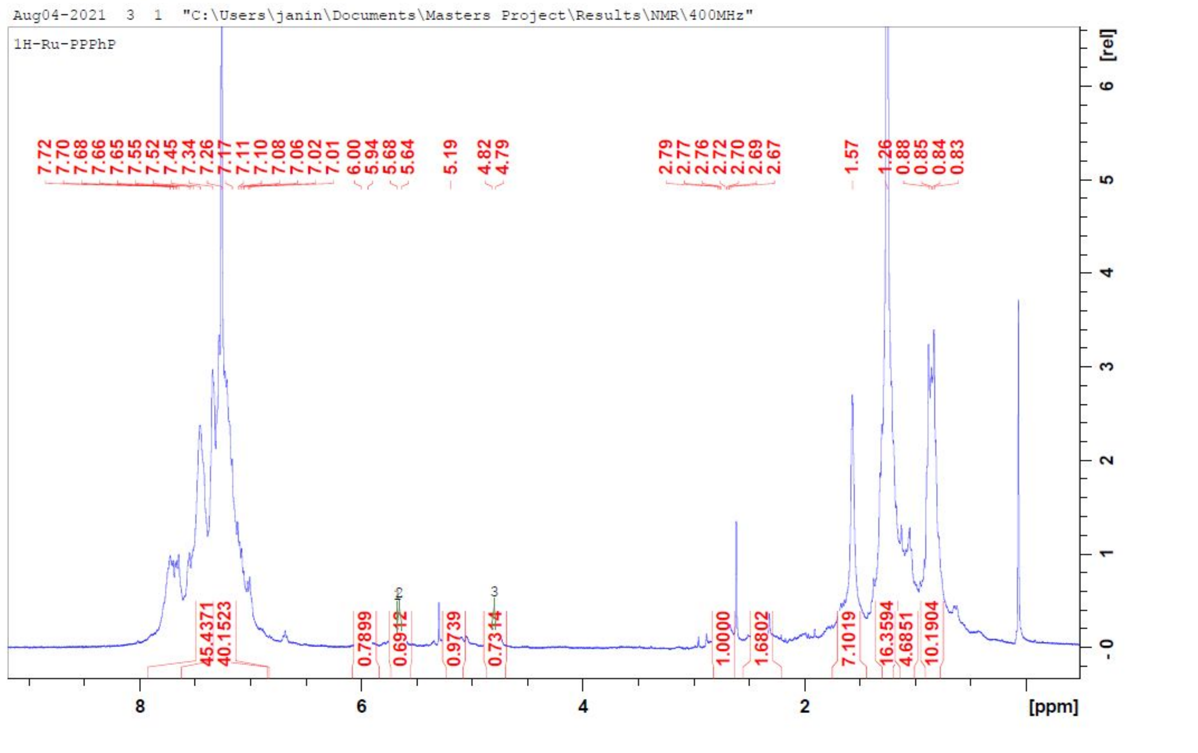

Ru(I)-$PC_{(n)}P$-Au(I) Complexes

Figure S9. ^{1}H NMR Spectrum of Ru(II)-$PC_{(1)}P$-Au(I)$\cdot SbF_6$

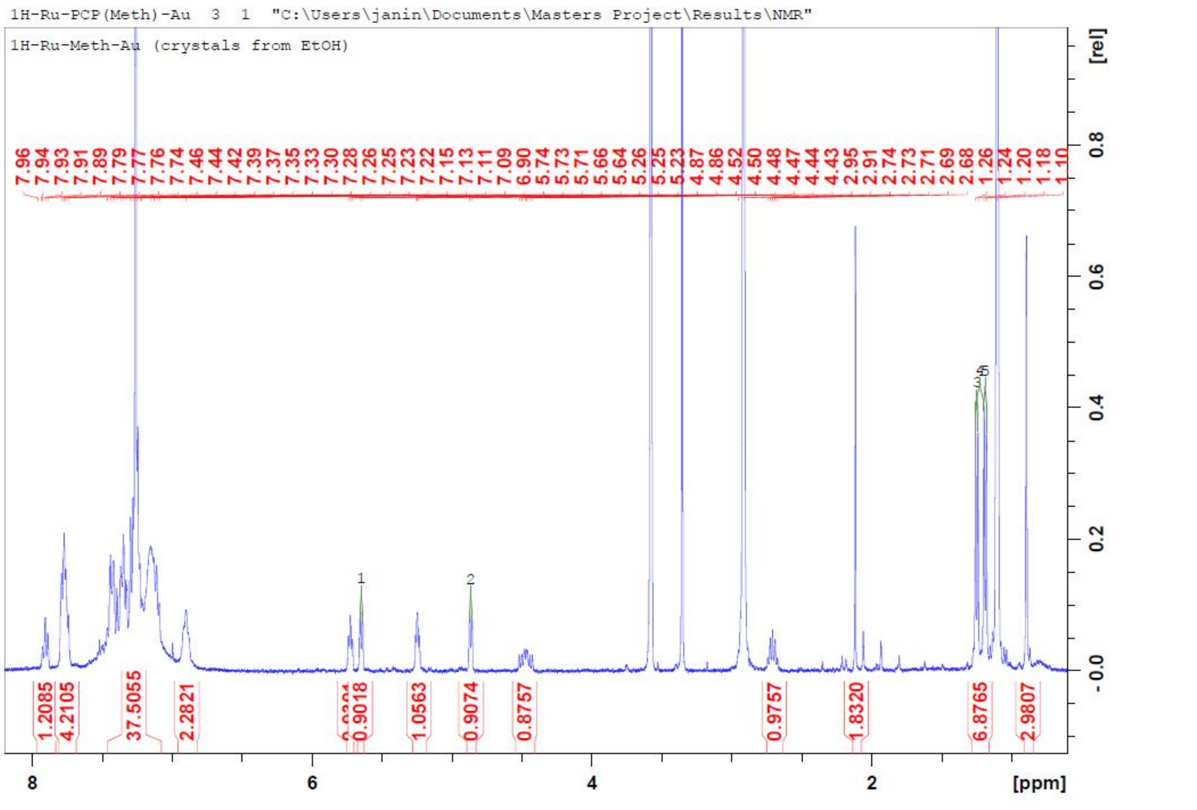

Figure S10. ^{1}H NMR Spectrum of Ru(II)-$PC_{(2)}P$-Au(I)·SbF_6

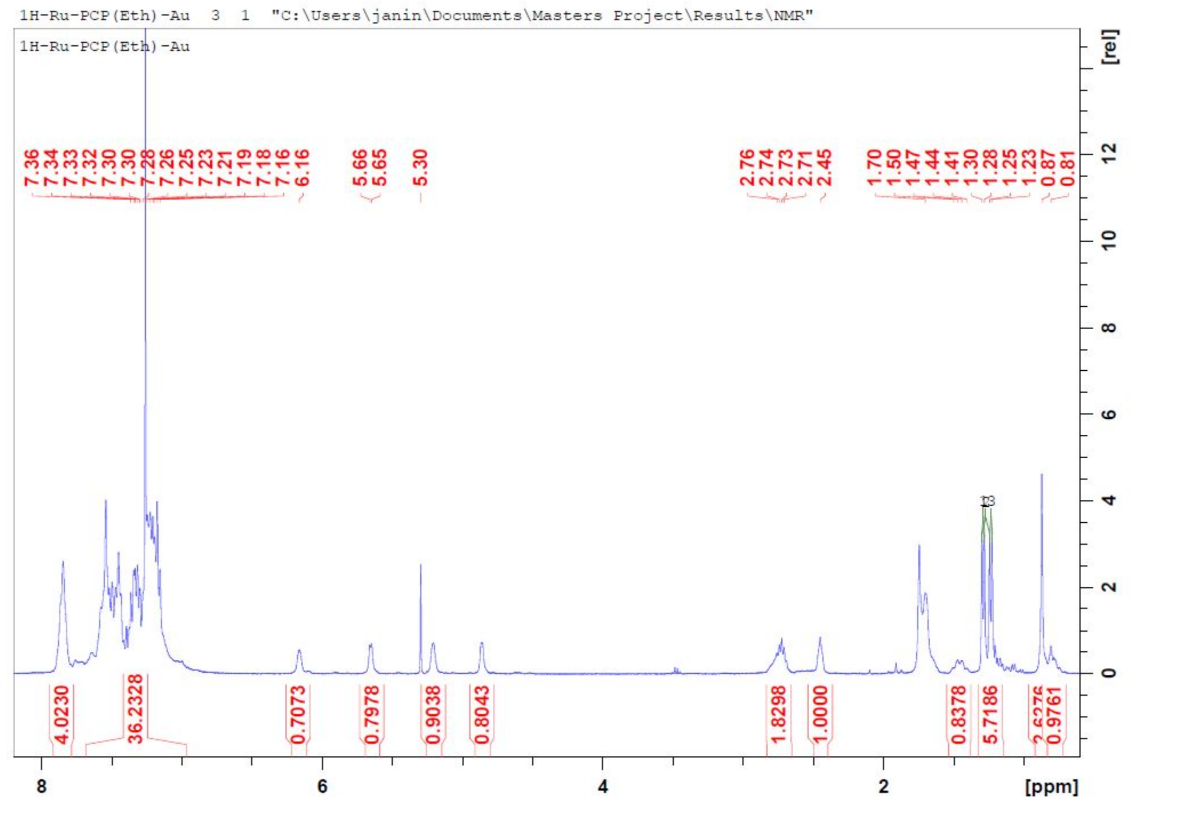

Figure S11. ^{1}H NMR Spectrum of Ru(II)-$PC_{(3)}P$-Au(I)·SbF_6

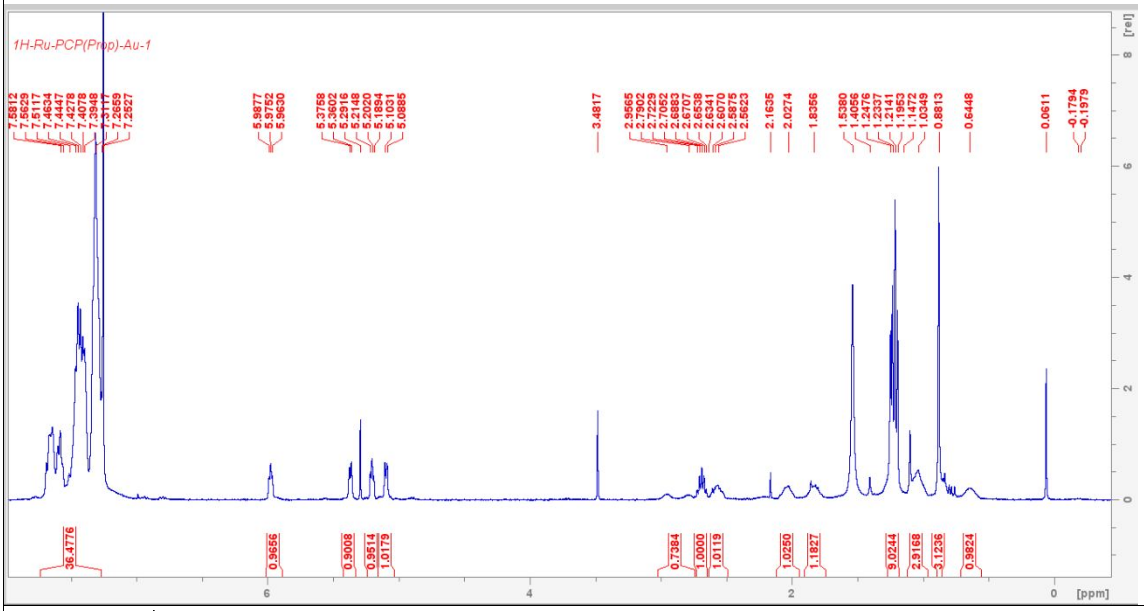
Figure S12. ^{1}H NMR Spectrum of Ru(II)-PC$_{(3)}$P-Au(I)·NO_3

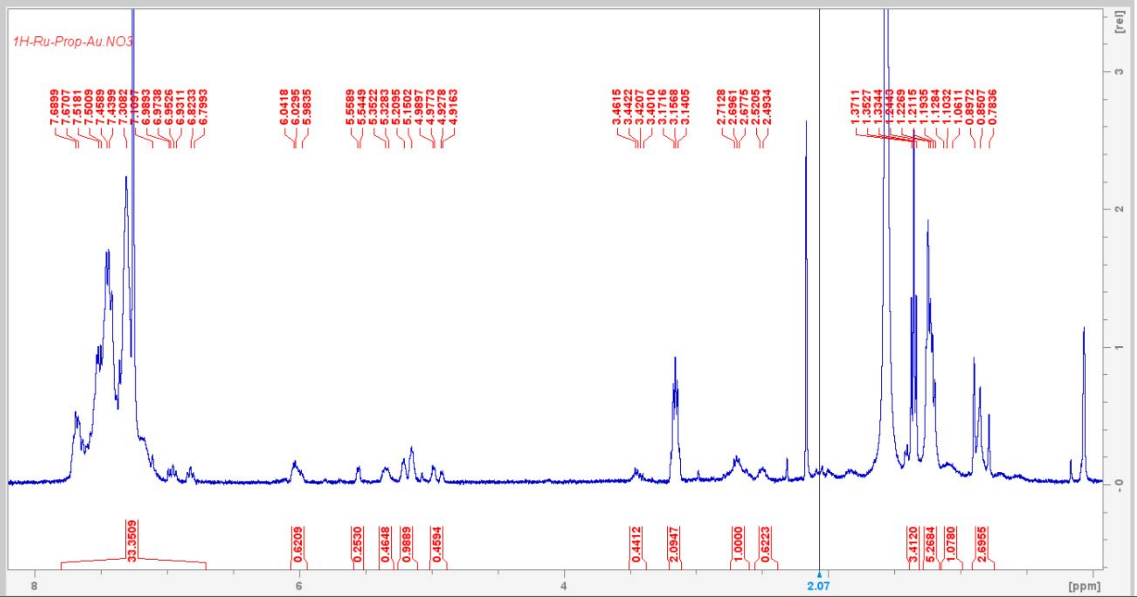
Figure S13. ^{1}H NMR Spectrum of Ru(II)-PC$_{(4)}$P-Au(I)·SbF_6

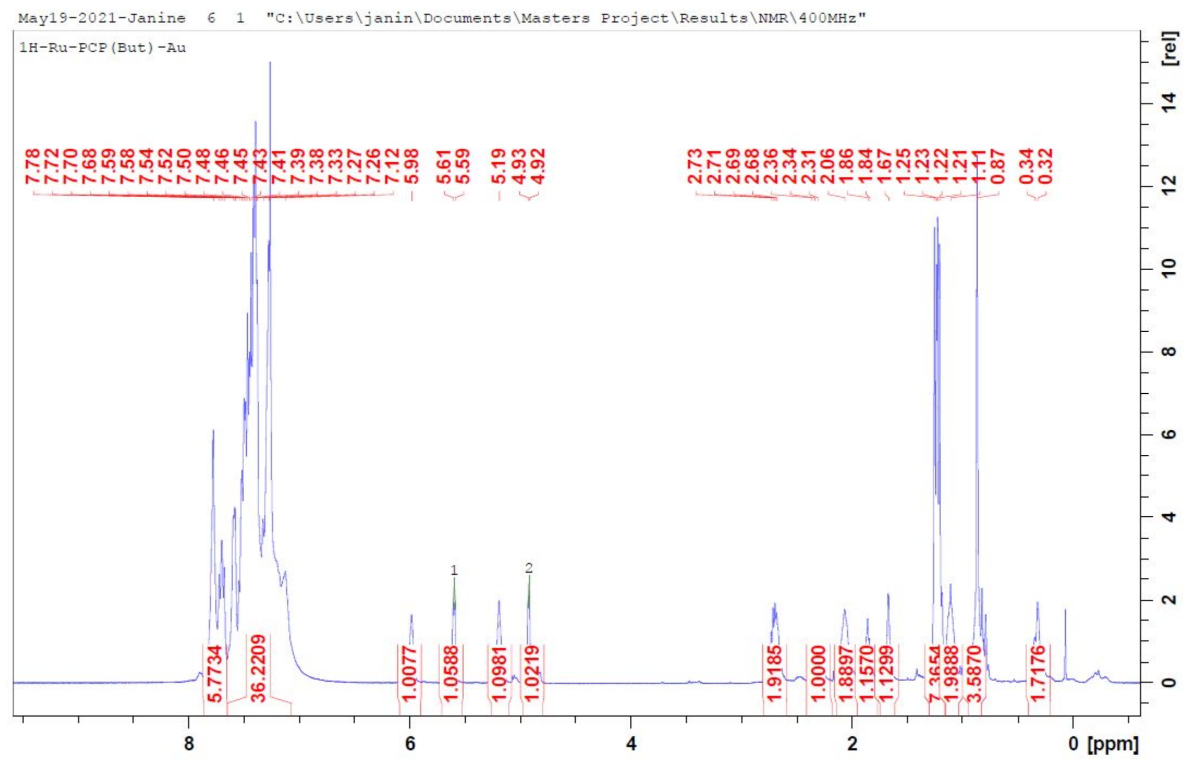

Figure S14. ^{1}H NMR Spectrum of Ru(II)-$PC_{(2)}PC_{(2)}P$-Au(I)·SbF_6

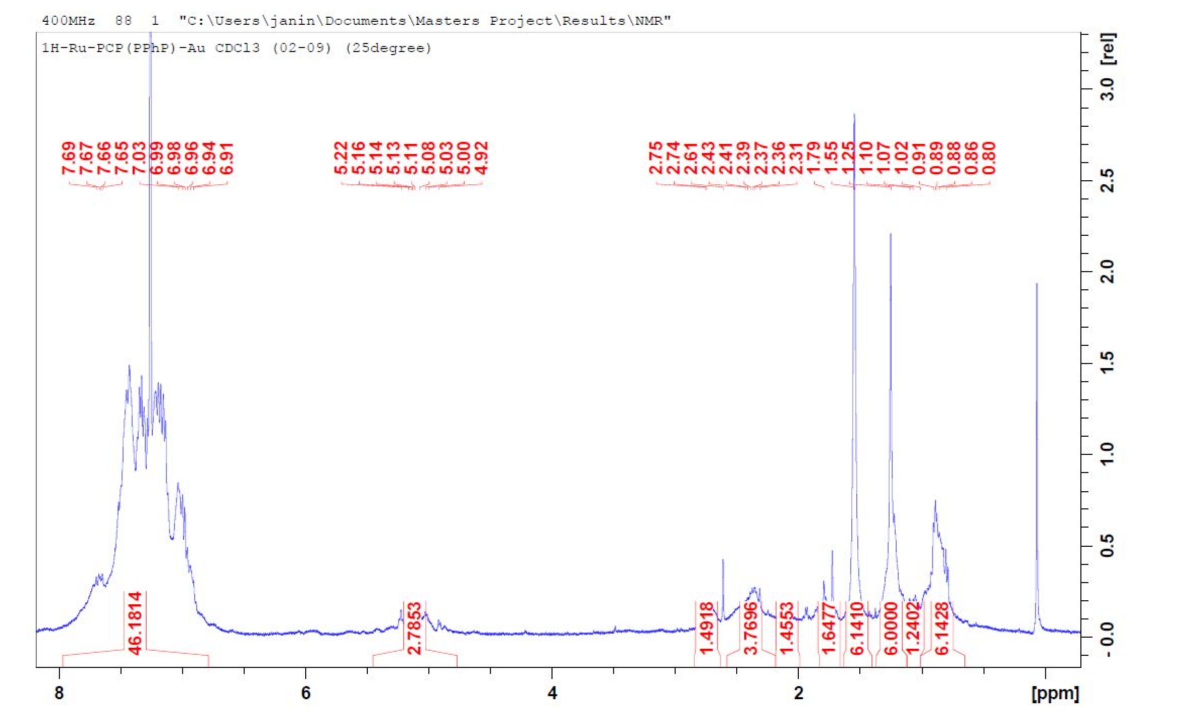

A3 -Summary of $^{31}P\{H\}$ NMR data

Table S1. Summary of the $^{31}P\{H\}$ NMR shifts for the metal complexes of interest.

	**Complex Name**	**$^{31}P\{H\}$ NMR shifts**		
		**Ru(II)-$PC_{(n)}P$ Complexes**		
		Ru-***P***Ph_3	Ru-***P***CP	Ru-PC***P***
**1a**	Ru(II)-$PC_{(1)}P \cdot SbF_6$	23.0 (d, *J* = 13.5 Hz)	22.9 (d, *J* = 27.8 Hz)	-29.3 (dd, *J* = 27.9, 13.7 Hz)
**2a**	Ru(II)-$PC_{(2)}P \cdot SbF_6$	23.4 (s)	23.7 (d, *J* = 30.4 Hz)	-14.7 (dd. *J* = 29.3, 3.9)
**3a**	Ru(II)-$PC_{(3)}P \cdot SbF_6$	23.4 (d, *J* = 52.2 Hz)	18.7 (d, *J* = 52.4 Hz)	-19.3 (s)
**4a**	Ru(II)-$PC_{(4)}P \cdot SbF_6$	23.0 (d, *J* = 52.7 Hz)	18. 8 (d, *J* = 52.4 Hz)	-16.4 (s)
**5a**	Ru(II)-$PC_{(2)}PC_{(2)}P \cdot SbF_6$	24.2 – 18.7 (m)		-12.7 - -16.2 (m)
		**Ru(II)-$PC_{(n)}P$-Au(I) Complexes**		
		Ru-***P***Ph_3	Ru-***P***CP	Ru-PC***P***-Au
**1b**	Ru(II)-$PC_{(1)}P$-Au(I)$\cdot SbF_6$	22.4 (d, *J* = 53.10 Hz)	19.5 (dd, *J* = 52.1, 18.4 Hz)	20.3 (d, *J* = 18.9 Hz)
**2b**	Ru(II)-$PC_{(2)}P$-Au(I)$\cdot SbF_6$	23.2 (d, *J* = 6.7 Hz)	23.2 (d, *J* = 28.3 Hz)	30.1 (dd, *J* = 27.4, 7.9 Hz)
**3b**	Ru(II)-$PC_{(3)}P$-Au(I)$\cdot SbF_6$	23.3 (d, *J* = 52.2 Hz)	18.5 (d, *J* = 52.3 Hz)	28.8 (s)
**3c**	Ru(II)-$PC_{(3)}P$-Au(I)$\cdot NO_3$	23.7-22.9 (m)	18.1 (dd, *J* = 135.9, 52.2 Hz)	28.2 (s)
**4b**	Ru(II)-$PC_{(4)}P$-Au(I)$\cdot SbF_6$	22.7 (d, *J* = 52.6 Hz)	19.0 (d, *J* = 51.2 H z)	31.35 (s)
**5b**	Ru(II)-$PC_{(2)}PC_{(2)}P$-Au(I)$\cdot SbF_6$	22.9 - 22.7 (m)	23.9 – 23.5 (m)	34.1 - 33.1 (m), 31.8-29.7 (m)

A4 - $^{31}P\{H\}$ NMR Spectra

Starting Materials

Figure S15. $^{31}P\{H\}$ NMR Spectrum of Ru(II)-PPh3

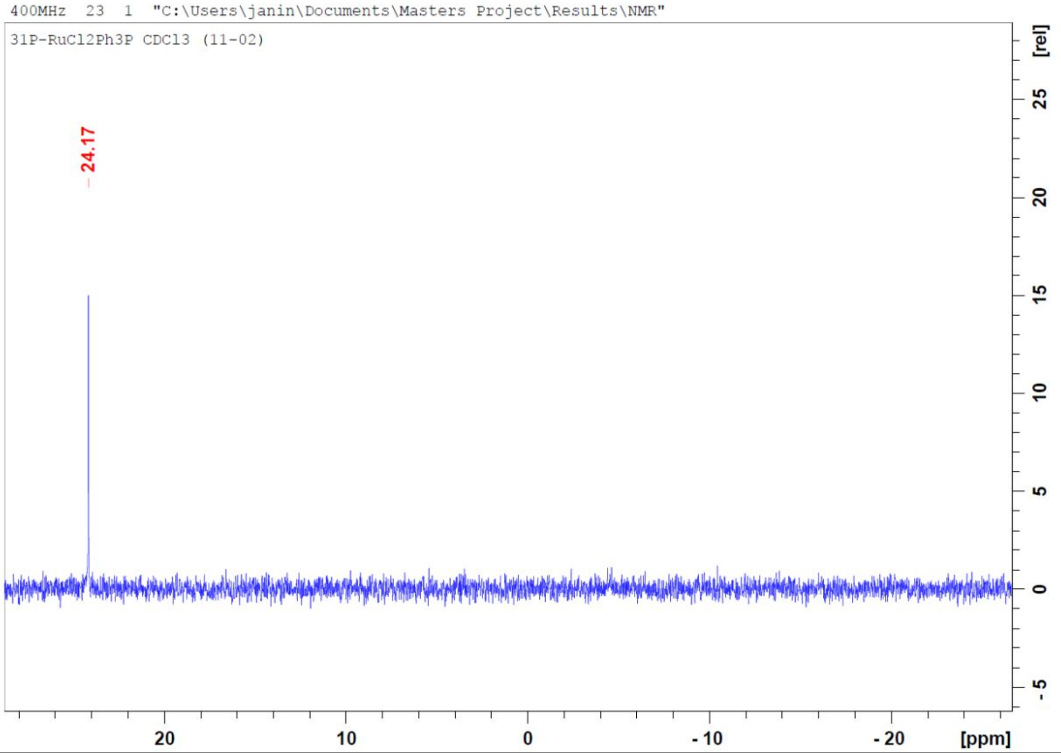

Figure S16. $^{31}P\{H\}$ NMR Spectrum of Ru(II)-acetonitrile

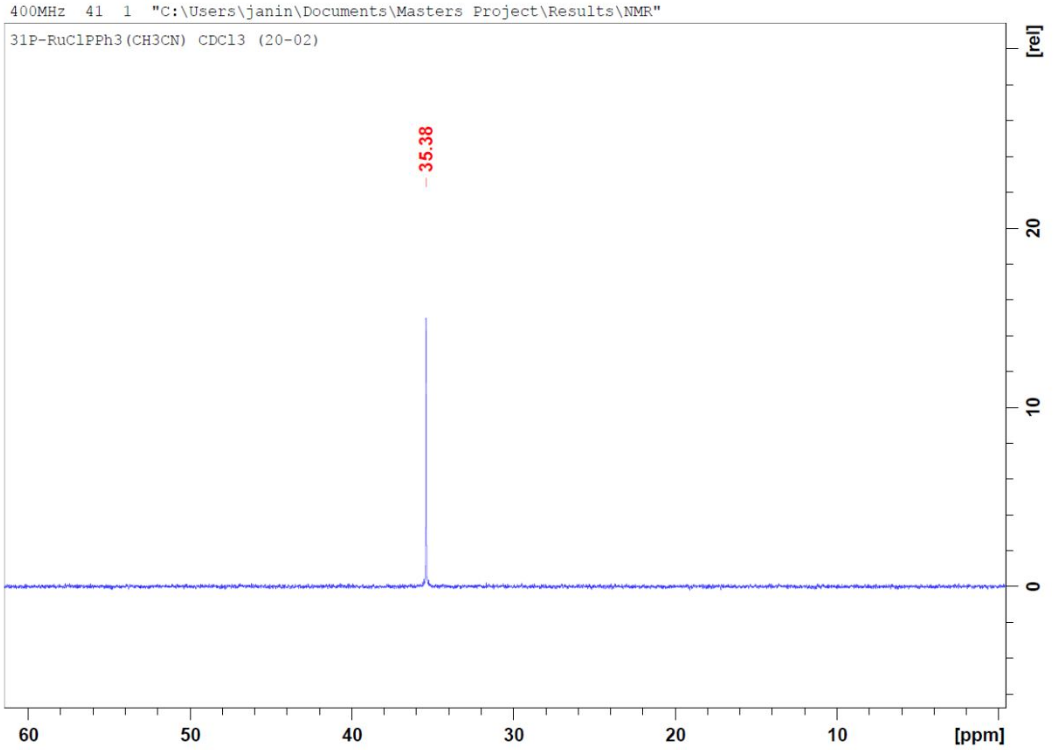

Ru(I)-PC$_{(n)}$P Complexes

Figure S17. $^{31}P\{H\}$ NMR Spectrum of Ru(II)-PC$_{(1)}$P·SbF_6

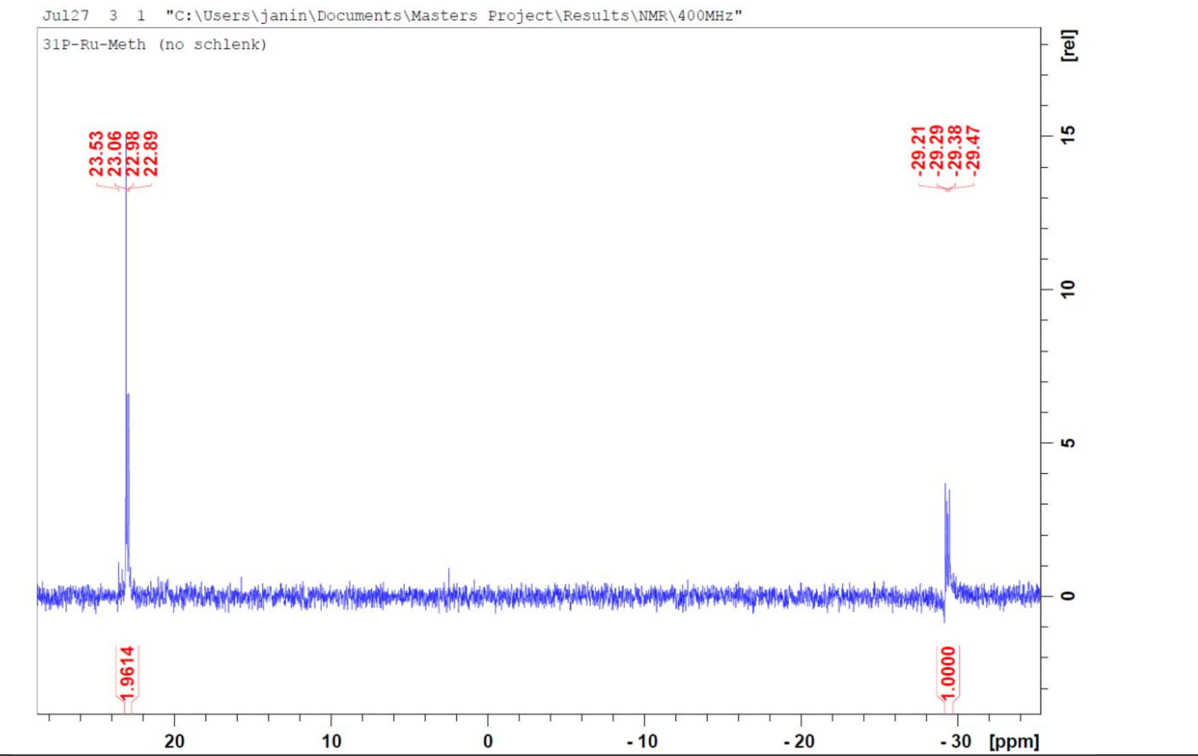

Figure S18. $^{31}P\{H\}$ NMR Spectrum of Ru(II)-$PC_{(2)}P\cdot SbF_6$

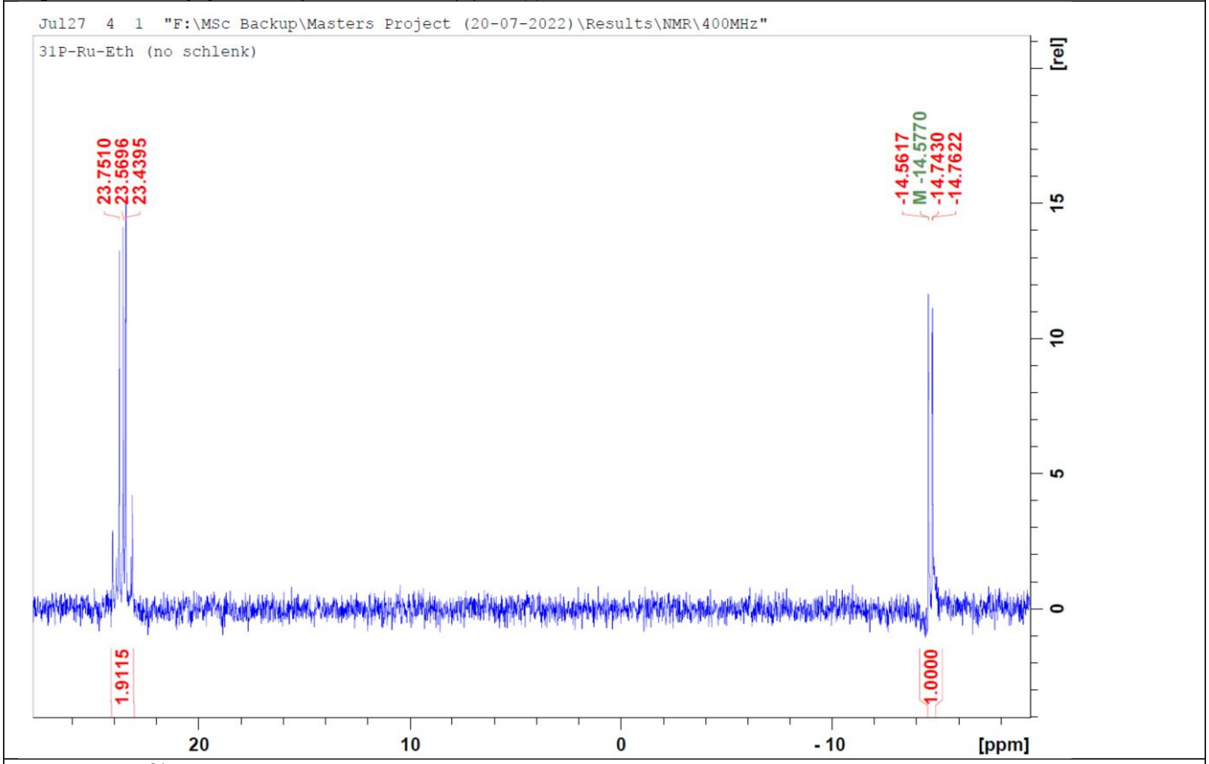

Figure S19. $^{31}P\{H\}$ NMR Spectrum of Ru(II)-$PC_{(3)}P\cdot SbF_6$

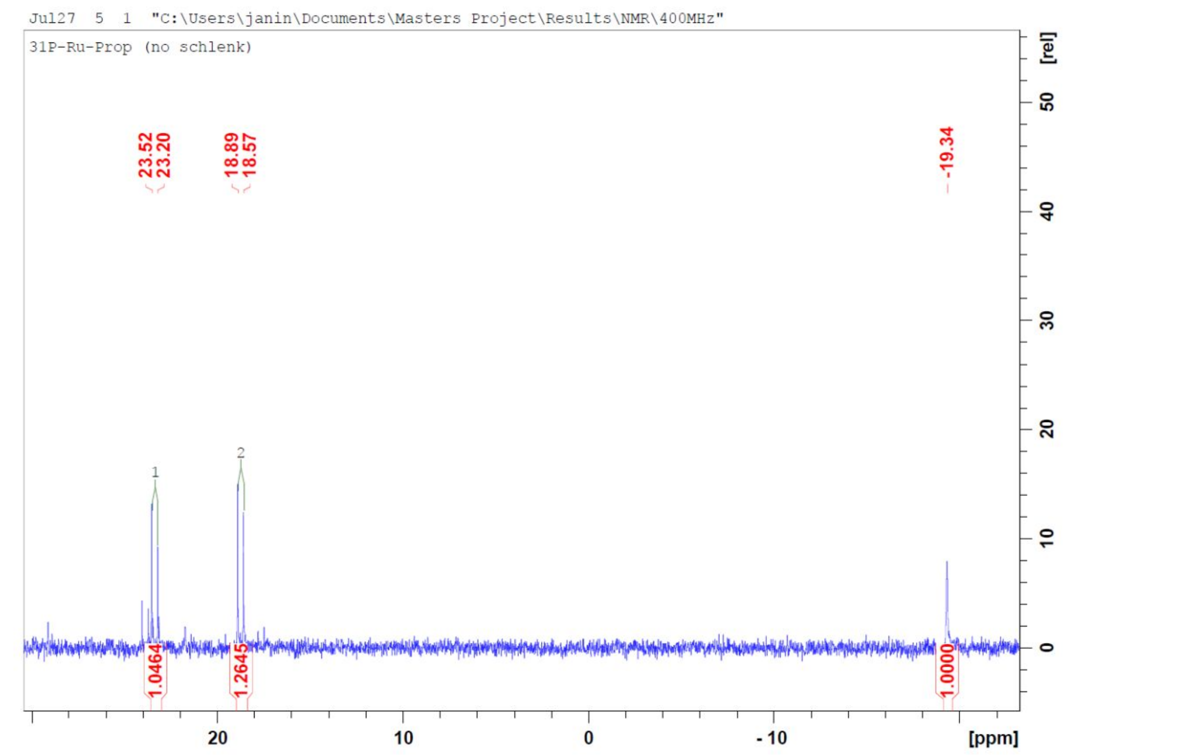

Figure S20. $^{31}P\{H\}$ NMR Spectrum of Ru(II)-$PC_{(4)}P \cdot SbF_6$

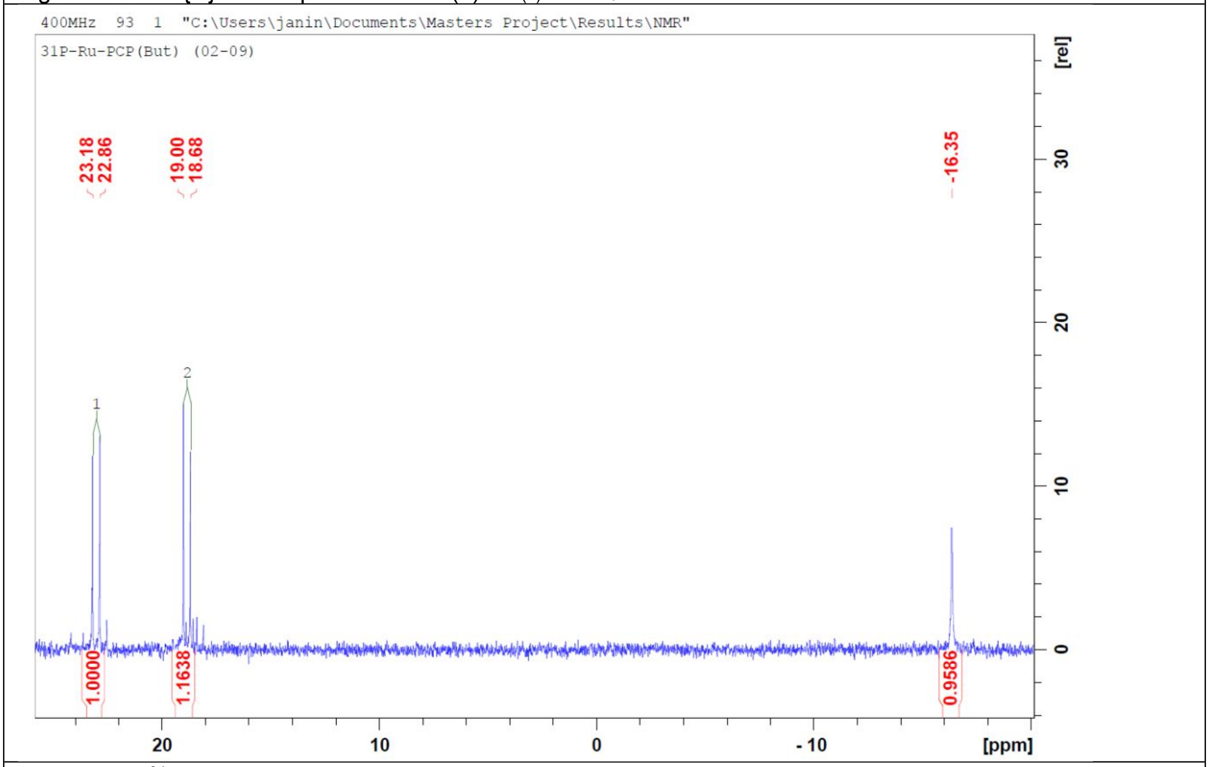

Figure S21. $^{31}P\{H\}$ NMR Spectrum of Ru(II)-$PC_{(2)}PC_{(2)}P \cdot SbF_6$

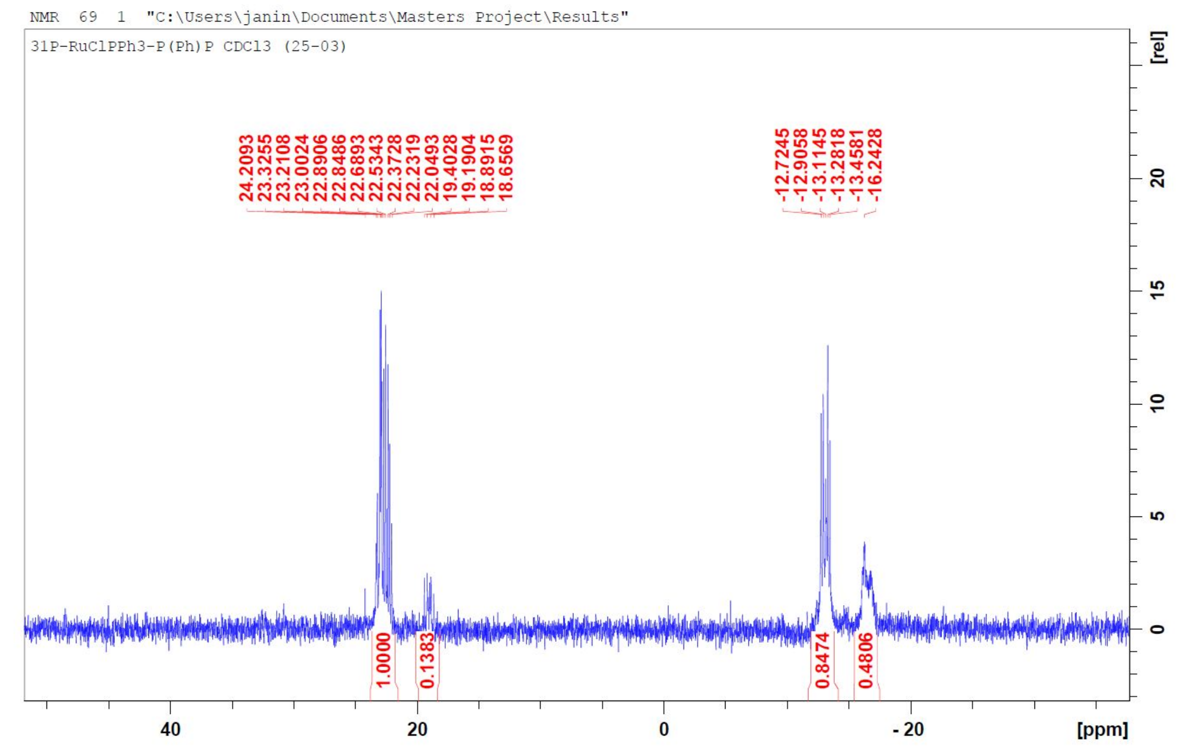

Ru(I)-PC$_{(n)}$P-Au(I) Complexes

Figure S22. ^{31}P{H} NMR Spectrum of Ru(II)-PC$_{(1)}$P-Au(I)·SbF$_6$

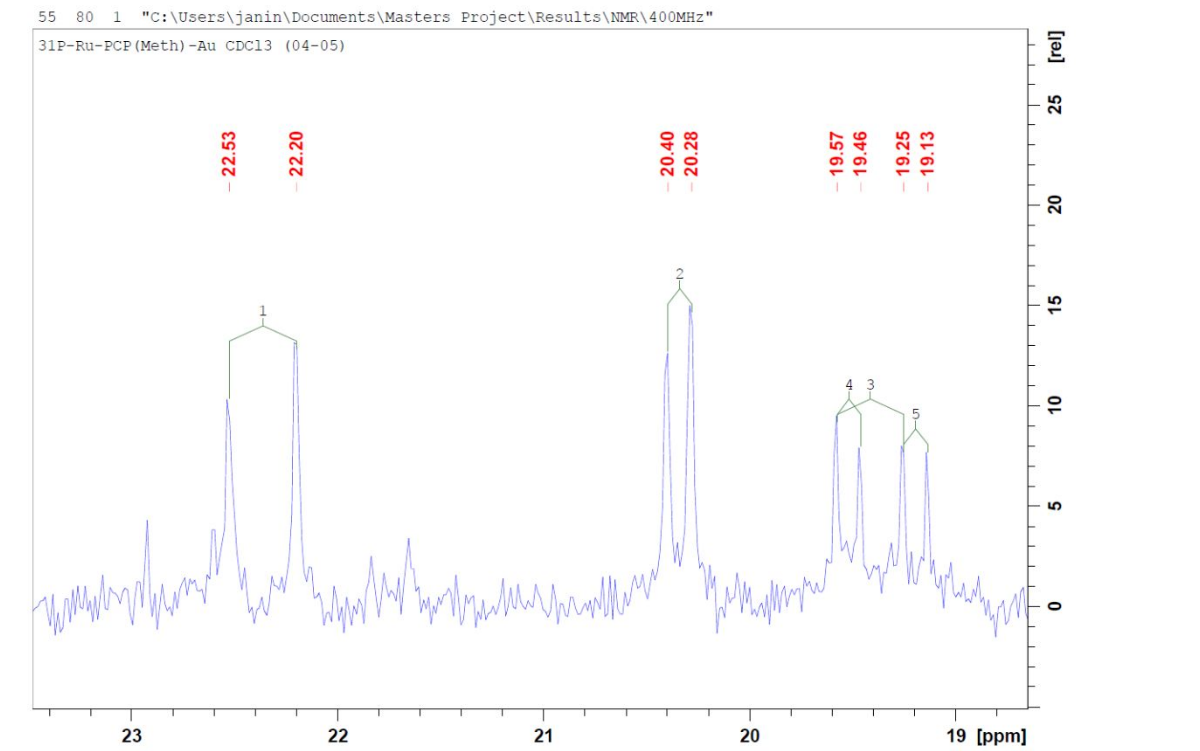

Figure S23. ^{31}P{H} NMR Spectrum of Ru(II)-PC$_{(2)}$P-Au(I)·SbF$_6$

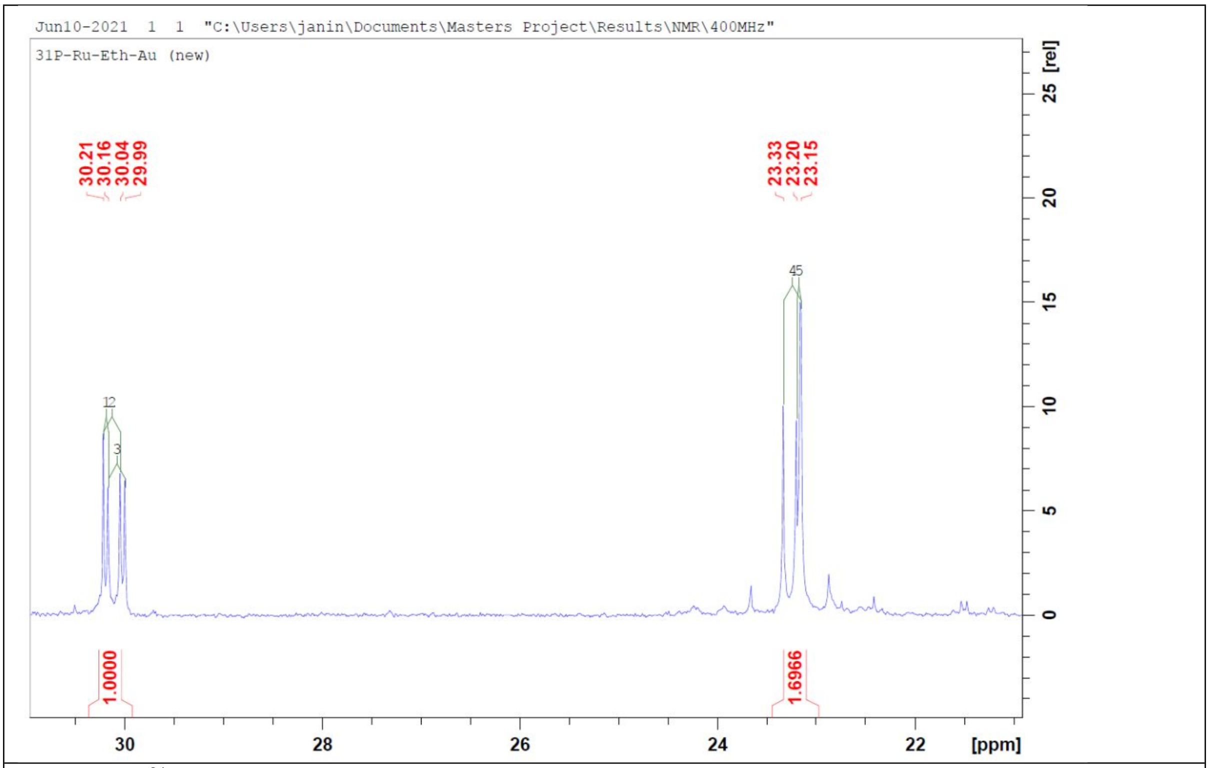

Figure S24. $^{31}P\{H\}$ NMR Spectrum of Ru(II)-PC$_{(3)}$P-Au(I)·SbF_6

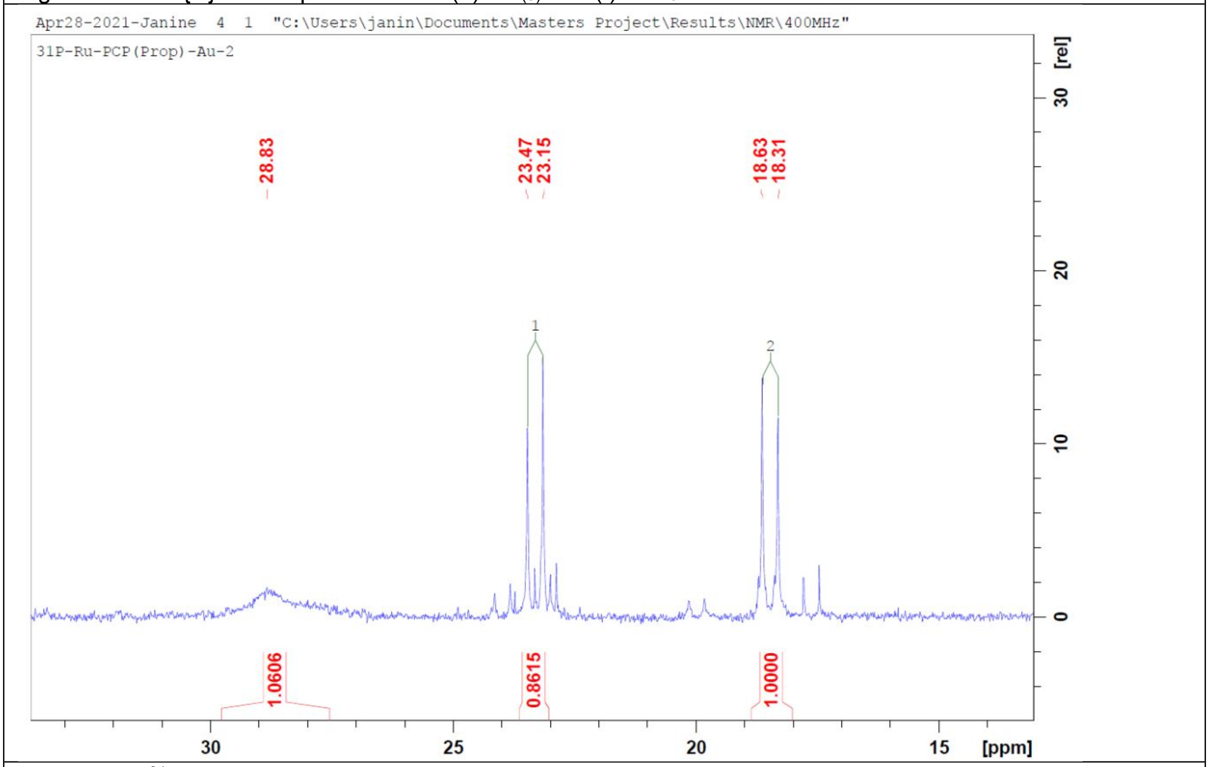

Figure S25. $^{31}P\{H\}$ NMR Spectrum of Ru(II)-PC$_{(3)}$P-Au(I)·NO_3

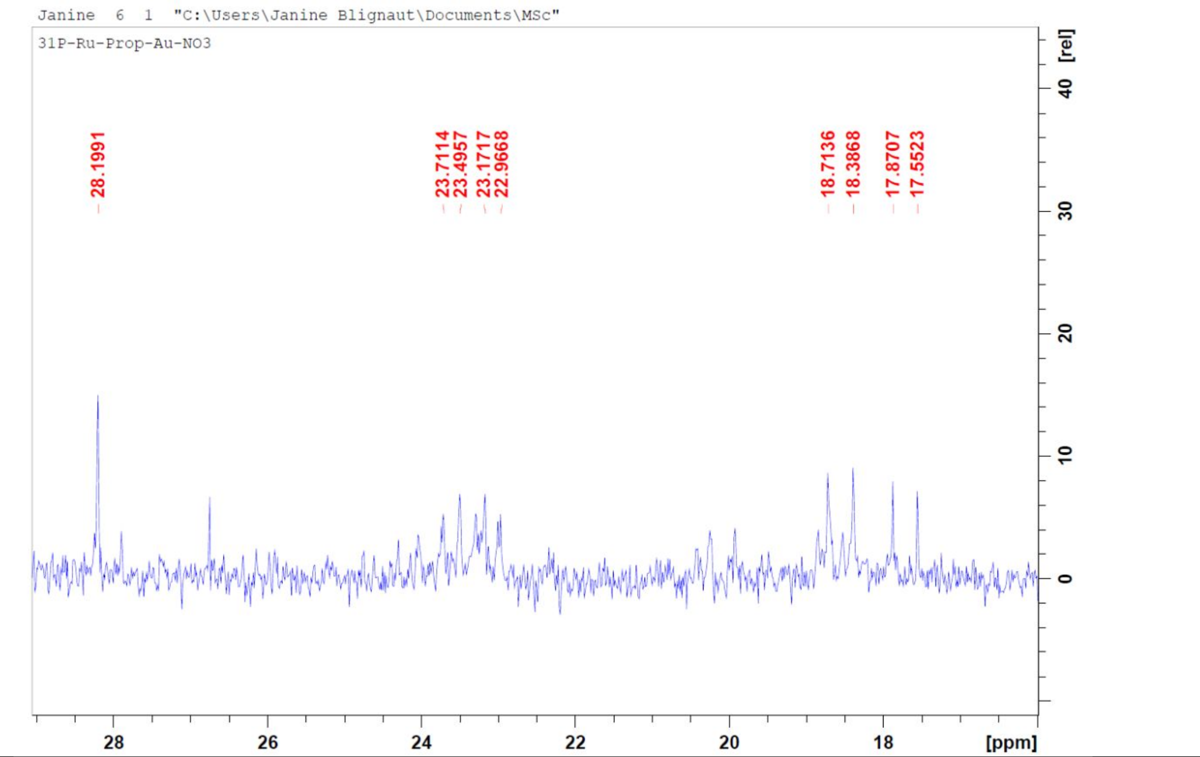

Figure S26. ^{31}P{H} NMR Spectrum of Ru(II)-PC(4)P-Au(I)·SbF_6

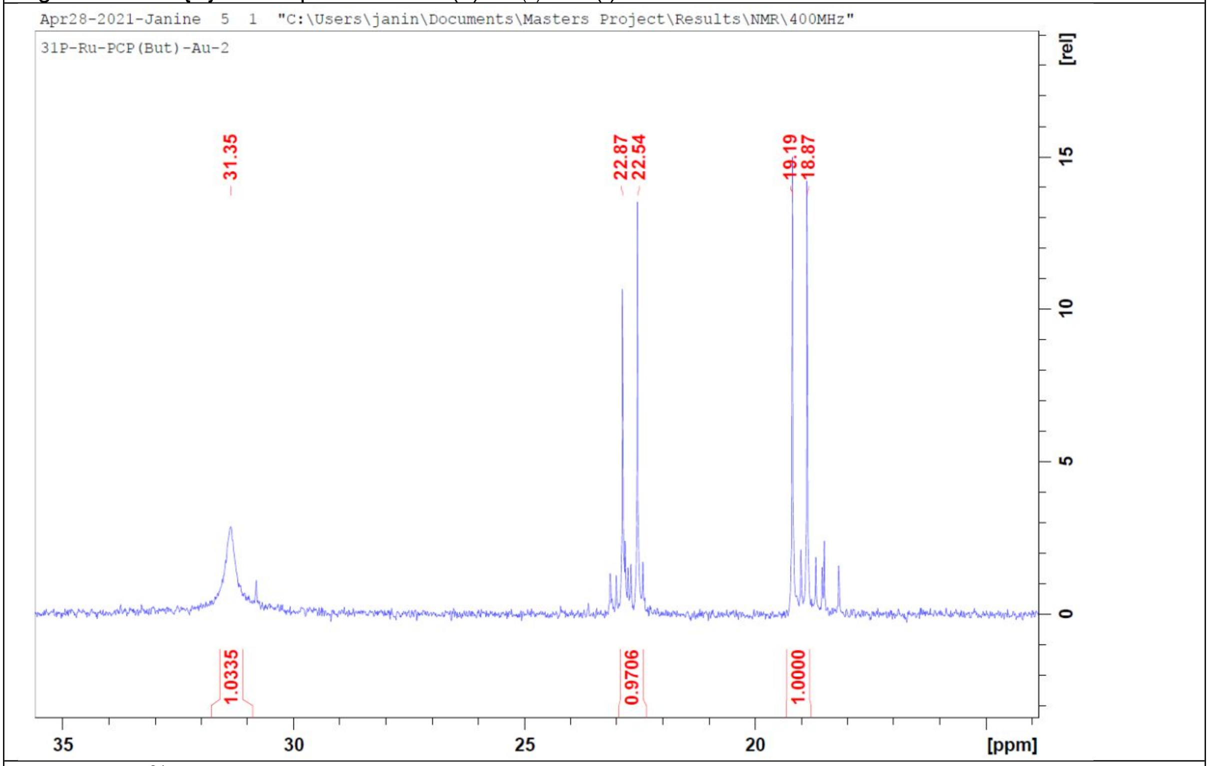

Figure S27. ^{31}P{H} NMR Spectrum of Ru(II)-PC(2)PC(2)P-Au(I)·SbF_6

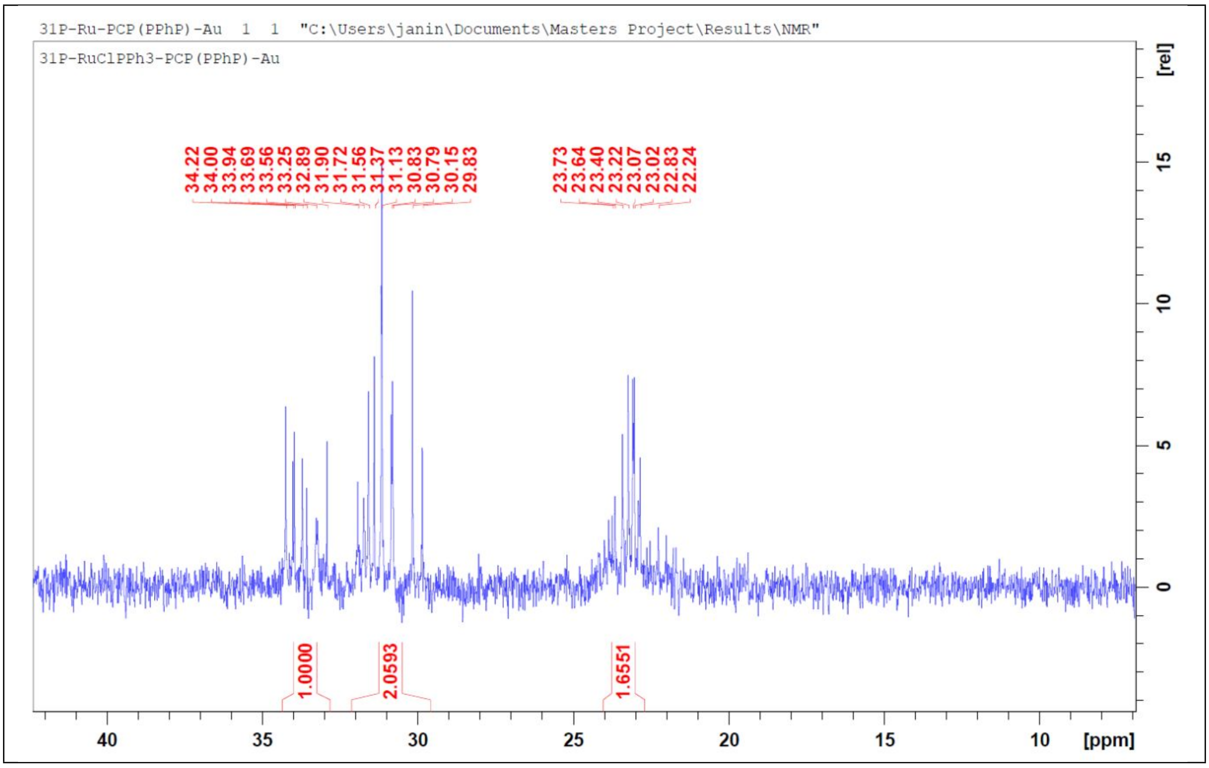

31P-Ru-PCP(PPhP)-Au 1 1 "C:\Users\janin\Documents\Masters Project\Results\NMR"
31P-RuClPPh3-PCP(PPhP)-Au
34.22 34.00 33.94 33.69 33.56 33.25 32.89 31.90 31.72 31.56 31.37 31.13 30.83 30.79 30.15 29.83
23.73 23.64 23.40 23.22 23.07 23.02 22.83 22.24
1.0000
2.0593
1.6551
[rel]
[ppm]

A5 - ESI-MS Spectra

Ru(I)-PC$_{(n)}$P Complexes

Figure S28. ESI-MS Spectrum of Ru(II)-PC$_{(1)}$P·SbF$_6$

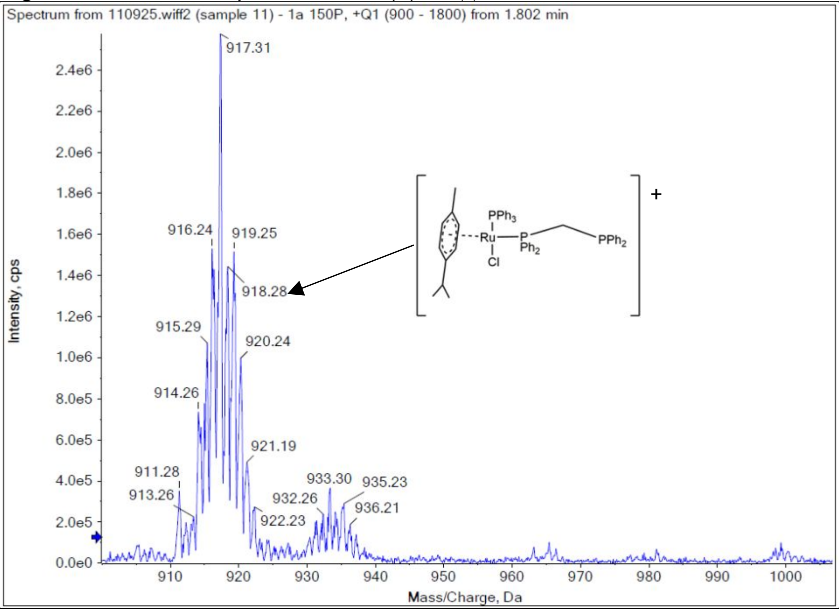

Figure S29. ESI-MS Spectrum of Ru(II)-PC$_{(2)}$P·SbF$_6$

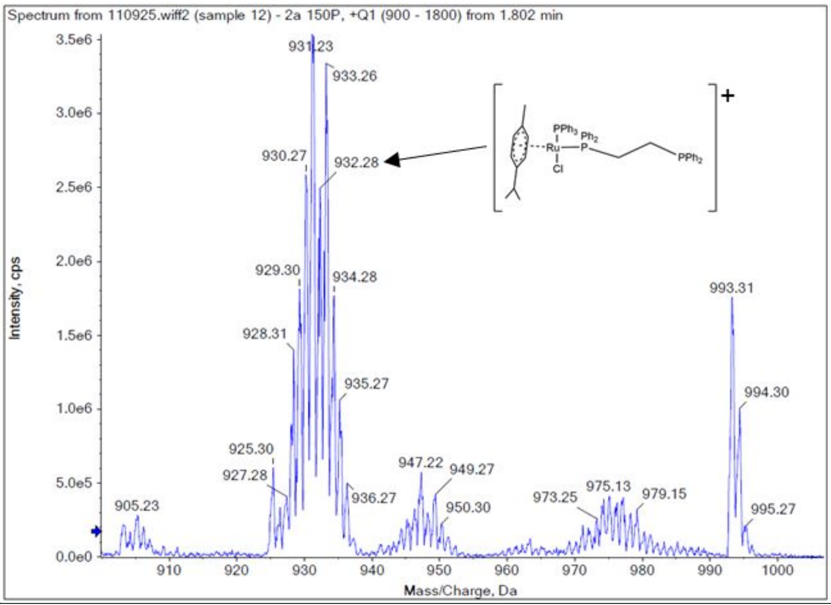

Figure S30. ESI-MS Spectrum of Ru(II)-PC$_{(3)}$P·SbF$_6$

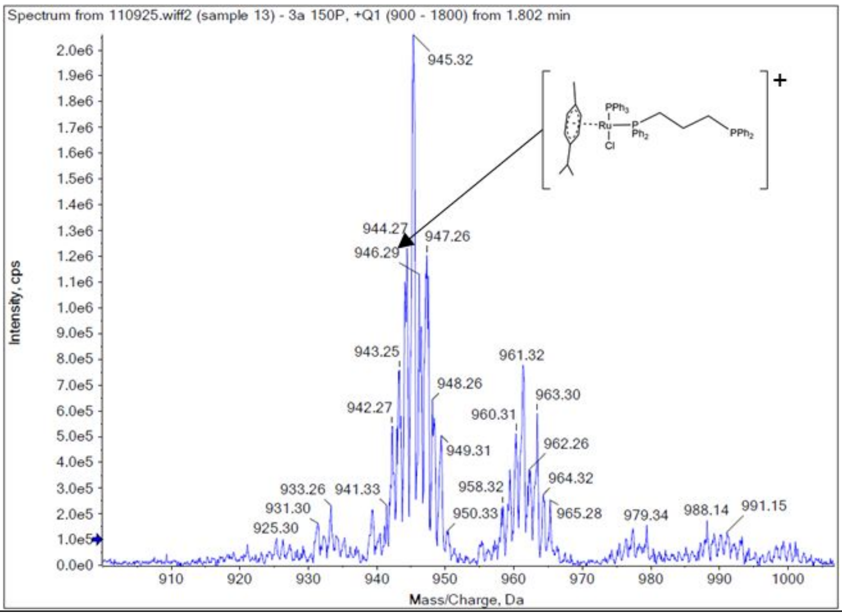

Figure S31. ESI-MS Spectrum of Ru(II)-PC(4)P·SbF6

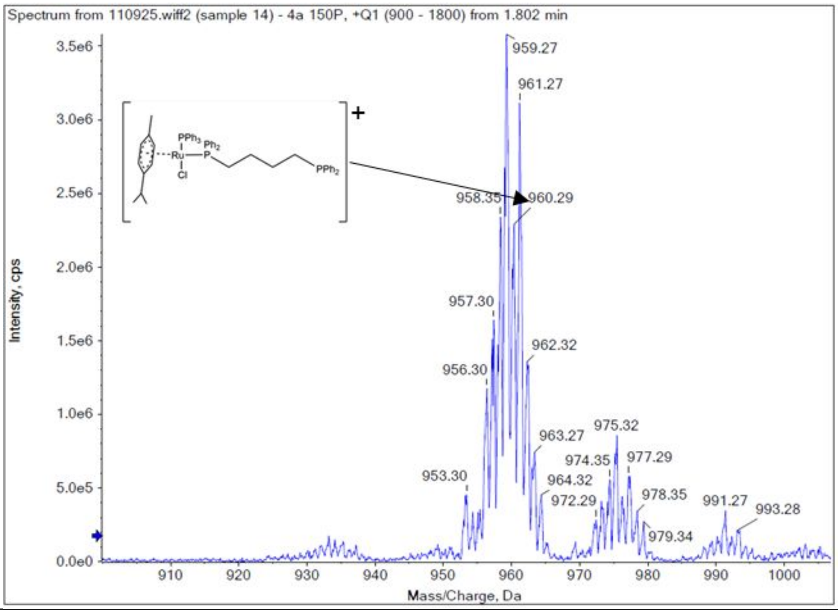

Figure S32. ESI-MS Spectrum of Ru(II)-PC(2)PC(2)P·SbF6

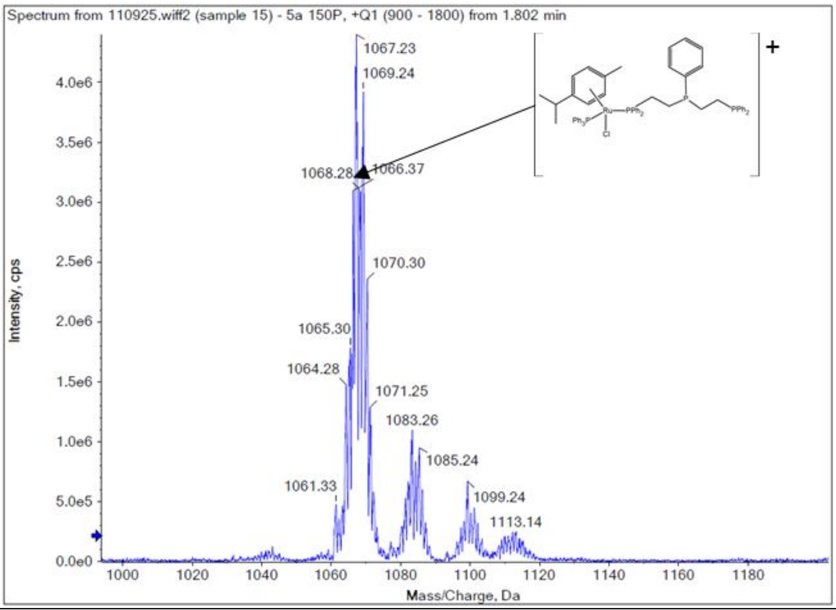

Ru(I)-PC$_{(n)}$P-Au(I) Complexes

Figure S33. ESI-MS Spectrum of Ru(II)-PC$_{(1)}$P-Au(I)·SbF_6

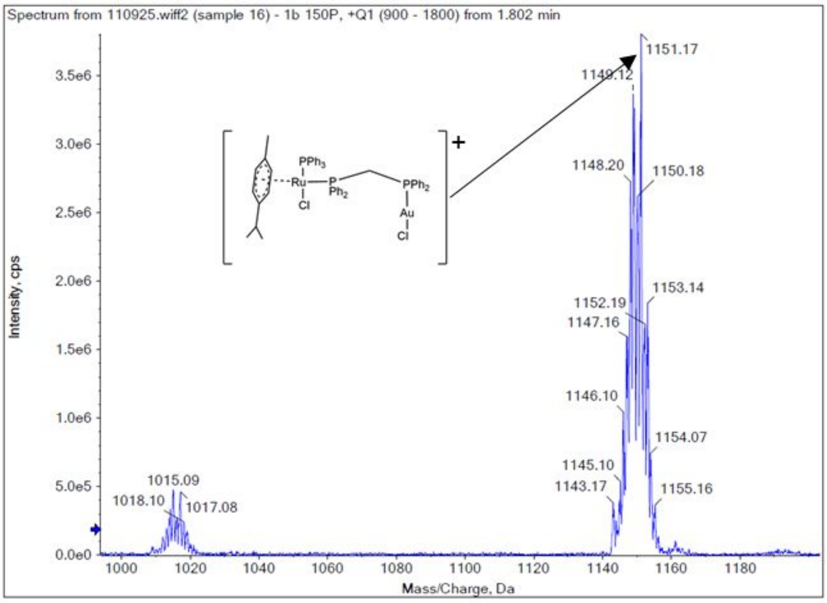

Figure S34. ESI-MS Spectrum of Ru(II)-PC$_{(2)}$P-Au(I)·SbF_6

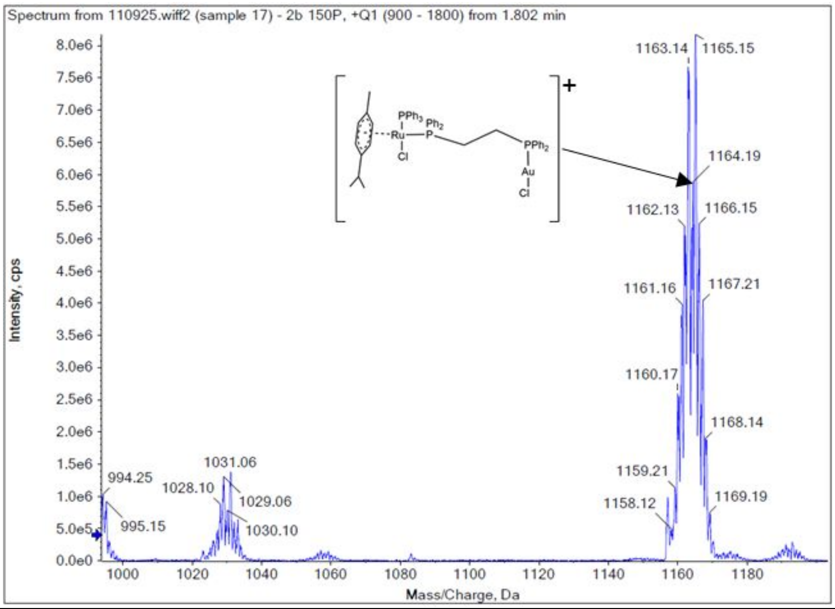

Figure S35. ESI-MS Spectrum of Ru(II)-PC(3)P-Au(I)·SbF6

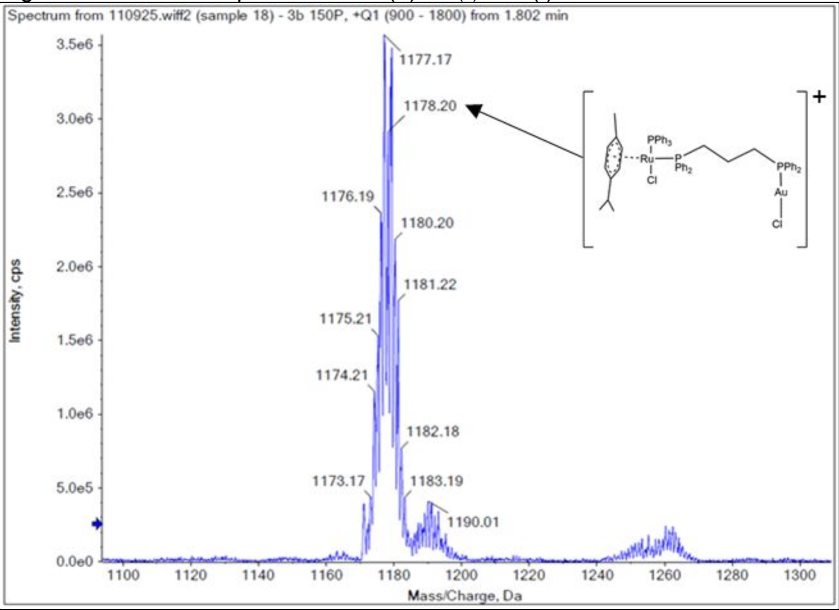

Figure S36. ESI-MS Spectrum of Ru(II)-PC(3)P-Au(I)·NO3

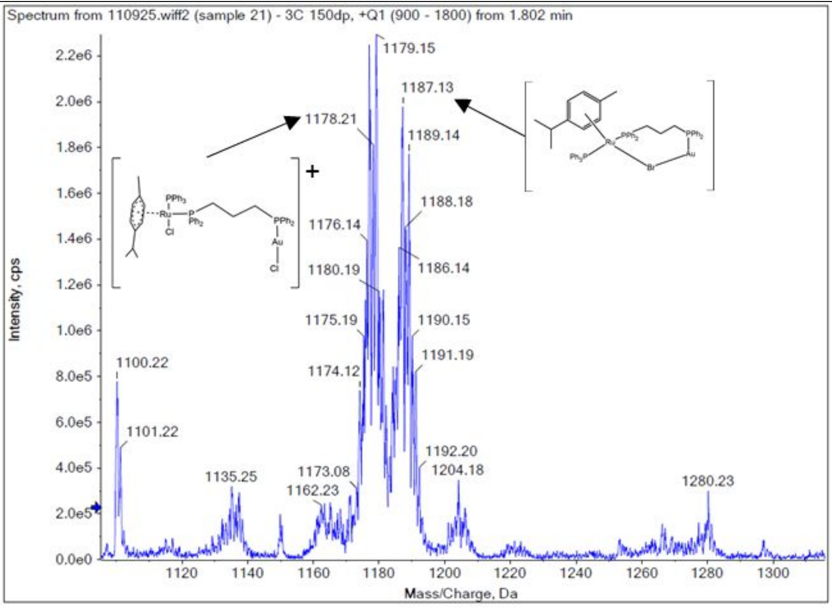

Figure S37. ESI-MS Spectrum of Ru(II)-PC$_{(4)}$P-Au(I)·SbF$_6$

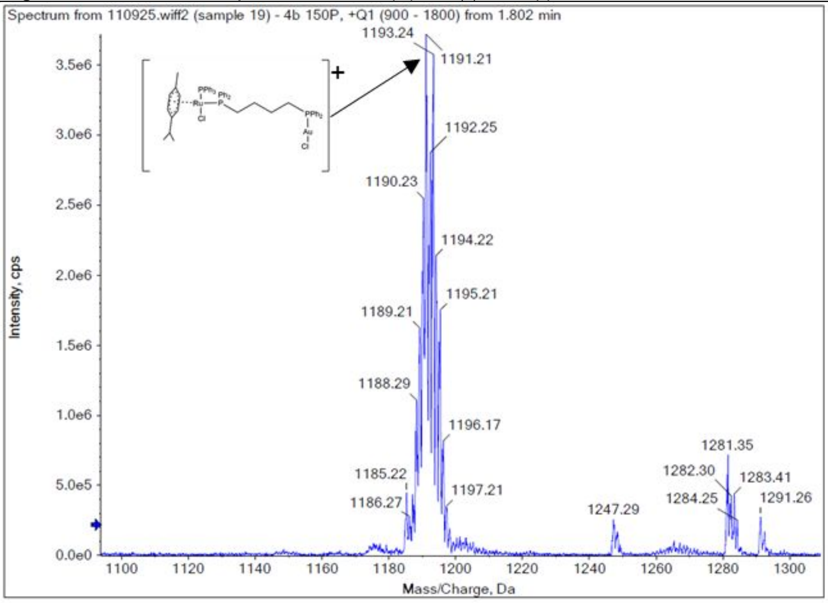

Figure S38. ESI-MS Spectrum of Ru(II)-PC$_{(2)}$PC$_{(2)}$P-Au(I)·SbF$_6$

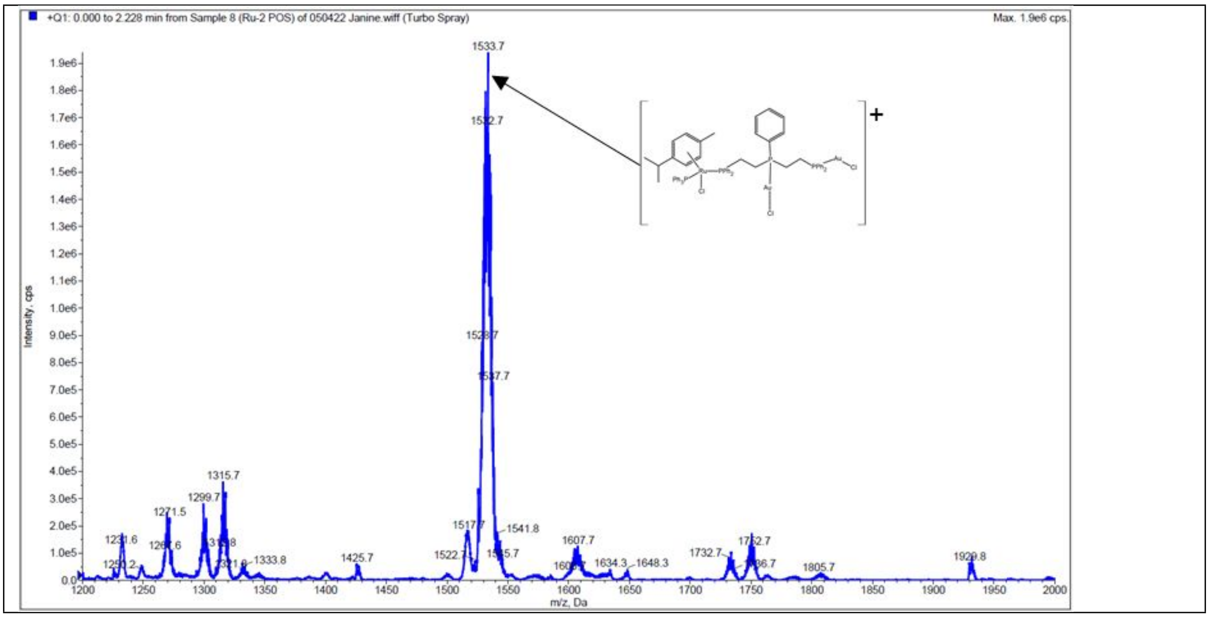

+Q1: 0.000 to 2.228 min from Sample 8 (Ru-2 POS) of 050422 Janine.wiff (Turbo Spray)
Max. 1.9e6 cps.
Intensity, cps
m/z, Da
1533.7
1532.7
1528.7
1537.7
1315.7
1299.7
1271.5
1231.6
1517.7
1541.8
1425.7
1333.8
1607.7
1634.3
1648.3
1732.7
1752.7
1805.7
1929.8

A6 - X-Ray Diffraction Discussion

Crystals of η[1]-1,1-*bis*(diphenylphosphino)methan-η[6]-*p*-cymenetriphenylphosphino-chlorido-ruthenium(II) hexafluoroantimonate(I) (**1a**) were obtained from a 50:50 DCM:diethyl ether solution. **1a** crystallised in the monoclinic $P2_1/n$ space group as a cationic complex with a central ruthenium(II) atom coordinated to a *p*-cymene, triphenylphosphine, monodentate *bis*(diphenylphosphino)methane, and a chlorido ligand, an anionic SF_6^- counter ion, and one DCM solvent molecule (see Figure S38). The *p*-cymene group coordinates to the metal atom through a η^6 π-bond, forming the *pseudo*-octahedral geometry or a piano-stool configuration. [18], [65], [66], [67], [68] The *bis*(diphenylphosphino)methane ligand has one carbon atom coordinated to two diphenylphosphine ligands, with one phosphine atom coordinated to the ruthenium(II) metal in a monodentate fashion and the other phosphine atom left pendant. In **1a**, the Ru1-P1 bond (2.3649(15) Å) is slightly shorter than the Ru1-P2 (2.3859(16) Å) bond, the Ru1-Cl1 bond is the longest bond (2.3924(14) Å) in the coordination sphere, and Ru1-Cg1 is reported as 1.7835(6) Å, all of which compares well with similar structures in literature (Table S1). [67] The angles around the Ru(II) centre are reported as 128.44(4) °, 125.53(4) °, and 122.76(4) ° for Cg1-Ru1-P1, Cg1-Ru1-P2, and Cg1-Ru1-Cl1 respectively. Nine hydrogen bonding and seven π-interactions are observed in the structure of **1a** (see Table S2 and Figure S39 and S40). The hydrogen bonding and π-interactions range between 3.124(7) Å and 3.437(6) Å (D…A) and between 3.415(6) Å and 3.886(6) Å (D…Cg) respectively. Interestingly, eight of the nine hydrogen bonding interactions observed in **1a** involves the SbF_6^- counter ion and DCM solvent molecule. Five of the seven aromatic rings in the structure (from the PPh_3 and one of the PPh_2 ligands) are involved in the π-interactions. **1a** is further stabilised by bifurcation between Cl1 and two hydrogen atoms (H11A and H25). Molecules of **1a** pack in a head-to-tail fashion when viewed along the b-axis (Figure S41).

[μ-1,1-*Bis*(diphenylphosphino)methan-η6-p-cymenetriphenylphosphino-chloridoruthenium(II)]chloridogold(I) hexafluoroantimonate(I) (**1b**) was obtained from a 50:50 DCM:methanol solution. In **1b**, the 1,1-bis(diphenylphosphino)methane ligand, is coordinated to Ru(II) on one side and Au(I) on the other. **1b** crystallized with two complex molecules, two SbF_6^- counterions (one on a special position, Sb2 [0.5 1 0.5] and Sb3 [1 0 1]), and two DCM solvent molecules in the asymmetric unit (Figure S44, Table 3). As with **1a**, the Ru(II) metal centre is coordinated to a *p*-cymene, a

triphenylphosphine ligand, a *bis*(diphenylphosphino)methane, and a chlorido ligand. All the bond distances and angles compare well to **1a** and similar structures in literature (Table S3), with P3-Au1 and Au1-Cl2 bond distances reported as 2.220(3) Å and 2.287(3) Å, respectively, and the P3-Au1-Cl2 angle almost linear at 174.77(11)°. [18] Fourteen hydrogen bonding interactions (of which eight are intramolecular), seven D-H…π interactions (of with five are intramolecular), two D-X…π interactions, and two π - π interactions are observed in the structure of **1b** (Table S4 and Figures S45 and S46). All fourteen of the hydrogen bonding interactions involve the counter ion or solvent molecule. **1b** is further stabilised by bifurcation at Cl1 (from H11B and H29) and Cl3 (from H110B and H84), and an intramolecular short contact (4.153(4) Å) between Sb1 and Cl6 forming an infinite chain along the c-axis (Figure S47). **1b** packs in a head-to-head fashion in column like structures with the counter ions and solvent molecules systematically positioned in between the columns, when viewed along the b,c-plane (Figure S47).

[μ-1,2-*Bis*(diphenylphosphino)ethane-η^6-*p*-cymenetriphenylphosphinochlorido-ruthenium(II)]chloridogold(I) hexafluoroantimonate(I) (**2b**) was obtained from a 50:50 DCM:Methanol solution. **2b** crystallised in the triclinic $P\bar{1}$ space group with one complex molecule and one SbF_6^- counterion in the asymmetric unit (Figure S13, Table 3). The ruthenium(II) centre is coordinated to a *p*-cymene, a triphenylphosphine ligand, a *bis*(diphenylphosphino)ethane, and a chlorido ligand. The Ru(II) centre is bridged to a chloridogold(I) moiety through the pendant phosphine atom of the 1,1-bis(diphenylphosphino)ethane ligand. The bond distances and angles compare well with those reported for similar structures in literature [18] as well as with **1b** (Table S5), with P3-Au1 and Au1-Cl2 bond distances reported as 2.2323(15) Å and 2.2929(14) Å, respectively, and the P3-Au1-Cl1 angle again almost linear at 175.12(5) °. Twelve hydrogen bonding interactions, of which six are intramolecular (Figure S51), and three intramolecular C-H…π-interactions (Figure S52) are observed in the structure of **2b**. The ruthenium bound chlorido ligand is the acceptor of five of the six intramolecular hydrogen bonding interactions, significantly stabilising the crystal structure of **2b** (Table S6). **2b** packs in a head-to-head fashion with the SbF_6^- counter ions in between, when viewed along the a-axis (Figure S53).

[μ-1,3-*Bis*(diphenylphosphino)propan-η^6-*p*-cymene-triphenylphosphinochlorido-ruthenium(II)]chloridogold(I) hexafluoroantimonate(I) was recrystallized from a 50:50 DCM:methanol solution. The structure of **3b** is similar to

that of **1b** and **2b** but has a PC_3P bridge (Figure S56). **3b** crystallized in the triclinic *P*1 space group with one complex molecule and one SbF_6^- counter ion in the asymmetric unit. One of the phenyl rings on P3 displays a positional disorder in a 39:61 ratio, as well as two fluorine atoms in SbF_6^- (F3 in a 32:68 ratio and F2 in a 35:65 ratio). The bond distances and angles compare well with those reported for similar structures in literature [18] as well as with **1b** and **2b** (Table S7), with P3-Au1 and Au1-Cl2 bond distances reported as 2.226(4) Å and 2.267(4) Å, respectively, and the P3-Au1-Cl2 angle the most linear at 178.5(2) °. Five hydrogen bonding interactions and seven π-interactions are observed in the structure of **3b** (Figures S57 and S58, Table S8). Three of the hydrogen bonding interactions are to three SbF_6^- counter ions (one intramolecular and two intermolecular), while Cl1 is once again the acceptor of two intramolecular hydrogen bonding interactions. All of this contributes to the stabilisation of **3b**. The molecules pack in a head-to-toe fashion along the b-axis, with the Ru(II) centre of both molecules (created by a glide plane along the b-axis) situated at a corner of the unit cell and the counter ions forming 'layers' in between the complex molecules (Figure S59).

A7 - X-Ray Diffraction and Hirshfeld data

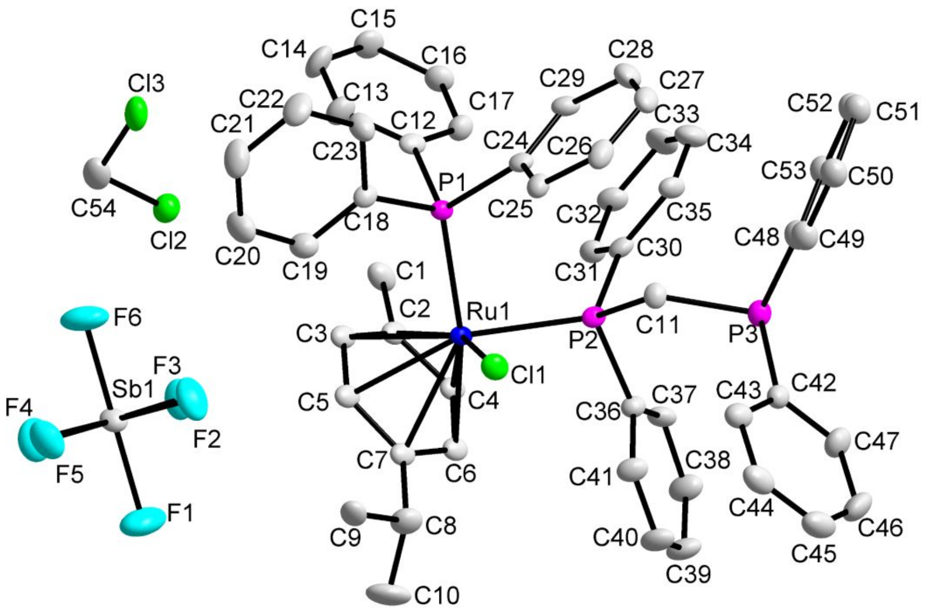

Figure S39. Illustration of the molecular structure of **1a**. Ellipsoids are drawn at 50 % probability, and H-atoms were omitted for clarity.

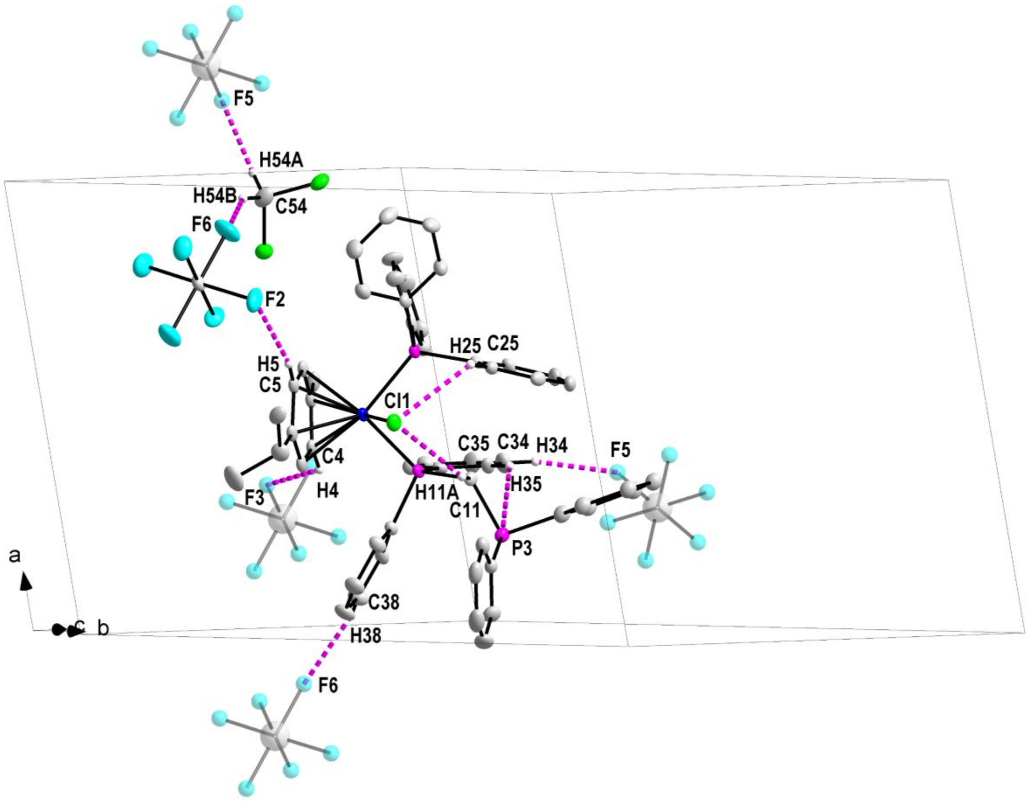

Figure S40. The hydrogen bonding interactions observed in the structure of **1a**, illustrated in pink dashed lines. Ellipsoids are drawn at 50 % probability, and insignificant labels and H-atoms were omitted for clarity.

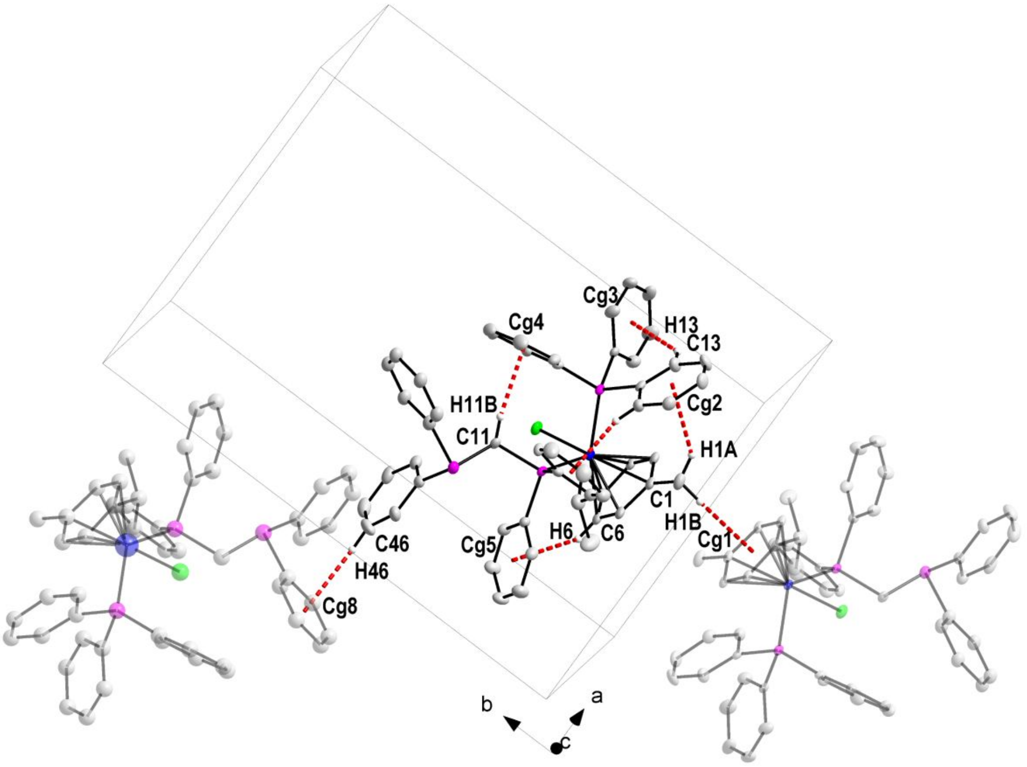

Figure S41. The π-interactions observed in the structure of **1a,** illustrated in red dashed lines. Ellipsoids are drawn at 50 % probability, and insignificant labels and H-atoms were omitted for clarity.

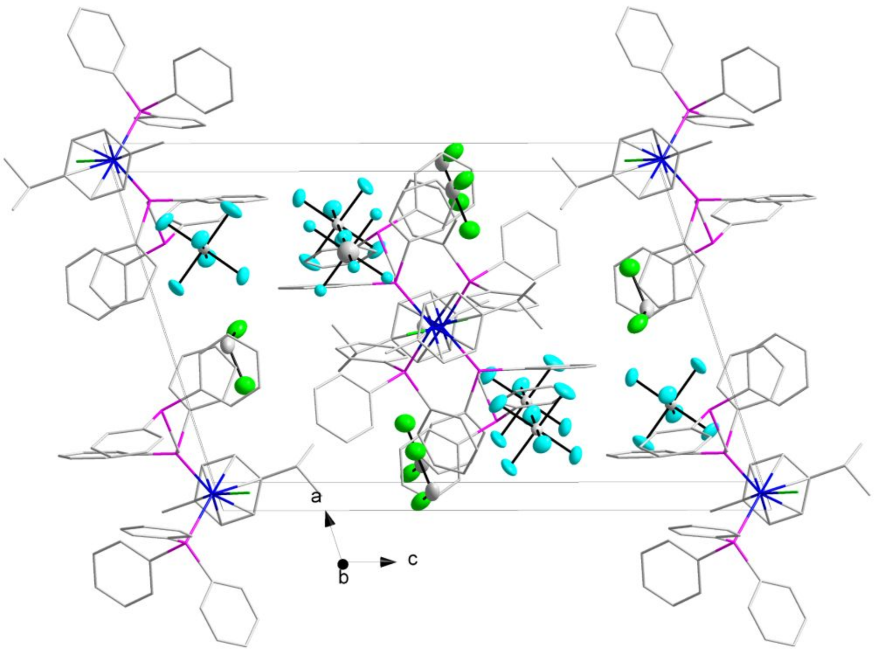

Figure S42. Illustration of the packing of **1a** in the unit cell. Ellipsoids are drawn at 50 % probability, and labels and H-atoms were omitted for clarity.

Table S2. Selected bond lengths and angles of **1a**.

**Bonds**	**Bond lengths (Å)**	**Angles**	**Bond angles**
Ru1-Cl1	2.392(1)	P1- Ru1-Cl1	86.42(5)
Ru1-P1	2.365(2)	P2- Ru1-Cl1	87.23(5)
Ru1-P2	2.386(2)	P1- Ru1-P2	93.99(5)
Ru1-Cg1*	1.7835(6)	P2-C11-P3	114.9(3)
P2-C11	1.838(5)	Cg1*-Ru1-P1	128.44(4)
P3-C11	1.877(5)	Cg1*-Ru1-P2	125.53(4)
		Cg1*-Ru1-Cl1	122.76(4)

**Cg1 is the centre of gravity of the C2 - C7 carbon ring.*

Table S3. Hydrogen bonding interactions and π-interactions observed in the structure of **1a**.

**D-H…A**	**d(D-H) Å**	**D(H…A) Å**	**d(D…A) Å**	**<(DHA) °**
**C4-H4…F3**[a]	0.95	2.51	3.124(7)	122
**C5-H5…F2**	0.95	2.40	3.299(7)	158
**C11-H11A…Cl1**	0.99	2.66	3.319(6)	124
**C25-H25…Cl1**	0.95	2.77	3.342(6)	119

**C34-H34…F5[b]**	0.95	2.39	3.302(7)	161
**C35-H35…P3**	0.95	2.87	3.437(6)	120
**C38-H38…F6[c]**	0.95	2.46	3.226(7)	138
**C54-H54A…F5[d]**	0.99	2.39	3.336(9)	160
**C54-H54B…F6**	0.99	2.30	3.178(9)	146

**D-H…Cg**	**d(D-H) Å**	**D(H…Cg) Å**	**d(D…Cg) Å**	**<(DHCg) °**
**C1-H1A…Cg2**	0.98	2.80	3.541(7)	133
**C1-H1B…Cg1[a]**	0.98	2.99	3.886(6)	152
**C6-H6…Cg6**	0.95	2.92	3.636(6)	133
**C11-H11B…Cg4**	0.99	2.49	3.415(6)	156
**C13-H13…Cg3**	0.95	2.81	3.598(7)	141
**C17-H17…Cg5**	0.95	2.66	3.557(7)	158
**C46-H46…Cg8[e]**	0.95	2.83	3.762(8)	169

Symmetry transformations used to generate equivalent atoms: [a] 1-x,-y,1-z, [b] -1/2+x,1/2-y,1/2+z, [c] -1+x,y,z, [d] 2-x,-y,1-z, [e] -x,1-y,1-z. Cg1 = centre of gravity of C2 – C7; Cg2 of C12 - C17; Cg3 of C18 – C23; Cg4 of C24 – C29; Cg5 of C30 – C35; Cg6 of C36 – C41; Cg8 of C48 – C53.

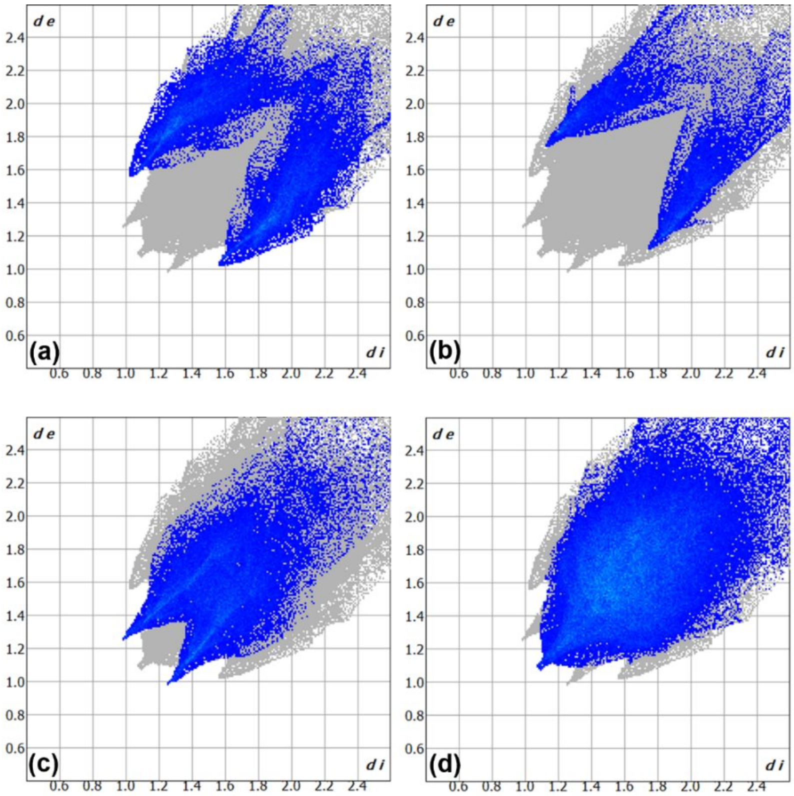

Figure S43: Fingerprint plots of **1a** illustrating the plot resolved into (a) C…H/H…C contacts (17.7%), (b) Cl…H/H…Cl contacts (8.7%), (c) F…H/H…F contacts (19.1%), and (d) H…H contacts (52.9%).

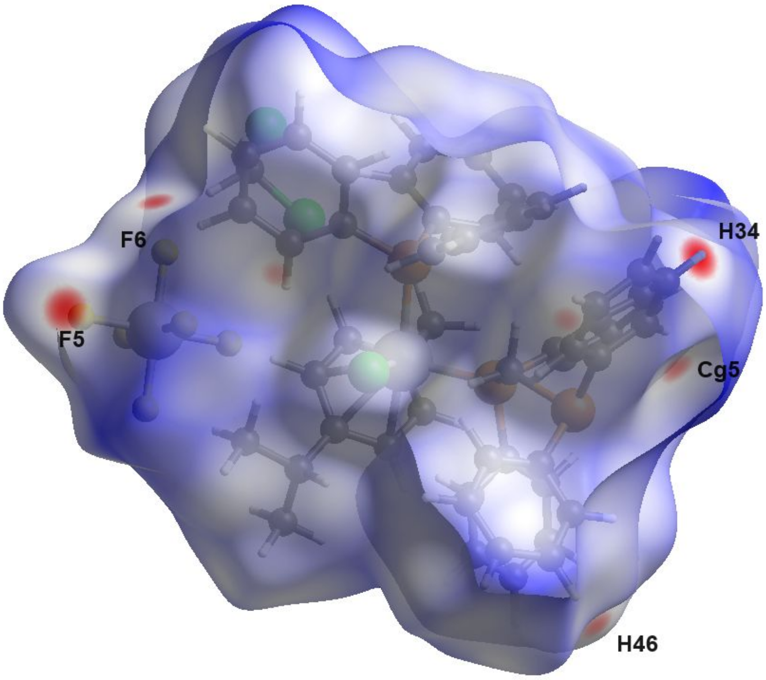

Figure S44: Hirshfeld surface for **1a** mapped with d_{norm} over the range -0.2–2.0 illustrating some of the intermolecular interactions observed.

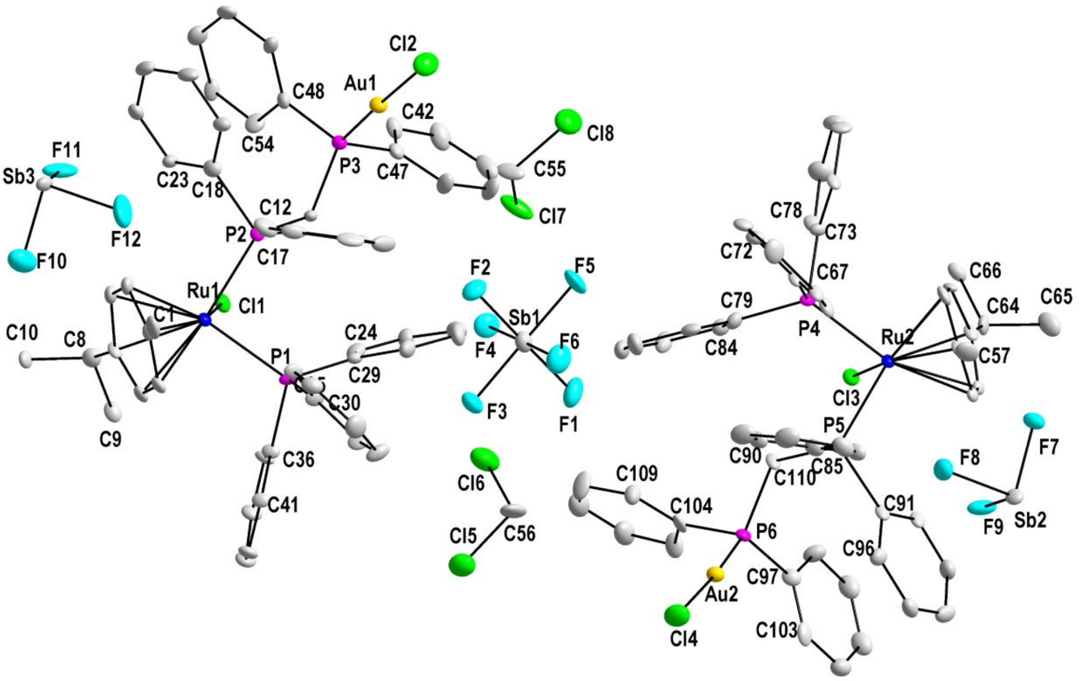

Figure S45. Illustration of the molecular structure of **1b**. Ellipsoids are drawn at 50 % probability, and insignificant labels and hydrogen atoms are omitted for clarity.

Table S4. Selected bond lengths and angles of **1b**.

Bonds	Bond lengths (Å)	Angles	Bond angles (°)
Ru1-Cl1	2.392(3)	P1- Ru1-Cl1	89.2(1)
Ru1-P1	2.367(3)	P2- Ru1-Cl1	87.4(1)
Ru1-P2	2.366(3)	P1- Ru1-P2	93.4(1)
Ru1-Cg1*	1.7892(8)	P2-C11-P3	121.7(6)
P2-C11	1.83(1)	C11-P3-Au1	115.4(4)
P3-C11	1.83(1)	P3-Au1-Cl2	174.8(1)
P3-Au1	2.220(3)	Cg1*-Ru1-P1	125.46(8)
Au1-Cl2	2.287(3)	Cg1*-Ru1-P2	129.9(7)
		Cg1*-Ru1-Cl1	119.46(8)
Ru2-Cl3	2.381(3)	P4- Ru2-Cl3	88.80(10)
Ru2-P4	2.377(3)	P5- Ru2-Cl3	87.44(10)
Ru2-P5	2.369(3)	P4- Ru2-P5	93.58(9)
Ru2-Cg9*	1.792(4)	P5-C110-P6	120.6(6)
P5-C110	1.860(11)	C110-P6-Au2	115.8(4)
P6-C110	1.835(11)	P6-Au2-Cl4	173.67(11)
P6-Au2	2.231(3)	Cg9*-Ru2-P4	124.93(16)
Au2-Cl4	2.285(3)	Cg9*-Ru2-P5	130.28(16)
		Cg9*-Ru2-Cl3	119.70(17)

**Cg1 is the centre of gravity of the C2 - C7 ring of the p-cymene moiety and Cg9 of C58-C63.*

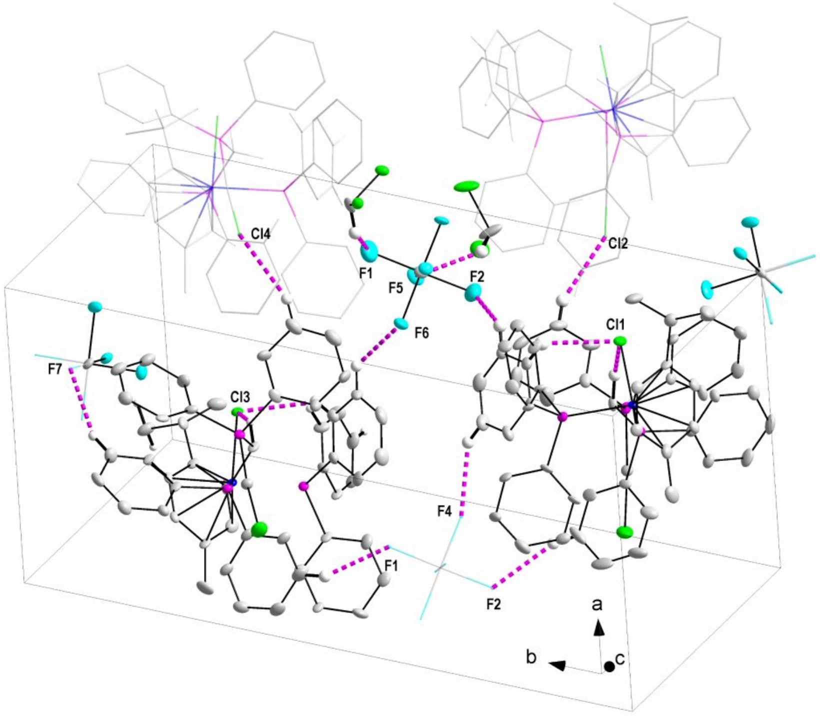

Figure S46. Hydrogen bonding interactions observed in the structure of **1b** (pink dashed lines). Ellipsoids are drawn at 50% probability, and insignificant labels and hydrogen atoms are omitted for clarity.

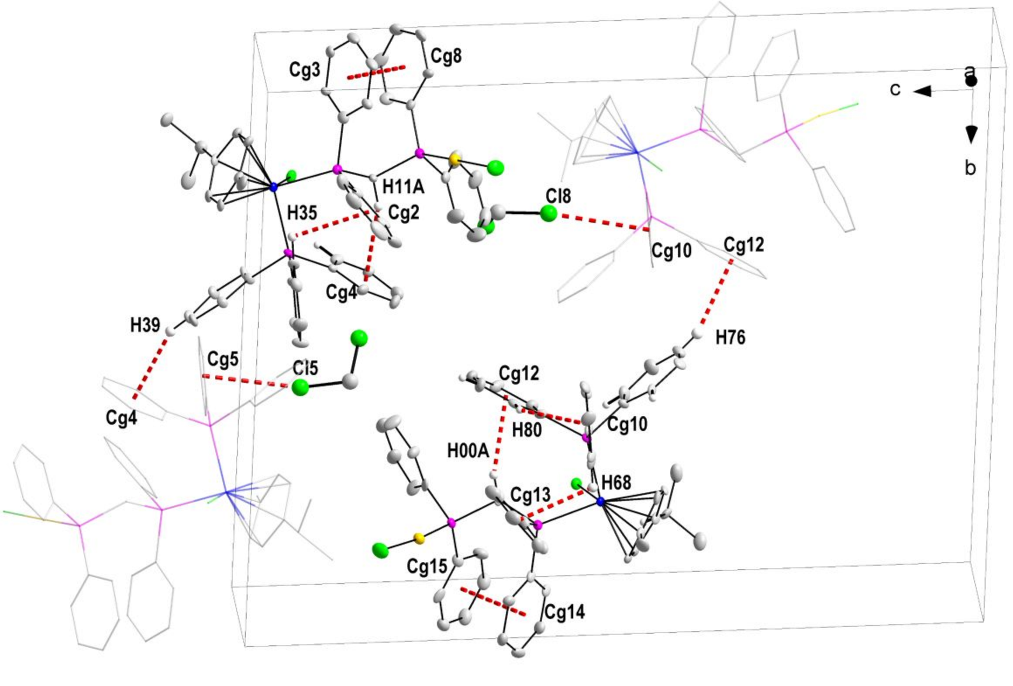

Figure S47. π-interactions observed in the structure of **1b** (red dashed lines). Ellipsoids are drawn at 50% probability, and insignificant labels, counter ions and hydrogen atoms are omitted for clarity.

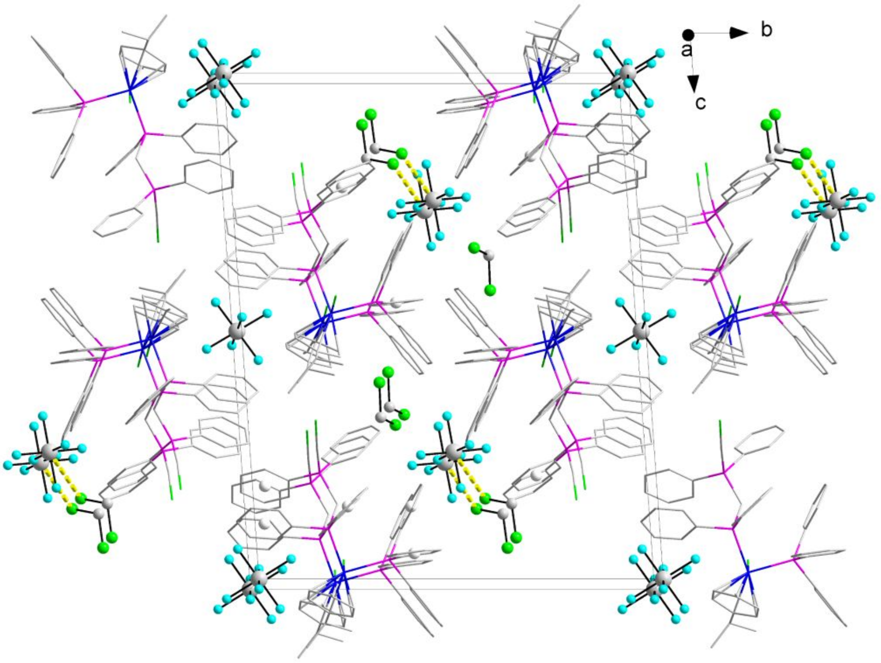

Figure S48. Unit cell packing of **1b**. Ellipsoids are drawn at 50% probability, and labels and hydrogen atoms are omitted for clarity. Yellow dashed lines indicate the short intermolecular contacts between Sb1 and Cl6.

Table S5. Hydrogen bonding and π-interactions observed in the structure of **1b**.

D-H…A	d(D-H) Å	D(H…A) Å	d(D…A) Å	<(DHA) °
**C110-H110B...Cl3**	0.99	2.48	3.23(1)	132
**C11-H11B…Cl1**	0.99	2.48	3.22 (1)	131
**C14-H14...F2[a]**	0.95	2.50	3.37(2)	152
**C26-H26...F4[a]**	0.95	2.45	3.22(2)	137
**C28-H28...F2**	0.95	2.47	3.20(1)	134
**C29-H29...Cl1**	0.95	2.63	3.29(1)	127
**C46-H46...Cl2[b]**	0.95	2.68	3.56(1)	155
**C55-H55A...F5**	0.99	2.48	3.27(2)	136
**C56-H56B...F1**	0.99	2.55	3.22(2)	125
**C83-H83...F6**	0.95	2.40	3.06(2)	126
**C84-H84...Cl3**	0.95	2.67	3.31(1)	125
**C89-H89...F1[a]**	0.95	2.38	3.22(2)	147
**C93-H93...F7[c]**	0.95	2.51	3.41(2)	158
**C106-H106...Cl4[b]**	0.95	2.73	3.62(2)	156
**D-H…Cg**	**d(D-H) Å**	**D(H…Cg) Å**	**d(D…Cg) Å**	**<(DHCg) °**

**C110-H110A…Cg12**	0.99	2.78	3.65(1)	146
**C11-H11A…Cg4**	0.99	2.86	3.74(1)	149
**C35-H35…Cg2**	0.95	2.85	3.23(1)	105
**C39-H39…Cg4^{d}**	0.95	2.75	3.62(1)	153
**C68-H68…Cg13**	0.95	2.87	3.24(1)	104
**C76-H76…Cg12^{e}**	0.95	2.83	3.71(1)	154
**C80-H80…Cg10**	0.95	2.96	3.73(1)	138

**D-X…Cg**	**d(D-X) Å**	**D(X…Cg)**	**d(D…Cg) Å**	**<(DXCg) °**
**C55-Cl8…Cg10^{e}**	1.769(16)	3.535(6)	5.289(16)	171.0(6)
**C56-Cl5…Cg5^{d}**	1.755(13)	3.504(6)	5.221(14)	165.4(5)

**CgX…CgY**	**d(CgX-CgY)**
**Cg3…Cg8**	3.458(7)
**Cg14…Cg15**	3.482(7)

Symmetry transformations used to generate equivalent atoms: ^{a} -1+x,y,z,^{b} 1+x,y,z, ^{c} 1-x,2-y,1-z, ^{d} 1-x,1-y,2-z, ^{e} 1-x,1-y,1-z. Cg2 = centre of gravity of C12 -C17; Cg3 of C18 – C23; Cg4 of C24 – C29; Cg5 of C30 – C35; Cg8 of C48 – C53; Cg10 of C67 – C72; Cg12 of C79 – C84; Cg13 of C85 – C90; Cg14 of C91 – C96; Cg15 of C97 – C103.

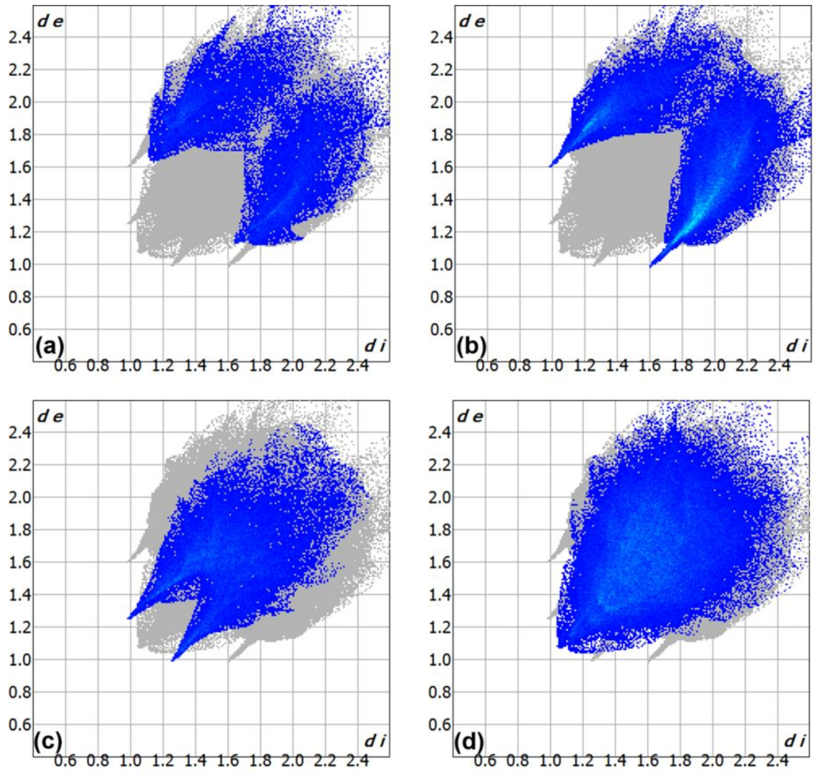

Figure S49. Fingerprint plots of **1b** illustrating the plot resolved into (a) C…H/H…C contacts (12.7%), (b) Cl…H/H…Cl contacts (24.4%), (c) F…H/H…F contacts (17.4%), and (d) H…H contacts (40.5%).

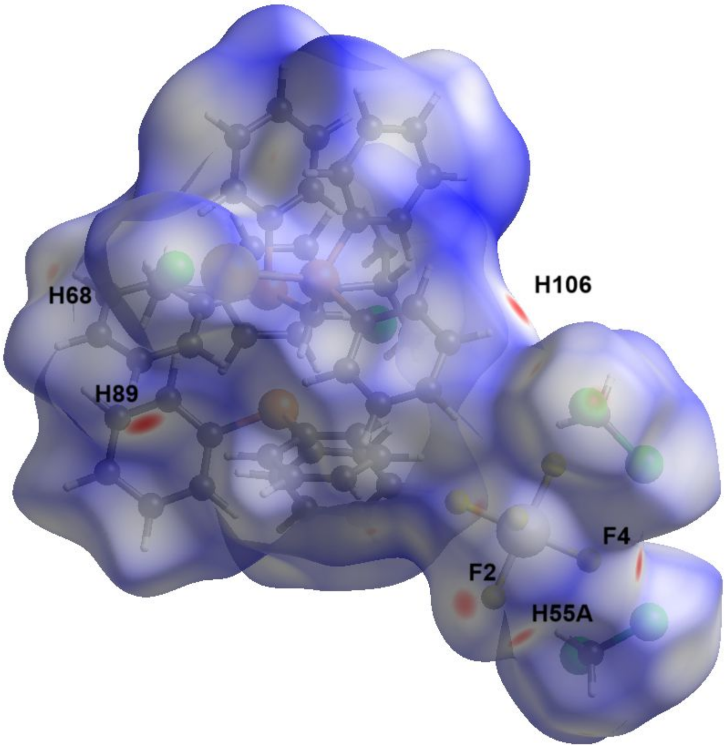

Figure S50: Hirshfeld surface for **1b** mapped with d_{norm} over the range -0.18–1.83 illustrating some of the intermolecular interactions observed.

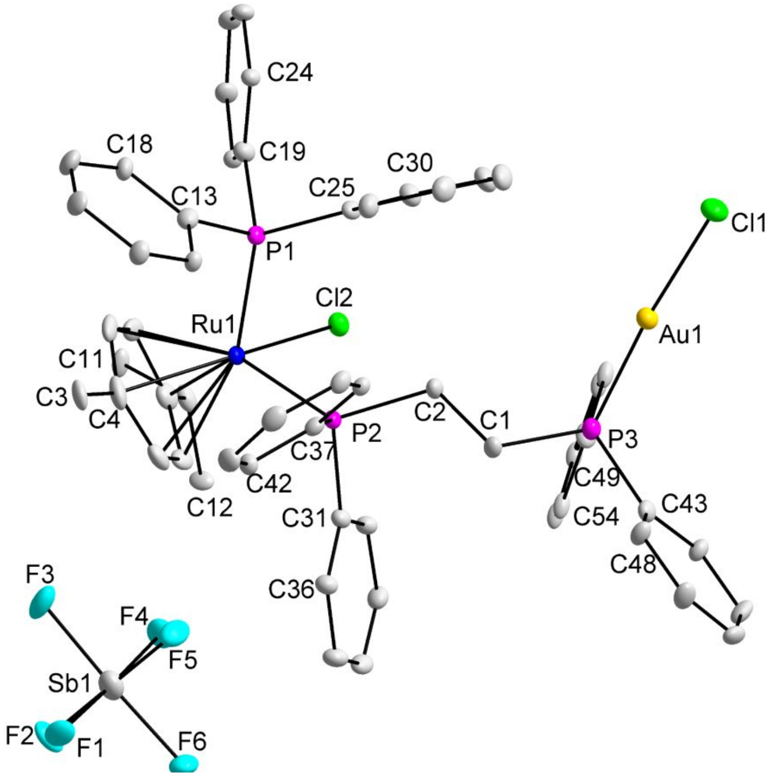

Figure S51. Illustration of the molecular structure of **2b**. Ellipsoids are drawn at 50 % probability, and insignificant labels and hydrogen atoms are omitted for clarity.

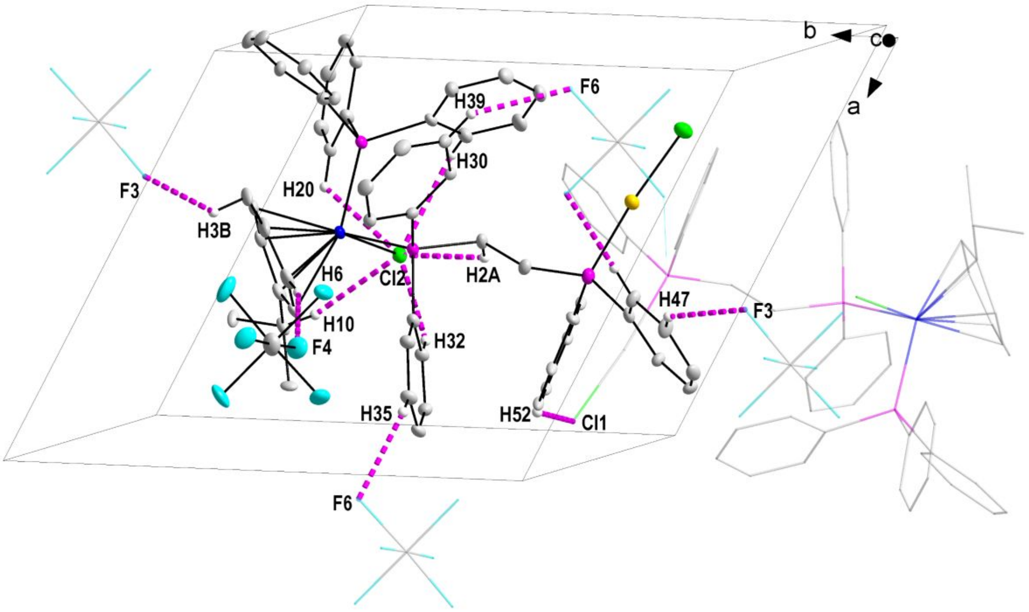

Figure S52. Hydrogen bonding interactions observed in the structure of **2b**. Ellipsoids are drawn at 50% probability, and insignificant labels and H-atoms were omitted for clarity.

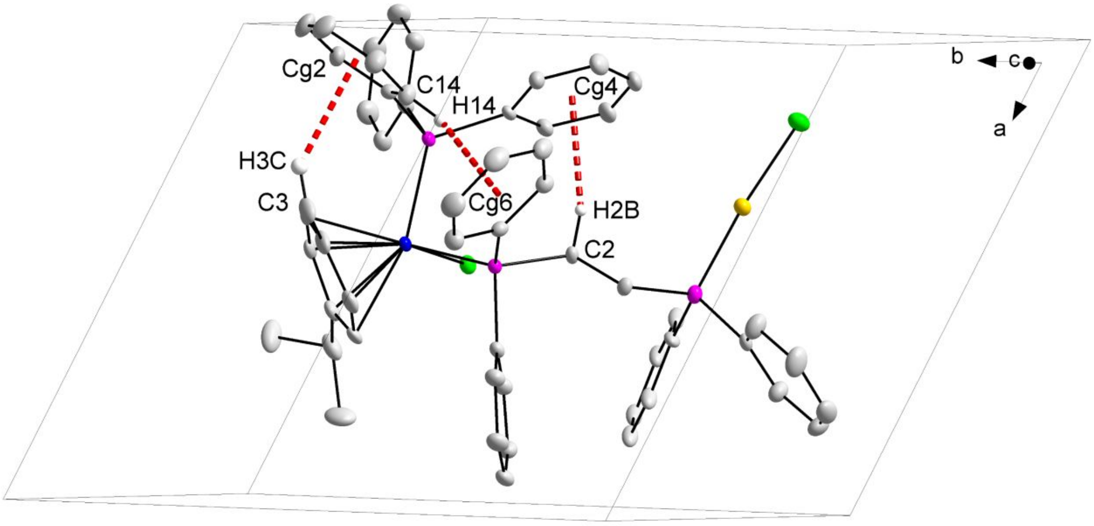

Figure S53. The π-interactions observed in the structure of **2b**. Ellipsoids are drawn at 50% probability, and insignificant labels and H-atoms were omitted for clarity.

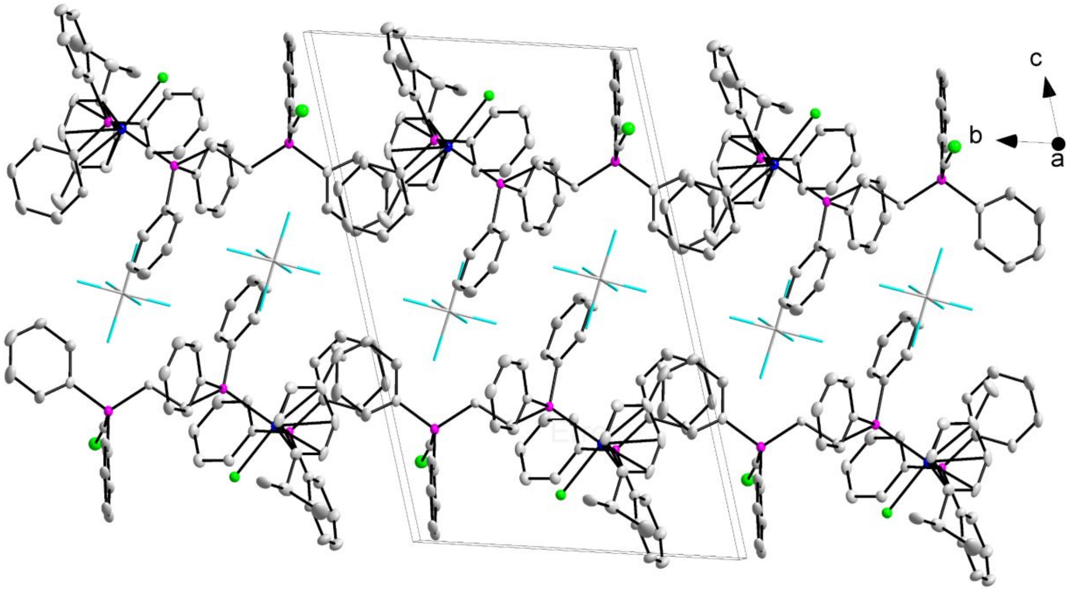

Figure S54. Packing of **2b** in the unit cell, viewed along the a-axis.

Table S6. Selected bond distances and angles of **2b**.

Bonds	Bond distances (Å)	Angles	Bond angles (°)
Ru1-Cl2	2.396(1)	P1- Ru1-Cl2	88.75(5)
Ru1-P1	2.337(1)	P2- Ru1-Cl2	86.33(5)
Ru1-P2	2.364(1)	P1- Ru1-P2	94.41(5)
Ru1-Cg1*	1.7960(6)	Ru1-P2-C2	119.5(2)
P2-C2	1.846(5)	P2-C2-C1	113.9(4)
C2-C1	1.536(7)	C2-C1-P3	111.9(3)
P3-C1	1.827(5)	C1-P3-Au1	117.0(2)
P3-Au1	2.232(2)	P3-Au1-Cl1	175.12(5)
Au1-Cl1	2.293(1)	Cg1*-Ru1-P1	125.70(5)
		Cg1*-Ru1-P2	126.76(4)
		Cg1*-Ru1-Cl2	123.18(4)

**Cg1 is the centre of gravity of the C2 - C7 ring of the p-cymene moiety.*

Table S7. Hydrogen bonding and π-interactions observed in the structure of **2b**.

D-H…A	d(D-H) Å	D(H…A) Å	d(D…A) Å	<(DHA) °
**C2-H2A…Cl2**	0.99	2.72	3.337(6)	121
**C3-H3B…F3[a]**	0.98	2.43	3.17(1)	132

**C6-H6…F4**	0.95	2.51	3.416(8)	159
**C10-H10…Cl2**	1.00	2.78	3.449(9)	125
**C20-H20…Cl2**	0.95	2.75	3.387(7)	125
**C30-H30…Cl2**	0.95	2.82	3.441(7)	124
**C32-H32…Cl2**	0.95	2.78	3.601(6)	145
**C35-H35…F6[b]**	0.95	2.52	3.204(8)	129
**C39-H39…F6[c]**	0.95	2.55	3.374(9)	145
**C47-H47…F3[d]**	0.95	2.54	3.279(9)	134
**C48-H48…F5[c]**	0.95	2.44	3.387(9)	173
**C52-H52…Cl1[e]**	0.95	2.81	3.528(3)	133

**D-H…Cg**	**d(D-H) Å**	**D(H…Cg) Å**	**d(D…Cg) Å**	**<(DHCg) °**
**C2-H2B…Cg4**	0.99	2.58	3.522(7)	159
**C3-H3C…Cg2**	0.98	2.68	3.51(1)	143
**C14-H14…Cg6**	0.95	2.52	3.403(7)	155

Symmetry transformations used to generate equivalent atoms: [a] *1-x,2-y,1-z,* [b] *2-x,1-y,1-z,* [c] *1-x,1-y,1-z,* [d] *x,-1+y,z,* [e] *1-x,-y,2-z. Cg2 = centre of gravity of C13 -C18; Cg4 of C25 – C30; Cg6 of C37 – C42.*

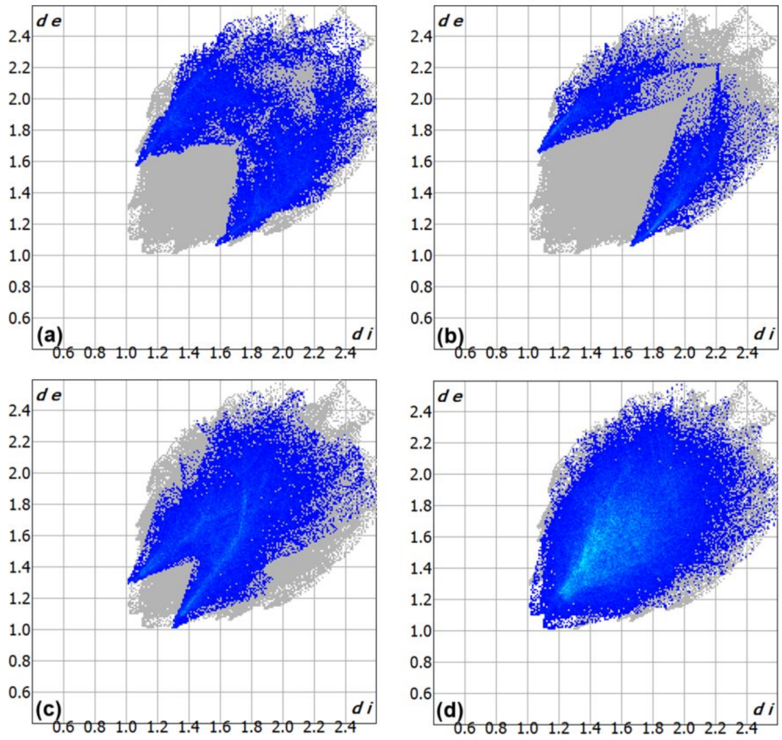

Figure S55. Fingerprint plots of **2b** illustrating the plot resolved into (a) C…H/H…C contacts (14.1%), (b) Cl…H/H…Cl contacts (8.7%), (c) F…H/H…F contacts (20.9%), and (d) H…H contacts (49.2%).

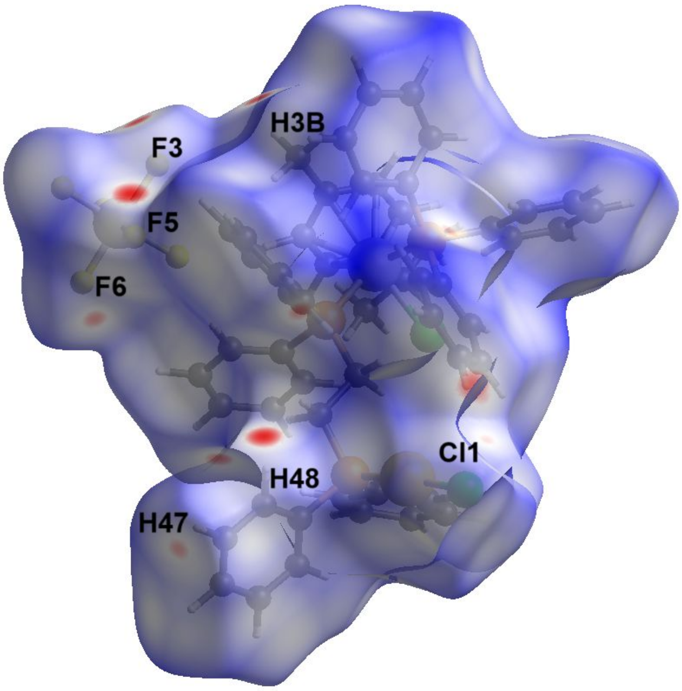

Figure S56. Hirshfeld surface for **2b** mapped with d_{norm} over the range -0.188–1.40 illustrating some of the intermolecular interactions observed.

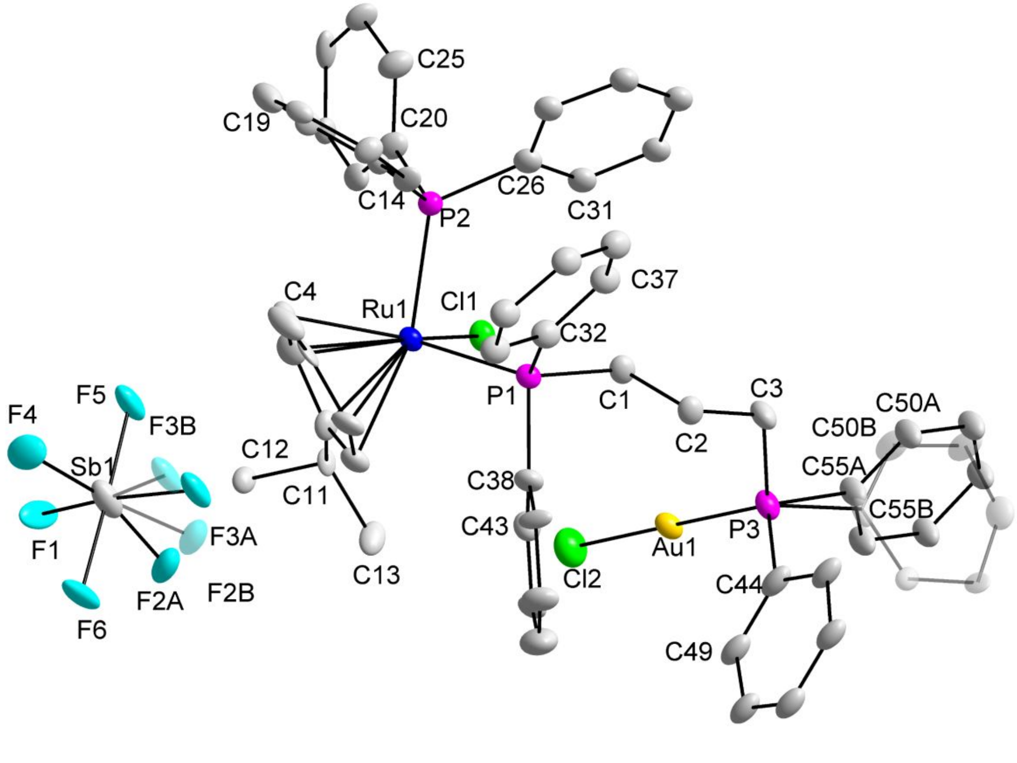

Figure S57. The molecular structure of **3b**. Ellipsoids are drawn at 50% probability, and insignificant labels and hydrogen atoms are omitted for clarity.

Table S8. Selected bond distances and angles of **3b**.

Bonds	Bond lengths (Å)	Angles	Bond angles (°)
Ru1-Cl1	2.389(3)	P1- Ru1-Cl1	88.5(1)
Ru1-P1	2.358(3)	P2- Ru1-Cl1	87.1(1)
Ru1-P2	2.366(3)	P1- Ru1-P2	96.3(1)
Ru1-Cg1*	1.795(1)	Ru1-P2-C1	121.2(4)
P1-C1	1.84(1)	P2-C1-C2	115.7(9)
C2-C1	1.52(2)	C2-C3-P3	112.3(8)
C2-C3	1.54(2)	C3-P3-Au1	115.8(5)
P3-C3	1.81(1)	P3-Au1-Cl2	178.5(2)
P3-Au1	2.226(4)	Cg1*-Ru1-P1	125.16(9)
Au1-Cl2	2.267(4)	Cg1*-Ru1-P2	125.67(8)
		Cg1*-Ru1-Cl1	123.03(9)

**Cg1 is the centre of gravity of the C5 – C10 six carbon ring of the p-cymene moiety.*

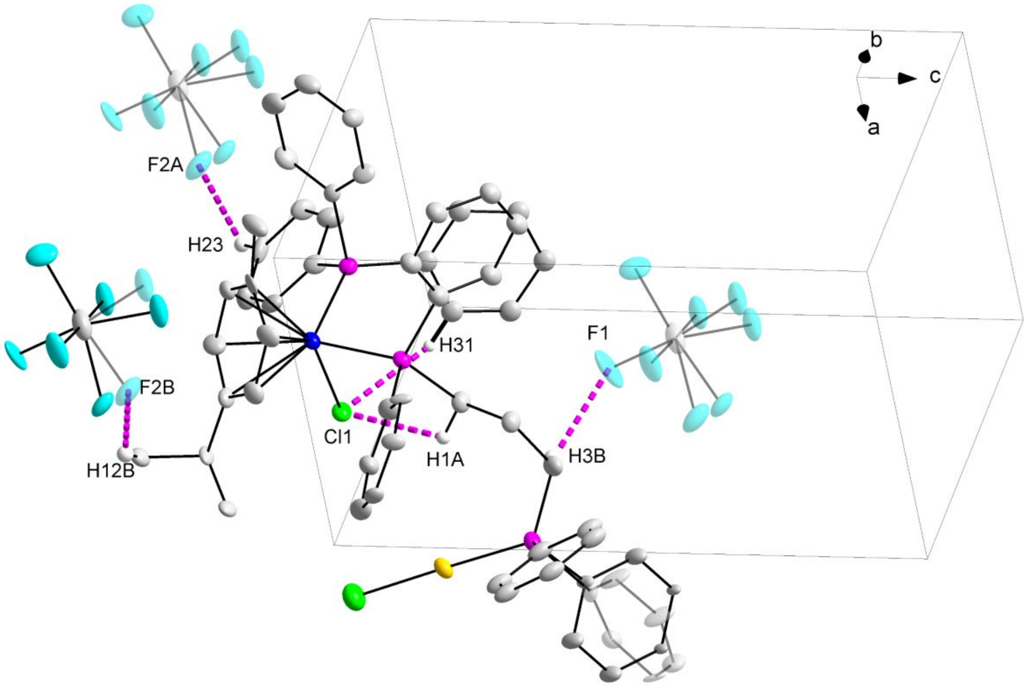

Figure S58. Hydrogen bonding interactions observed in the structure of **3b**. Ellipsoids are drawn at 50% probability, and insignificant labels and hydrogen atoms are omitted for clarity.

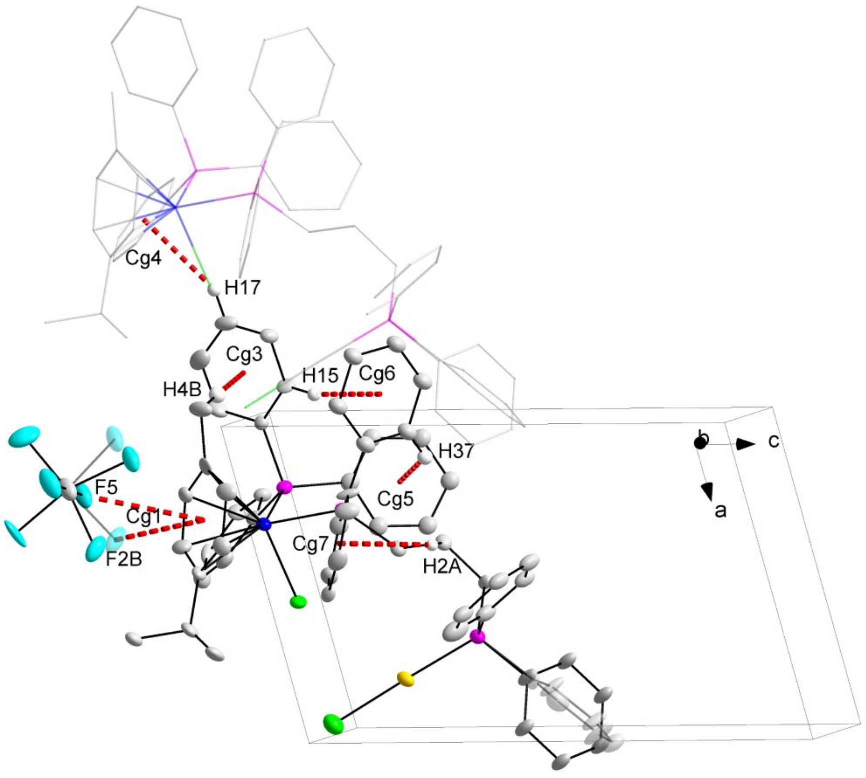

Figure S59. π-interactions observed in the structure of **3b**. Ellipsoids are drawn at 50% probability, and insignificant labels and hydrogen atoms are omitted for clarity.

Table S9. Hydrogen bonding and π-interactions overserved in the structure of **3b**.

**D-H…A**	**d(D-H) Å**	**D(H…A) Å**	**d(D…A) Å**	**<(DHA) °**
**C1-H1A…Cl1**	0.99	2.81	3.33(1)	113
**C3-H3B…F1[a]**	0.99	2.49	3.02(2)	113
**C12-H12B…F2B**	0.98	2.53	3.37(4)	144
**C23-H23…F2A[b]**	0.95	2.42	3.19(6)	138
**C31-H31…Cl1**	0.95	2.65	3.48(1)	146

**D-H…Cg**	**d(D-H) Å**	**D(H…Cg) Å**	**d(D…Cg) Å**	**<(DHCg)**
**C2-H2A…Cg7**	0.99	2.97	3.79(2)	141
**C4-H4B…Cg3**	0.98	2.35	3.31(2)	166
**C15-H15…Cg6**	0.95	2.50	3.42(1)	163
**C17-H17…Cg4[c]**	0.95	2.66	3.54(2)	153
**C37-H37…Cg5**	0.95	2.99	3.58(2)	122

**D-X…Cg**	**d(D-X) Å**	**D(X…Cg) Å**	**d(D…Cg) Å**	**<(DXCg)**
**Sb1-F2B…Cg1**	1.87	3.35(3)	4.230(6)	105(1)

**Sb1-F5…Cg1**	1.87	3.26(1)	4.230(6)	108.1(4)

Symmetry transformations used to generate equivalent atoms: [a] *x,y,1+z,* [b] *x,1+y,z,* [c] *-1+x,y,z. Cg1 = centre of gravity of C5 – C10; Cg3 of C14-19; Cg4 of C20 - C25; Cg5 of C26 - C31; Cg6 of C32 - C37; Cg7 of C38 - C43.*

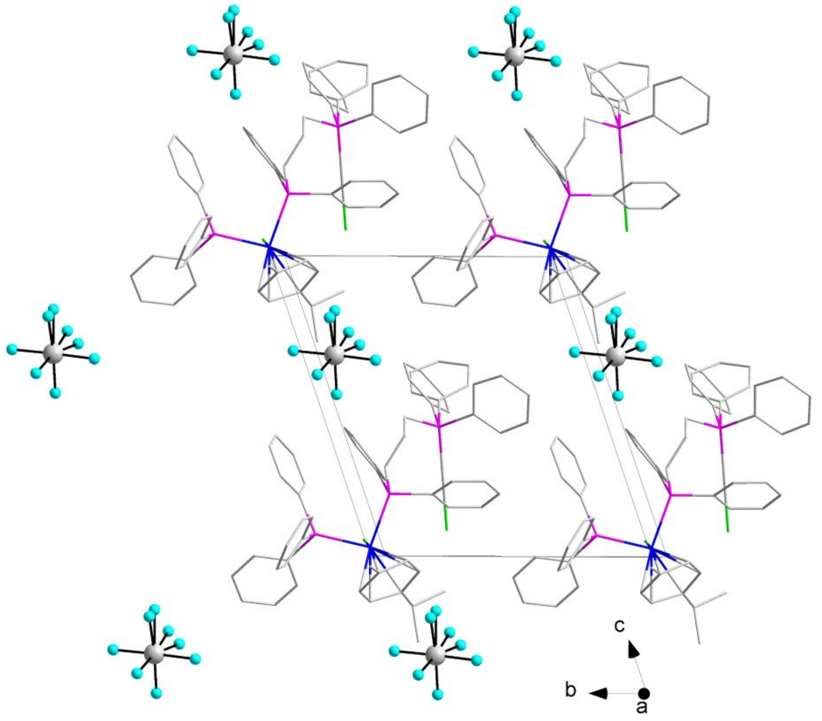

Figure S60. Packing of **3b** in the unit cell, viewed along the a-axis.

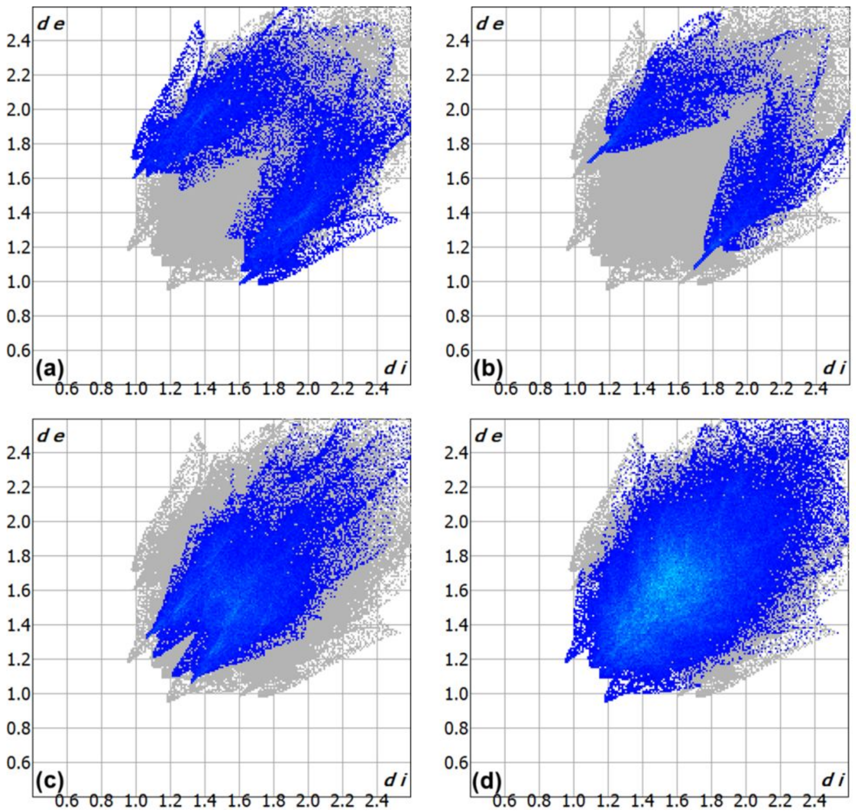

Figure S61. Fingerprint plots of **3b** illustrating the plot resolved into (a) C…H/H…C contacts (14.6%), (b) Cl…H/H…Cl contacts (7.3%), (c) F…H/H…F contacts (20.9%), and (d) H…H contacts (54.5%).

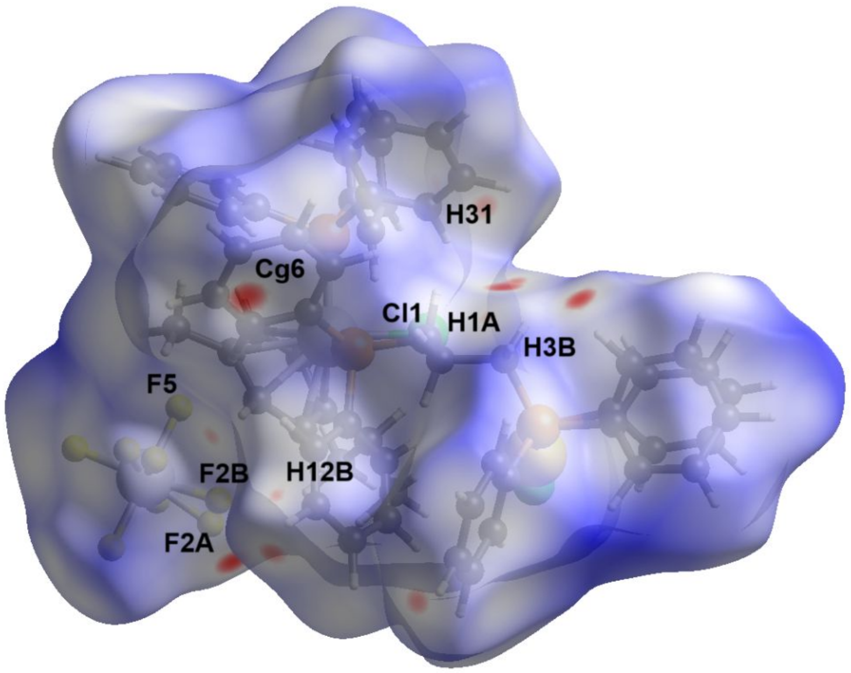

Figure S62. Hirshfeld surface for **3b** mapped with d_{norm} over the range -0.157–1.950 illustrating some of the intermolecular interactions observed
